# Supplementary material for: Early Holocene Scandinavian foragers on a journey to affluence: Mesolithic fish exploitation, seasonal abundance and storage investigated through strontium isotope ratios by laser ablation (LA‐MC-ICP‐MS)
Source: PLoS One. 2021 Jan 20;16(1):e0245222. doi: 10.1371/journal.pone.0245222 (PMC7817046; doi:10.1371/journal.pone.0245222)
Supplement: S1 File — (PDF) [file pone.0245222.s001.pdf]

Supplementary material for Early Holocene Scandinavian foragers on a journey to affluence: Mesolithic fish exploitation, seasonal abundance and storage investigated through strontium isotope ratios by laser ablation (LA-MC-ICP-MS)

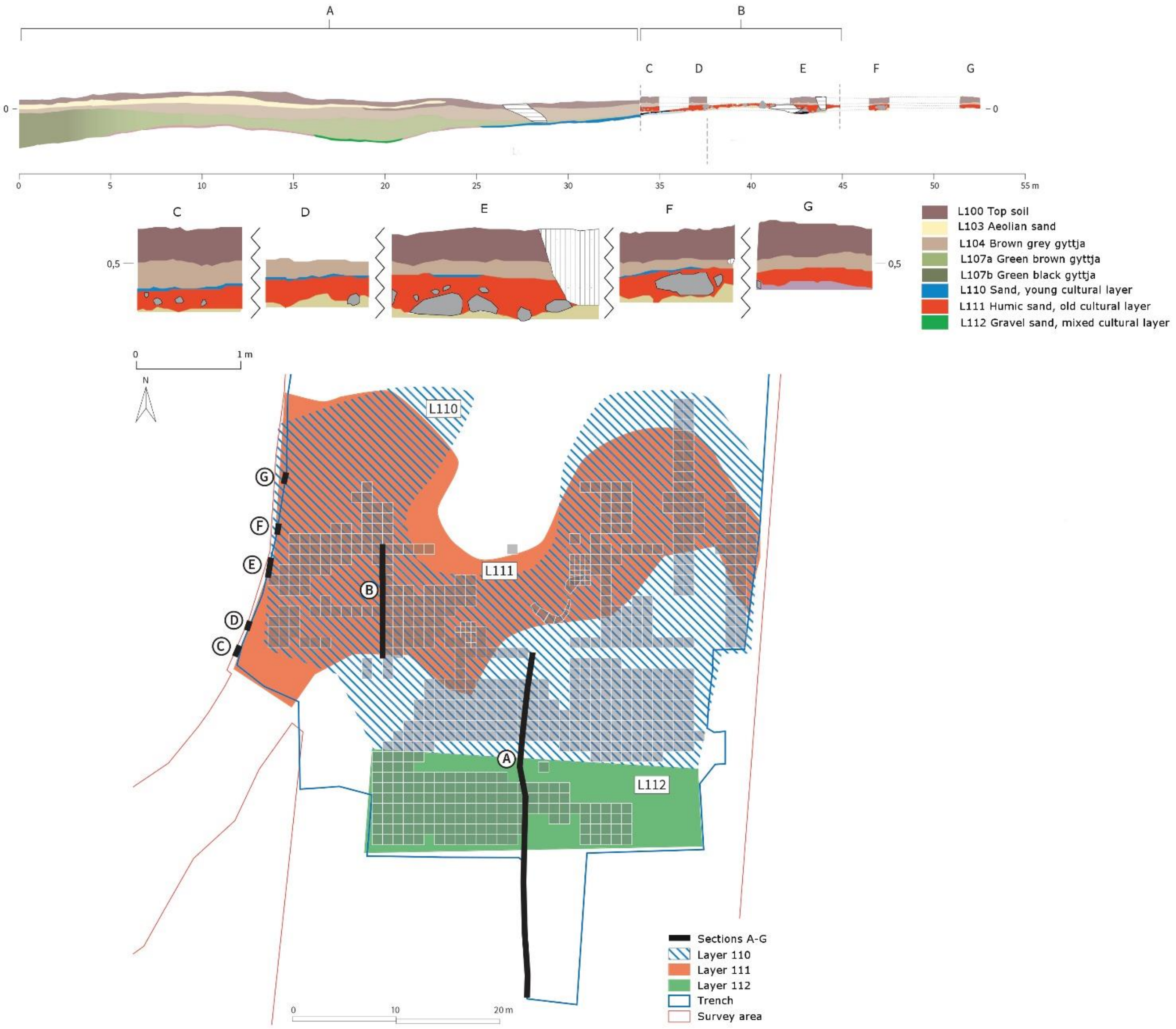

Fig S 1. Basic layout and stratigraphy of the main cultural layers at Norje Sunnansund. L110 and L111 are the two land based cultural layers and L112 is the water deposited cultural layer. The oldest land based layer was recognizable as a dark clayey organic layer with good preservation, (L111), which was deposited on top of the underlying clay deriving from earlier Baltic Sea stages when the entire area was inundated during the transgression following the melting of the ice from the last Ice Age. The youngest land based archaeological layer was a sandy layer covering the older layer (L110). The water deposited layer was a sandy layer deposited directly on top of the underlying clay, on the slope downwards and into the lake (L112). Images originally published in Kjällquist et al (2016) and freely available, with permission from Blekinge Museum, through CC by 4.0 license.

Instrument settings and operating conditions

Table S 1. Instrument settings during the analyses in November 2019

**Instrument Settings** November 2019	
**Mass spectrometer**	Nu plasma (II) MC-ICP-MS
Cooling gas flow rate	13 L/min
Aux gas flow rate	0.9 L/min
Mass resolution	low
Cones	common Ni cones
Torch	glass
**Laser ablation**	ESI NWR193 ArF eximer based laser ablation system
Ar flow rate (Mix Gas)	0.66 L/min
He flow rate	0.50 L/min
Preablation	
Frequency	10 Hz
Translation rate	100 µm/s
Spot-size	150 µm
Fluence	2 J/cm^2
Ablation	
Frequency	25 Hz
Translation rate	5 µm/s
Spotsize	130 µm (shark spine standard with 75µm)
Line raster length	depending on tooth
Fluence	3.7 J/cm^2
Data collection	
Gas background	45 s
Integration	0.5 s
all profiles are from tip to DEJ	
**Corrections**	
Fractionation	factor calculated with accepted ^{86}Sr/^{88}Sr value of 0.1194
Kr	subtracted by measuring gas blank (30 sec) before each measurement
Rb	measured on mass 85, applied on mass 87 (fractionation corrected) assuming ^{87}Rb/^{85}Rb = 0.3861
Ca-Argides/-dimer	measured on mass 82, applied for masses 84, 86, 88
Yb	measured on mass 86.5 ($^{173}Yb^{2+}$), applied for masses 86, 87, 88
Er	measured on mass 83 ($^{166}Er^{2+}$), applied for masses 84, 85
Dy	measured on mass 81.5 ($^{163}Dy^{2+}$), applied for mass 82

Table S 2. Instrument settings during the analyses in February 2020.

**Instrument Settings** February 2020	
**Mass spectrometer**	Nu plasma (II) MC-ICP-MS
Cooling gas flow rate	13 L/min
Aux gas flow rate	0.9 L/min

Mass resolution	low
Cones	common Ni cones
Torch	glass
**Laser ablation**	ESI NWR193 ArF eximer based laser ablation system
Ar flow rate (Mix Gas)	0.7-0.8 L/min
He flow rate	0.45 L/min
Preablation	
Frequency	10 Hz
Translation rate	100 µm/s
Spot-size	150 µm
Fluence	2 J/cm^2
Ablation	
Frequency	25 Hz
Translation rate	5 µm/s
Spotsize	130 µm (shark spine standard with 75µm)
Line raster length	depending on tooth
Fluence	3.7 J/cm^2
Data collection	
Gas background	45 s
Integration	0.5 s
all profiles are from tip to DEJ	
**Corrections**	
Fractionation	factor calculated with accepted $^{86}Sr/^{88}Sr$ value of 0.1194
Kr	subtracted by measuring gas blank (30 sec) before each measurement
Rb	measured on mass 85, applied on mass 87 (fractionation corrected)
	assuming $^{87}Rb/^{85}Rb$ = 0.3861
Ca-Argides/-dimer	measured on mass 82, applied for masses 84, 86, 88
Yb	measured on mass 86.5 ($^{173}Yb^{2+}$), applied for masses 86, 87, 88
Er	measured on mass 83 ($^{166}Er^{2+}$), applied for masses 84, 85
Dy	measured on mass 81.5 ($^{163}Dy^{2+}$), applied for mass 82

Table S 3. Instrument settings during the analyses in September 2020.

**Instrument Settings**<br>September 2020	
Mass spectrometer	Nu plasma (II) MC-ICP-MS
Cooling gas flow rate	13 L/min
Aux gas flow rate	0.9 L/min
Mass resolution	low
Cones	common Ni cones
Torch	glass
Laser ablation	ESI NWR193 ArF eximer based laser ablation system
Ar flow rate (Mix Gas)	0.8 L/min
He flow rate	0.45 L/min
Preablation	
Frequency	10 Hz

Translation rate	100 µm/s
Spotsize	150 µm
Fluence	2 J/cm^2
Ablation	
Frequency	25 Hz
Translation rate	5 µm/s
Spotsize	130 µm (shark spine with 75µm)
Line raster length	depending on tooth
Fluence	3.5 J/cm^2
Data collection	
Gas background	45 s
Integration	0.5 s
all profiles are from tip to DEJ	
Corrections	
Fractionation	factor calculated with accepted $^{86}Sr/^{88}Sr$ value of 0.1194
Kr	substracted by measuring gas blank (30 sec) before each measurement
Rb	measured on mass 85, applied on mass 87 (fractionation corrected) assuming $^{87}Rb/^{85}Rb$ = 0.3861
Ca-Argides/-dimer	measured on mass 82, applied for masses 84, 86, 88
Yb	measured on mass 86.5 ($^{173}Yb^{2+}$), applied for masses 86, 87, 88
Er	measured on mass 83 ($^{166}Er^{2+}$), applied for masses 84, 85
Dy	measured on mass 81.5 ($^{163}Dy^{2+}$), applied for mass 82

Table S 4 Instrument settings during the analyses in October 2020.

**Instrument Settings**<br>October 2020	
Mass spectrometer	Nu plasma (II) MC-ICP-MS
Cooling gas flow rate	13 L/min
Aux gas flow rate	0.9 L/min
Mass resolution	low
Cones	common Ni cones
Torch	glass
Laser ablation	ESI NWR193 ArF eximer based laser ablation system
Ar flow rate (Mix Gas)	0.8 L/min
He flow rate	0.45 L/min
Preablation	
Frequency	10 Hz
Translation rate	100 µm/s
Spotsize	150 µm
Fluence	2 J/cm^2
Ablation	
Frequency	25 Hz
Translation rate	5 µm/s
Spotsize	130 µm (shark spine with 75µm)
Line raster length	depending on tooth

Fluence	3.7 J/cm^2
Data collection	
Gas background	45 s
Integration	0.5 s
all profiles are from tip to DEJ	
**Corrections**	
Fractionation	factor calculated with accepted $^{86}Sr/^{88}Sr$ value of 0.1194
Kr	substracted by measuring gas blank (30 sec) before each measurement
Rb	measured on mass 85, applied on mass 87 (fractionation corrected) assuming $^{87}Rb/^{85}Rb$ = 0.3861
Ca-Argides/-dimer	measured on mass 82, applied for masses 84, 86, 88
Yb	measured on mass 86.5 ($^{173}Yb^{2+}$), applied for masses 86, 87, 88
Er	measured on mass 83 ($^{166}Er^{2+}$), applied for masses 84, 85
Dy	measured on mass 81.5 ($^{163}Dy^{2+}$), applied for mass 82

Table S 5. Instrument operating conditions for standards during the analyses in November 2019.

primary RM: Fin spine 'E_Sp_1915' (Etmopterus Spinax 1915)

LA-ICP-MS

line scans: spotsize: 75 µm

	n	$^{87}Sr/^{86}Sr_{average}$	$2SE_{average}$	external precision 2SD	$^{87}Rb/^{86}Sr_{average}$	2SD	$^{174}Yb^{2+}/^{86}Sr_{average}$	2SD	$^{84}Sr/^{86}Sr_{average}$	$2SE_{average}$	external precision 2SD	total $Sr(V)_{average}$
LA-ICP-MS	204	0.70918	0.00025	0.00028	0.00017	0.00008	0.00003	0.00005	0.05637	0.00006	0.00036	18.46
global seawater (Mokadem et al., 2015)		0.709179		0.000002								

validation RM: Hare tooth inhouse std

LA-ICP-MS

line scans: spotsize: 130 µm

	n	$^{87}Sr/^{86}Sr_{average}$	$2SE_{average}$	external precision 2SD	$^{87}Rb/^{86}Sr_{average}$	2SD	$^{174}Yb^{2+}/^{86}Sr_{average}$	2SD	$^{84}Sr/^{86}Sr_{average}$	$2SE_{average}$	external precision 2SD	total $Sr(V)_{average}$
LA-ICP-MS	125	0.71009	0.00020	0.00026	0.028	0.023	0.00017	0.00009	0.05654	0.00007	0.00059	11.41
solution TIMS		0.709988		0.000015					0.056487		0.000050	

Table S 6. Instrument operating conditions for standards during the analyses in February 2020.

primary RM: Fin spine 'E_Sp_1915' (Etmopterus Spinax 1915)

LA-ICP-MS

line scans: spotsize: 75 µm

	n	$^{87}Sr/^{86}Sr_{average}$	$2SE_{average}$	external precision 2SD	$^{87}Rb/^{86}Sr_{average}$	2SD	$^{174}Yb^{2+}/^{86}Sr_{average}$	2SD	$^{84}Sr/^{86}Sr_{average}$	$2SE_{average}$	external precision 2SD	total $Sr(V)_{average}$
LA-ICP-MS	291	0.70919	0.00023	0.00032	0.00021	0.00014	0.00002	0.00004	0.05625	0.00006	0.00014	19.00
global seawater (Mokadem et al., 2015)		0.709179		0.000002								

validation RM: Hare tooth inhouse std

LA-ICP-MS

line scans: spotsize: 130 µm

	n	$^{87}Sr/^{86}Sr_{average}$	$2SE_{average}$	external precision 2SD	$^{87}Rb/^{86}Sr_{average}$	2SD	$^{174}Yb^{2+}/^{86}Sr_{average}$	2SD	$^{84}Sr/^{86}Sr_{average}$	$2SE_{average}$	external precision 2SD	total $Sr(V)_{average}$
LA-ICP-MS	68	0.71009	0.00018	0.00027	0.031	0.021	0.00017	0.00007	0.05632	0.00009	0.00022	8.01
solution TIMS		0.709988		0.000015					0.056487		0.000050	

Table S 7 Instrument operating conditions for standards during the analyses in September 2020.

primary RM: Fin spine 'E_Sp_1915' (Etmopterus Spinax 1915)

LA-ICP-MS

line scans: spotsize: 75 µm

	n	$^{87}Sr/^{86}Sr$ average	2SE average	external precision 2SD	$^{87}Rb/^{86}Sr$ average	2SD	$^{174}Yb^{2+}/^{86}Sr$ average	2SD	$^{84}Sr/^{86}Sr$ average	2SE average	external precision 2SD	total Sr(V) average
LA-ICP-MS	19	0.70918	0.00027	0.00036	0.00022	0.00007	<DL	-	0.05634	0.00006	0.00009	18.40
global seawater (Mokadem et al., 2015)		0.709179		0.000002								

validation RM: Hare tooth inhouse std

LA-ICP-MS

line scans: spotsize: 130 µm

	n	$^{87}Sr/^{86}Sr$ average	2SE average	external precision 2SD	$^{87}Rb/^{86}Sr$ average	2SD	$^{174}Yb^{2+}/^{86}Sr$ average	2SD	$^{84}Sr/^{86}Sr$ average	$2SE_{average}$	external precision 2SD	total Sr(V) average
LA-ICP-MS	4	0.70991	0.00028	0.00020	0.044	0.013	0.00012	0.00004	0.05636	0.00008	0.00012	9.44
solution TIMS		0.709988		0.000015					0.056487		0.000050	

Table S 8. Instrument operating conditions for standards during the analyses in October 2020.

primary RM: Fin spine 'E_Sp_1915' (Etmopterus Spinax 1915)<br>LA-ICP-MS<br>line scans: spotsize: 75 µm													
	**n**	**$^{87}Sr/^{86}Sr$** average	**2SE** average	**external precision 2SD**	**$^{87}Rb/^{86}Sr$** average	**2SD**	**$^{174}Yb^{2+}/^{86}Sr$** average	**2SD**	**$^{84}Sr/^{86}Sr$** average	**$2SE_{aver}$** age	**external precision 2SD**	**total Sr(V)** average
LA-ICP-MS	33	0.70918	0.00026	0.00034	0.00023	0.000 10	<DL		0.05634	0.00006	0.00010	17.69
global seawater (Mokadem et al., 2015)		0.709179		0.000002								
**validation RM: Hare tooth inhouse std**<br>LA-ICP-MS<br>line scans: spotsize: 130 µm												
	**n**	**$^{87}Sr/^{86}Sr$** average	**2SE** average	**external precision 2SD**	**$^{87}Rb/^{86}Sr$** average	**2SD**	**$^{174}Yb^{2+}/^{86}Sr$** average	**2SD**	**$^{84}Sr/^{86}Sr$** average	**2SE** average	**external precision 2SD**	**total Sr(V)** average
LA-ICP-MS	7	0.70999	0.00028	0.00030	0.039	0.018	0.00013	0.00006	0.05637	0.00008	0.00012	9.58
solution TIMS		0.709988		0.000015					0.056487		0.000050	

Individual tooth data

Each ablated tooth is presented with their unique measurement data (Tab S9-39) followed by a photograph (Fig S2-32 upper) and the individual mobility pattern (Fig S2-32 lower). The division into 'enamel only' (coloured $^{87}Sr/^{86}Sr$ data in the tables and coloured dots in the mobility graphs) and all measurement data is primarily inferred from ocular examination of each tooth. Here 'enamel only' have been determined following close examination of each ablation and its relation to the enamel cover on the tooth. In some cases, it has been difficult to determine if dentine has been ablated or not and on these occasions changes in $^{87}Rb/^{86}Sr$ ratios have been included in the determination. If remaining doubts have existed following this procedure the ablation in question have been circled to highlight a degree of uncertainty.

Cyprinids (Cyprinidae)

Cyprinid 76, Bream (Abramis brama), fermentation pit

Table S 9. $^{87}Sr/^{86}Sr$ measurements for Cyprinid 76. Coloured $^{87}Sr/^{86}Sr$ data marks 'enamel only' ablations as inferred from ocular examination of the close-up photograph and/or through with changes in $^{87}Rb/^{86}Sr$ ratios. Mean $^{84}Sr/^{86}Sr$ for cyprinid 76 is 0.0563±0.0002 (2SD), with accepted value at 0.0565 (Thirlwall, 1991). [1]Propagated from external reproducibility (2SD) obtained from the primary standard during the analytical session combined with the within-run precision of each analysis (2SE) (Iolite Version 2.5). [2] average of total Sr beam intensity in V (sum of ^{84}Sr, ^{86}Sr, ^{87}Sr, ^{88}Sr). [3] estimated from measured Sr intensity in secondary RM using the same ablation conditions (semi-quantitative).

Line number	Sampling time (sec)	$^{87}Sr/^{86}Sr$	2SE	2SD[1]	$^{87}Rb/^{86}Sr$	2SE	$^{174}Yb^{2+}/^{86}Sr$	2SE	Mean Sr-Beam (V)[2]	Estimated Sr concentration (ppm)[3]	$^{84}Sr/^{86}Sr$	2SE	Data points / line
1	36	0.71910	0.00009	0.00018	0.000336	0.000011	0.000146	0.000028	8.4	610	0.056450	0.000059	71
2	39	0.71922	0.00008	0.00018	0.001190	0.000162	0.000144	0.000025	8.5	620	0.056401	0.000065	78
3	43	0.71864	0.00012	0.00020	0.000298	0.000014	0.000142	0.000020	10.1	740	0.056253	0.000050	85
4	39	0.71937	0.00008	0.00018	0.000262	0.000008	0.000128	0.000019	10.5	770	0.056341	0.000054	77
5	43	0.71901	0.00009	0.00018	0.000304	0.000009	0.000073	0.000019	11.8	870	0.056282	0.000051	85
6	36	0.71880	0.00013	0.00021	0.000245	0.000012	0.000124	0.000015	14.2	1040	0.056383	0.000045	73
7	36	0.71890	0.00013	0.00020	0.000258	0.000014	0.000117	0.000019	12.0	870	0.056332	0.000054	73
8	47	0.71888	0.00007	0.00017	0.000224	0.000014	0.000103	0.000014	15.7	1150	0.056306	0.000039	93
9	51	0.71942	0.00008	0.00018	0.001493	0.000368	0.000110	0.000012	14.0	1020	0.056321	0.000038	101
10	50	0.71898	0.00010	0.00019	0.000854	0.000186	0.000114	0.000013	14.2	1040	0.056324	0.000034	100
11	50	0.71934	0.00007	0.00017	0.000201	0.000022	0.000097	0.000015	13.0	950	0.056327	0.000038	99
12	48	0.71911	0.00009	0.00018	0.000758	0.000153	0.000094	0.000012	13.7	1000	0.056313	0.000039	97
13	46	0.71888	0.00010	0.00019	0.000651	0.000049	0.000113	0.000014	15.4	1130	0.056224	0.000039	92
14	44	0.71858	0.00011	0.00019	0.000911	0.000139	0.000080	0.000012	16.6	1220	0.056273	0.000033	89
15	48	0.71837	0.00014	0.00021	0.000412	0.000047	0.000135	0.000012	17.5	1280	0.056206	0.000034	97
16	49	0.71846	0.00011	0.00019	0.000660	0.000055	0.000120	0.000012	16.2	1180	0.056238	0.000025	98
17	44	0.71764	0.00010	0.00019	0.002415	0.000171	0.000120	0.000013	16.7	1220	0.056105	0.000035	87

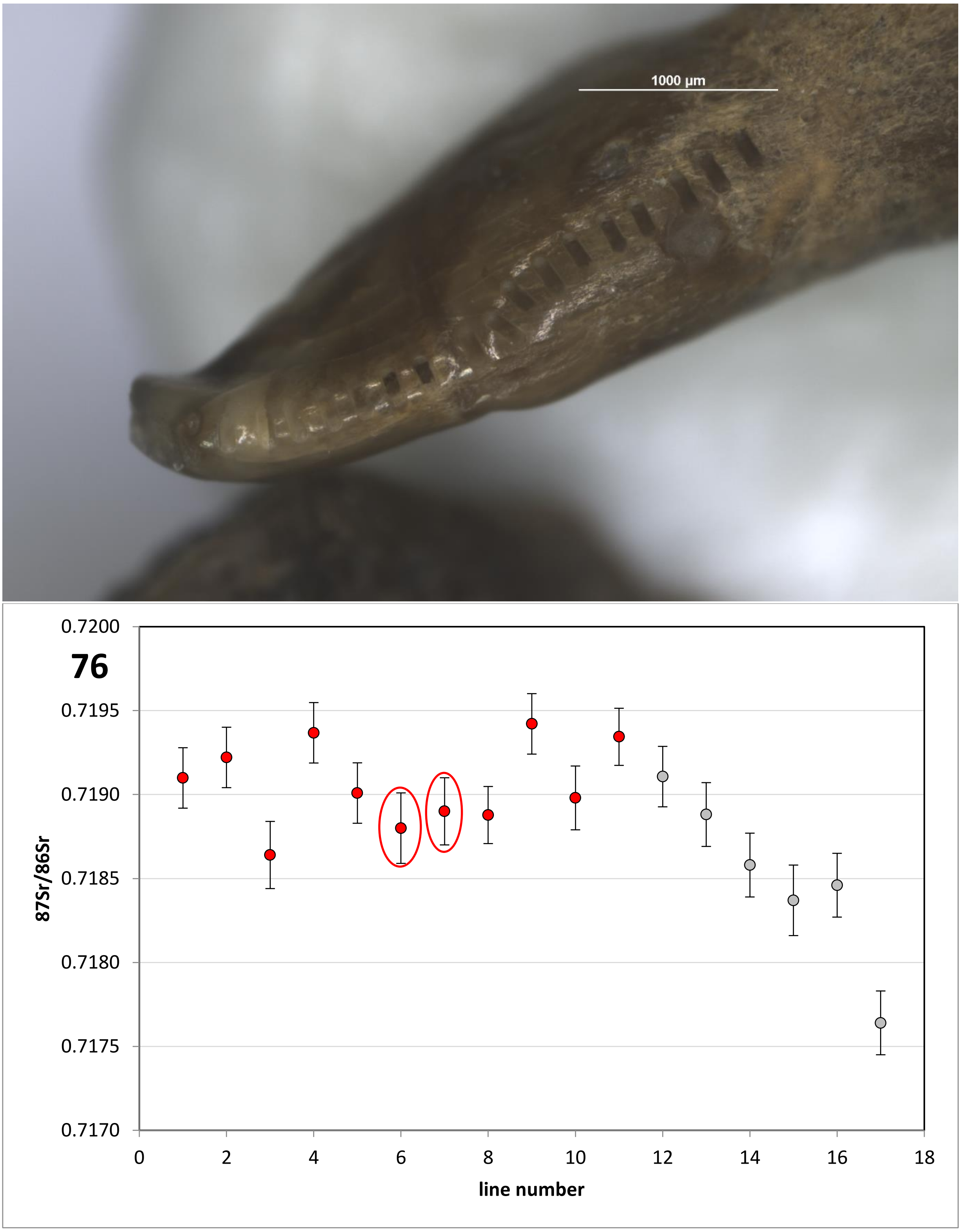

Fig S 2. Close up photograph of ablated cyprinid 76 pharyngeal tooth (upper) and its unique mobility pattern (lower). Red data point indicates 'enamel only' ablation; grey data point indicates enamel-dentine mix and circle around a data point indicate a degree of uncertainty to the assigned category. Photo: Adam Boethius

Cyprinid 77, Rudd (Scardinius erythrophthalmus), fermentation pit

Table S 10. $^{87}Sr/^{86}Sr$ measurements for Cyprinid 77. Coloured $^{87}Sr/^{86}Sr$ data marks 'enamel only' ablations as inferred from ocular examination of the close-up photograph and/or through changes in $^{87}Rb/^{86}Sr$ ratios. Mean $^{84}Sr/^{86}Sr$ for cyprinid 77 is 0.0563±0.0002 (2SD), with accepted value at 0.0565 (Thirlwall, 1991). [1]Propagated from external reproducibility (2SD) obtained from the primary standard during the analytical session combined with the within-run precision of each analysis (2SE) (Iolite Version 2.5). [2] average of total Sr beam intensity in V (sum of ^{84}Sr, ^{86}Sr, ^{87}Sr, ^{88}Sr). [3] estimated from measured Sr intensity in secondary RM using the same ablation conditions (semi-quantitative).

Line number	Sampling time (sec)	$^{87}Sr/^{86}Sr$	2SE	2SD[1]	$^{87}Rb/^{86}Sr$	2SE	$^{174}Yb^{2+}/^{86}Sr$	2SE	Mean Sr-Beam (V)[2]	Estimated Sr concentration (ppm)[3]	$^{84}Sr/^{86}Sr$	2SE	Data points / line
1	30	0.71898	0.00014	0.00036	0.000358	0.000043	0.000134	0.000031	6.1	580	0.056360	0.000100	60
2	27	0.71924	0.00012	0.00035	0.000451	0.000113	0.000158	0.000040	5.3	510	0.056640	0.000150	53
3	22	0.71836	0.00045	0.00056	0.003585	0.000973	0.000096	0.000044	5.6	530	0.056420	0.000170	44
4	31	0.71609	0.00017	0.00037	0.004135	0.000712	0.000057	0.000022	9.2	880	0.056286	0.000067	63
5	28	0.71567	0.00011	0.00035	0.001403	0.000350	0.000080	0.000022	11.8	1120	0.056189	0.000051	56
6	29	0.71578	0.00010	0.00034	0.000468	0.000097	0.000102	0.000019	11.9	1130	0.056280	0.000049	58
7	24	0.71579	0.00010	0.00034	0.000234	0.000012	0.000091	0.000023	11.7	1110	0.056273	0.000070	49
8	29	0.71622	0.00010	0.00034	0.000251	0.000032	0.000073	0.000018	12.5	1190	0.056281	0.000044	58
9	36	0.71634	0.00009	0.00034	0.000180	0.000005	0.000086	0.000016	13.0	1240	0.056257	0.000050	72
10	30	0.71646	0.00009	0.00034	0.000165	0.000006	0.000105	0.000020	12.4	1180	0.056232	0.000054	60
11	34	0.71698	0.00009	0.00034	0.000257	0.000014	0.000088	0.000016	12.0	1140	0.056302	0.000047	67
12	38	0.71657	0.00009	0.00034	0.000426	0.000022	0.000088	0.000016	12.6	1200	0.056341	0.000048	76

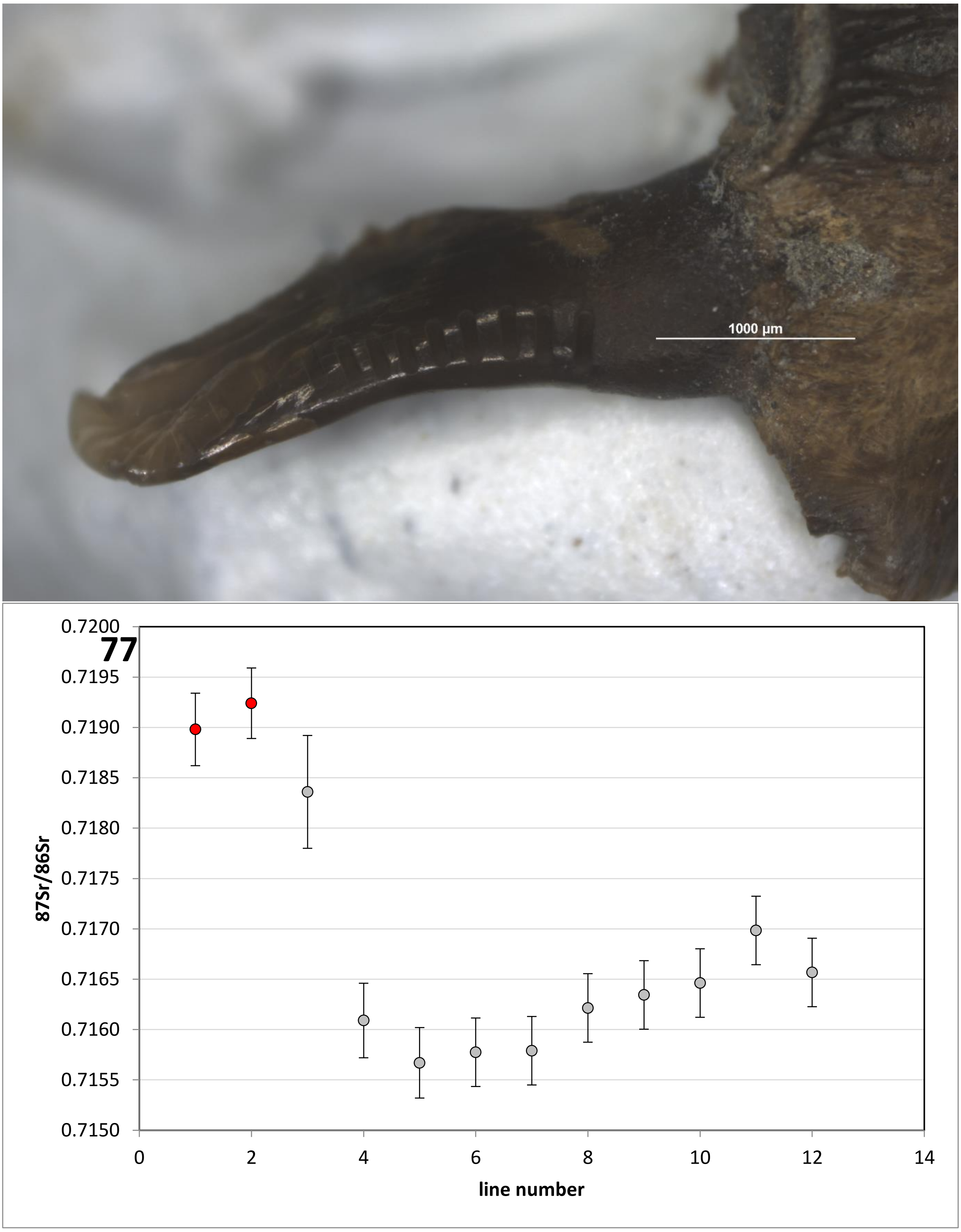

Fig S 3. Close up photograph of ablated cyprinid 77 pharyngeal tooth (upper) and its unique mobility pattern (lower). Red data point indicates 'enamel only' ablation; grey data point indicates enamel-dentine. Photo: Adam Boethius

Cyprinid 78, Dace (Leuciscus leuciscus), fermentation pit

Table S 11. $^{87}Sr/^{86}Sr$ measurements for Cyprinid 78. Coloured $^{87}Sr/^{86}Sr$ data marks 'enamel only' ablations as inferred from ocular examination of the close-up photograph and/or through changes in $^{87}Rb/^{86}Sr$ ratios. Mean $^{84}Sr/^{86}Sr$ for cyprinid 78 is 0.0563±0.0001 (2SD), with accepted value at 0.0565 (Thirlwall, 1991). [1]Propagated from external reproducibility (2SD) obtained from the primary standard during the analytical session combined with the within-run precision of each analysis (2SE) (Iolite Version 2.5). [2] average of total Sr beam intensity in V (sum of ^{84}Sr, ^{86}Sr, ^{87}Sr, ^{88}Sr). [3] estimated from measured Sr intensity in secondary RM using the same ablation conditions (semi-quantitative).

Line number	Sampling time (sec)	$^{87}Sr/^{86}Sr$	2SE	2SD[1]	$^{87}Rb/^{86}Sr$	2SE	$^{174}Yb^{2+}/^{86}Sr$	2SE	Mean Sr-Beam (V)[2]	Estimated Sr concentration (ppm)[3]	$^{84}Sr/^{86}Sr$	2SE	Data points / line
1	17	0.71899	0.00021	0.00039	0.000645	0.000088	0.000068	0.000071	4.3	410	0.056280	0.000210	34
2	17	0.71919	0.00021	0.00039	0.000604	0.000089	0.000035	0.000042	6.8	650	0.056290	0.000110	33
3	17	0.71901	0.00024	0.00041	0.000588	0.000093	0.000079	0.000039	9.0	850	0.056325	0.000092	34
4	18	0.71896	0.00021	0.00039	0.000587	0.000135	0.000120	0.000026	10.9	1040	0.056259	0.000071	36
5	13	0.71933	0.00021	0.00039	0.000111	0.000018	0.000125	0.000037	10.4	990	0.056306	0.000071	27
6	25	0.71930	0.00012	0.00035	0.000178	0.000012	0.000058	0.000026	9.8	930	0.056299	0.000070	50
7	31	0.71905	0.00012	0.00035	0.000140	0.000013	0.000094	0.000022	10.1	960	0.056310	0.000072	62
8	31	0.71899	0.00012	0.00035	0.000149	0.000010	0.000087	0.000023	10.1	960	0.056353	0.000068	61
9	24	0.71887	0.00017	0.00037	0.000178	0.000034	0.000129	0.000030	10.4	990	0.056354	0.000075	47
10	16	0.71940	0.00018	0.00038	0.000085	0.000013	0.000086	0.000040	9.8	930	0.056289	0.000096	32

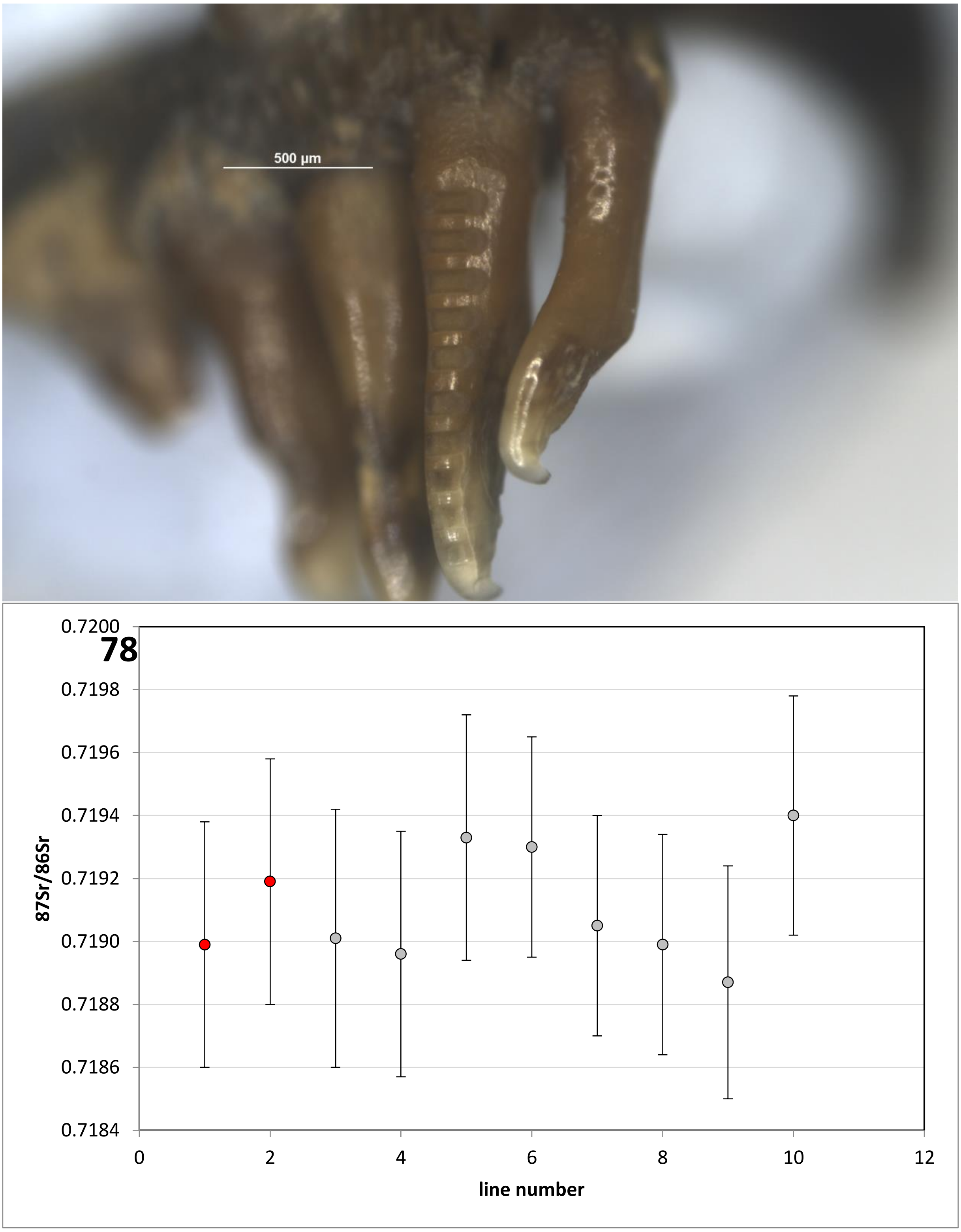

Fig S 4. Close up photograph of ablated cyprinid 78 pharyngeal tooth (upper) and its unique mobility pattern (lower). Red data point indicates 'enamel only' ablation; grey data point indicates enamel-dentine mix. Photo: Adam Boethius

Cyprinid 79, Roach (Rutilus rutilus), fermentation pit

Table S 12. $^{87}Sr/^{86}Sr$ measurements for Cyprinid 79. Coloured $^{87}Sr/^{86}Sr$ data marks 'enamel only' ablations as inferred from ocular examination of the close-up photograph and/or through changes in $^{87}Rb/^{86}Sr$ ratios. Mean $^{84}Sr/^{86}Sr$ for cyprinid 79 is 0.0563±0.0002 (2SD), with accepted value at 0.0565 (Thirlwall, 1991). [1]Propagated from external reproducibility (2SD) obtained from the primary standard during the analytical session combined with the within-run precision of each analysis (2SE) (Iolite Version 2.5). [2] average of total Sr beam intensity in V (sum of ^{84}Sr, ^{86}Sr, ^{87}Sr, ^{88}Sr). [3] estimated from measured Sr intensity in secondary RM using the same ablation conditions (semi-quantitative).

Line number	Sampling time (sec)	$^{87}Sr/^{86}Sr$	2SE	2SD[1]	$^{87}Rb/^{86}Sr$	2SE	$^{174}Yb^{2+}/^{86}Sr$	2SE	Mean Sr-Beam (V)[2]	Estimated Sr concentration (ppm)[3]	$^{84}Sr/^{86}Sr$	2SE	Data points / line
1	25	0.72006	0.00022	0.00040	0.000663	0.000104	0.000160	0.000046	4.9	460	0.056470	0.000190	50
2	29	0.71970	0.00012	0.00035	0.000629	0.000178	0.000122	0.000043	5.7	540	0.056500	0.000110	57
3	27	0.71915	0.00016	0.00037	0.000796	0.000221	0.000092	0.000033	6.7	640	0.056536	0.000082	54
4	28	0.71840	0.00012	0.00035	0.000727	0.000149	0.000071	0.000025	8.0	760	0.056348	0.000088	56
5	29	0.71814	0.00010	0.00035	0.000236	0.000015	0.000098	0.000026	9.1	860	0.056222	0.000087	57
6	38	0.71820	0.00010	0.00035	0.000260	0.000019	0.000085	0.000021	9.6	910	0.056283	0.000059	77
7	29	0.71783	0.00015	0.00036	0.000316	0.000016	0.000103	0.000021	9.9	950	0.056230	0.000069	59
8	34	0.71779	0.00012	0.00035	0.000400	0.000025	0.000111	0.000024	9.2	880	0.056286	0.000078	69
9	32	0.71781	0.00017	0.00037	0.001001	0.000071	0.000081	0.000023	9.2	880	0.056309	0.000090	64
10	33	0.71850	0.00016	0.00037	0.000625	0.000046	0.000076	0.000027	8.2	780	0.056299	0.000073	65

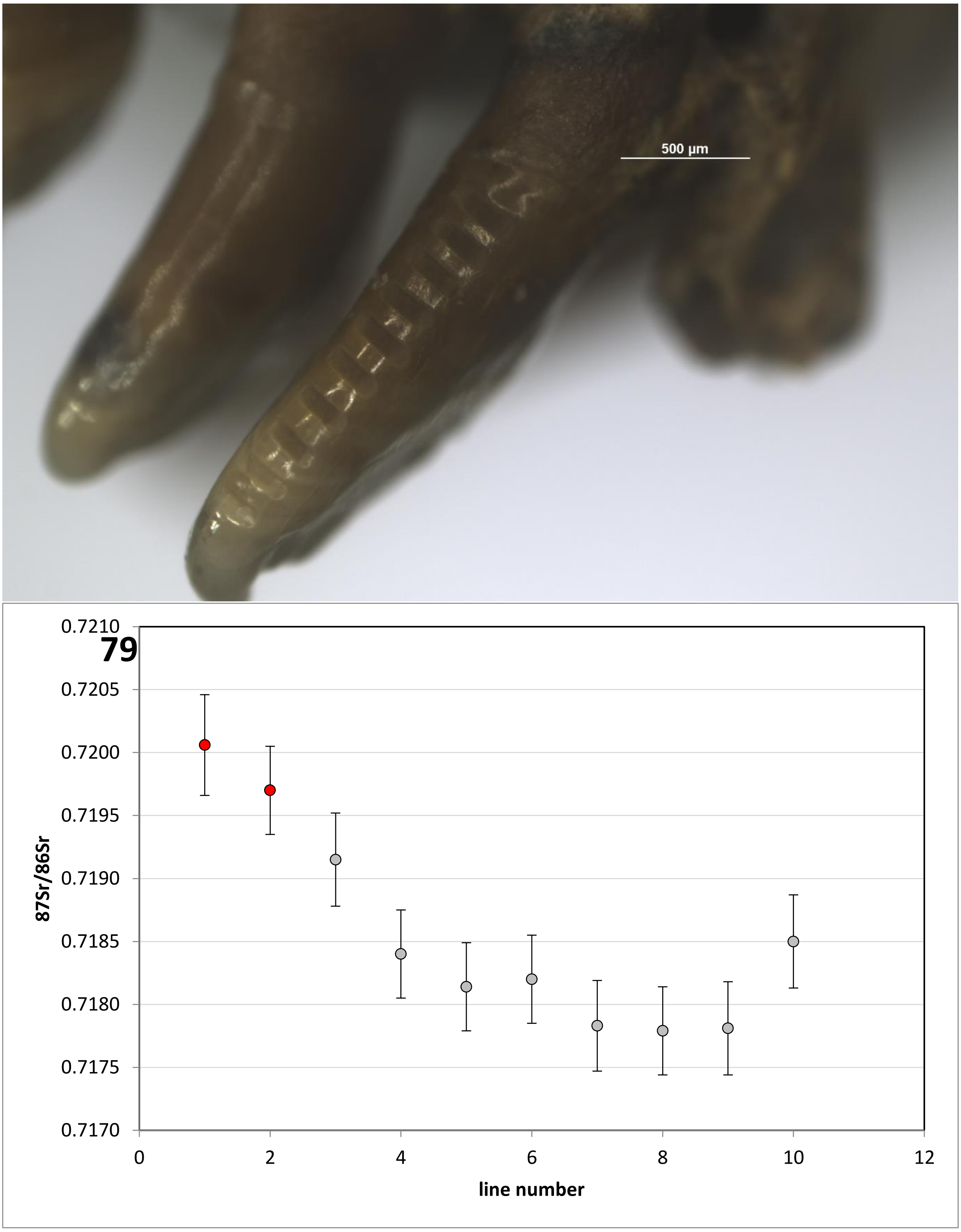

Fig S 5. Close up photograph of ablated cyprinid 79 pharyngeal tooth (upper) and its unique mobility pattern (lower). Red data point indicates 'enamel only' ablation; grey data point indicates enamel-dentine mix. Photo: Adam Boethius

Cyprinid 80, Roach (Rutilus rutilus), fermentation pit

Table S 13. $^{87}Sr/^{86}Sr$ measurements for Cyprinid 80. Coloured $^{87}Sr/^{86}Sr$ data marks 'enamel only' ablations as inferred from ocular examination of the close-up photograph and/or through changes in $^{87}Rb/^{86}Sr$ ratios. Mean $^{84}Sr/^{86}Sr$ for cyprinid 80 is 0.0563±0.0001 (2SD), with accepted value at 0.0565 (Thirlwall, 1991). [1]Propagated from external reproducibility (2SD) obtained from the primary standard during the analytical session combined with the within-run precision of each analysis (2SE) (Iolite Version 2.5). [2] average of total Sr beam intensity in V (sum of ^{84}Sr, ^{86}Sr, ^{87}Sr, ^{88}Sr). [3] estimated from measured Sr intensity in secondary RM using the same ablation conditions (semi-quantitative).

Line number	Sampling time (sec)	$^{87}Sr/^{86}Sr$	2SE	2SD[1]	$^{87}Rb/^{86}Sr$	2SE	$^{174}Yb^{2+}/^{86}Sr$	2SE	Mean Sr-Beam (V)[2]	Estimated Sr concentration (ppm)[3]	$^{84}Sr/^{86}Sr$	2SE	Data points / line
1	45	0.71960	0.00006	0.00024	0.000647	0.000013	0.000169	0.000014	11.0	550	0.056383	0.000048	89
2	43	0.71927	0.00007	0.00025	0.000725	0.000018	0.000157	0.000018	12.2	610	0.056261	0.000052	86
3	35	0.71937	0.00009	0.00025	0.001484	0.000313	0.000164	0.000014	14.4	720	0.056368	0.000046	70
4	55	0.71964	0.00005	0.00024	0.000605	0.000045	0.000162	0.000013	13.4	670	0.056379	0.000037	110
5	53	0.71943	0.00005	0.00024	0.000610	0.000051	0.000090	0.000013	14.6	730	0.056365	0.000032	106
6	49	0.71858	0.00007	0.00025	0.000411	0.000017	0.000123	0.000012	17.2	860	0.056371	0.000028	98
7	48	0.71724	0.00004	0.00024	0.000399	0.000084	0.000103	0.000009	21.3	1060	0.056316	0.000025	95
8	46	0.71733	0.00005	0.00024	0.000420	0.000052	0.000112	0.000008	20.2	1010	0.056318	0.000022	92
9	46	0.71801	0.00004	0.00024	0.000595	0.000074	0.000090	0.000008	22.6	1120	0.056356	0.000022	93
10	51	0.71692	0.00007	0.00025	0.000215	0.000015	0.000088	0.000007	26.5	1320	0.056265	0.000021	102
11	56	0.71662	0.00005	0.00024	0.000176	0.000009	0.000068	0.000008	24.8	1230	0.056288	0.000023	111
12	49	0.71681	0.00004	0.00024	0.000157	0.000004	0.000079	0.000008	23.7	1180	0.056284	0.000022	98
13	54	0.71744	0.00004	0.00024	0.000143	0.000003	0.000079	0.000006	26.3	1310	0.056266	0.000020	108
14	51	0.71815	0.00005	0.00024	0.000165	0.000004	0.000094	0.000007	24.6	1220	0.056283	0.000024	102

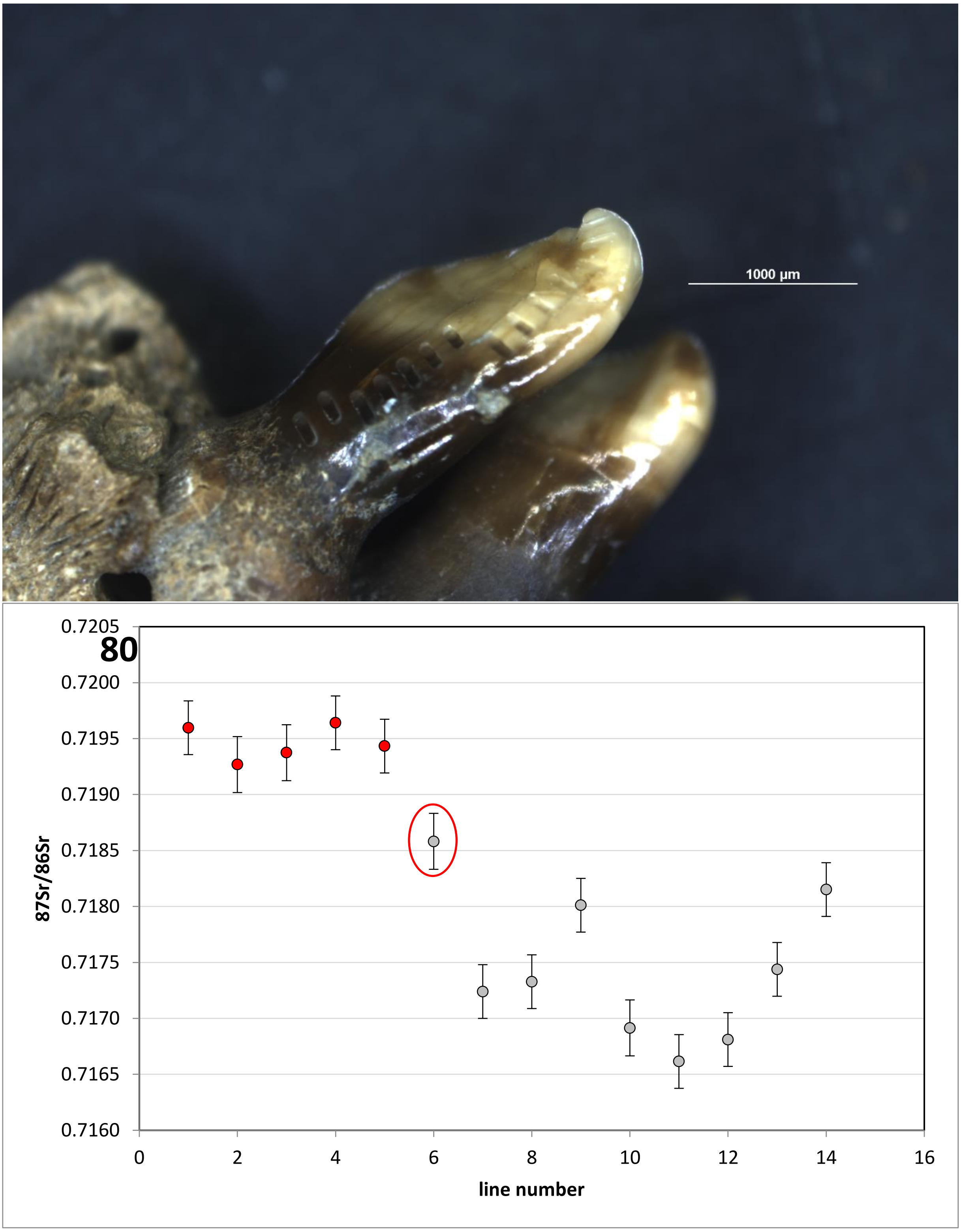

Fig S 6. Close up photograph of ablated cyprinid 80 pharyngeal tooth (upper) and its unique mobility pattern (lower). Red data point indicates ‘enamel only’ ablation; grey data point indicates enamel-dentine mix and circle around a data point indicate a degree of uncertainty to the assigned category. Photo: Adam Boethius

Cyprinid 81, Roach (Rutilus rutilus), fermentation pit

Table S 14. $^{87}Sr/^{86}Sr$ measurements for Cyprinid 81. Coloured $^{87}Sr/^{86}Sr$ data marks ‘enamel only’ ablations as inferred from ocular examination of the close-up photograph and/or through changes in $^{87}Rb/^{86}Sr$ ratios. Mean $^{84}Sr/^{86}Sr$ for cyprinid 81 is 0.0563±0.0001 (2SD), with accepted value at 0.0565 (Thirlwall, 1991). [1]Propagated from external reproducibility (2SD) obtained from the primary standard during the analytical session combined with the within-run precision of each analysis (2SE) (Iolite Version 2.5). [2] average of total Sr beam intensity in V (sum of ^{84}Sr, ^{86}Sr, ^{87}Sr, ^{88}Sr). [3] estimated from measured Sr intensity in secondary RM using the same ablation conditions (semi-quantitative).

Line number	Sampling time (sec)	$^{87}Sr/^{86}Sr$	2SE	2SD[1]	$^{87}Rb/^{86}Sr$	2SE	$^{174}Yb^{2+}/^{86}Sr$	2SE	Mean Sr-Beam (V)[2]	Estimated Sr concentration (ppm)[3]	$^{84}Sr/^{86}Sr$	2SE	Data points / line
1	44	0.71916	0.00007	0.00025	0.000401	0.000011	0.000142	0.000020	10.0	500	0.056293	0.000056	89
2	49	0.71892	0.00005	0.00024	0.000409	0.000011	0.000152	0.000017	11.4	570	0.056188	0.000045	98
3	64	0.71887	0.00004	0.00024	0.000462	0.000007	0.000145	0.000015	11.6	580	0.056377	0.000039	128
4	62	0.71894	0.00004	0.00024	0.000235	0.000010	0.000107	0.000012	14.7	730	0.056348	0.000032	124
5	59	0.71921	0.00004	0.00024	0.000492	0.000042	0.000116	0.000010	16.8	840	0.056351	0.000035	118
6	59	0.71954	0.00006	0.00024	0.000660	0.000085	0.000092	0.000011	17.5	870	0.056400	0.000029	117
7	50	0.71927	0.00007	0.00025	0.000472	0.000056	0.000094	0.000010	18.7	930	0.056361	0.000033	100
8	43	0.71888	0.00005	0.00024	0.000229	0.000010	0.000086	0.000009	23.5	1170	0.056381	0.000027	86
9	43	0.71829	0.00006	0.00024	0.000150	0.000007	0.000079	0.000007	32.7	1630	0.056260	0.000018	86

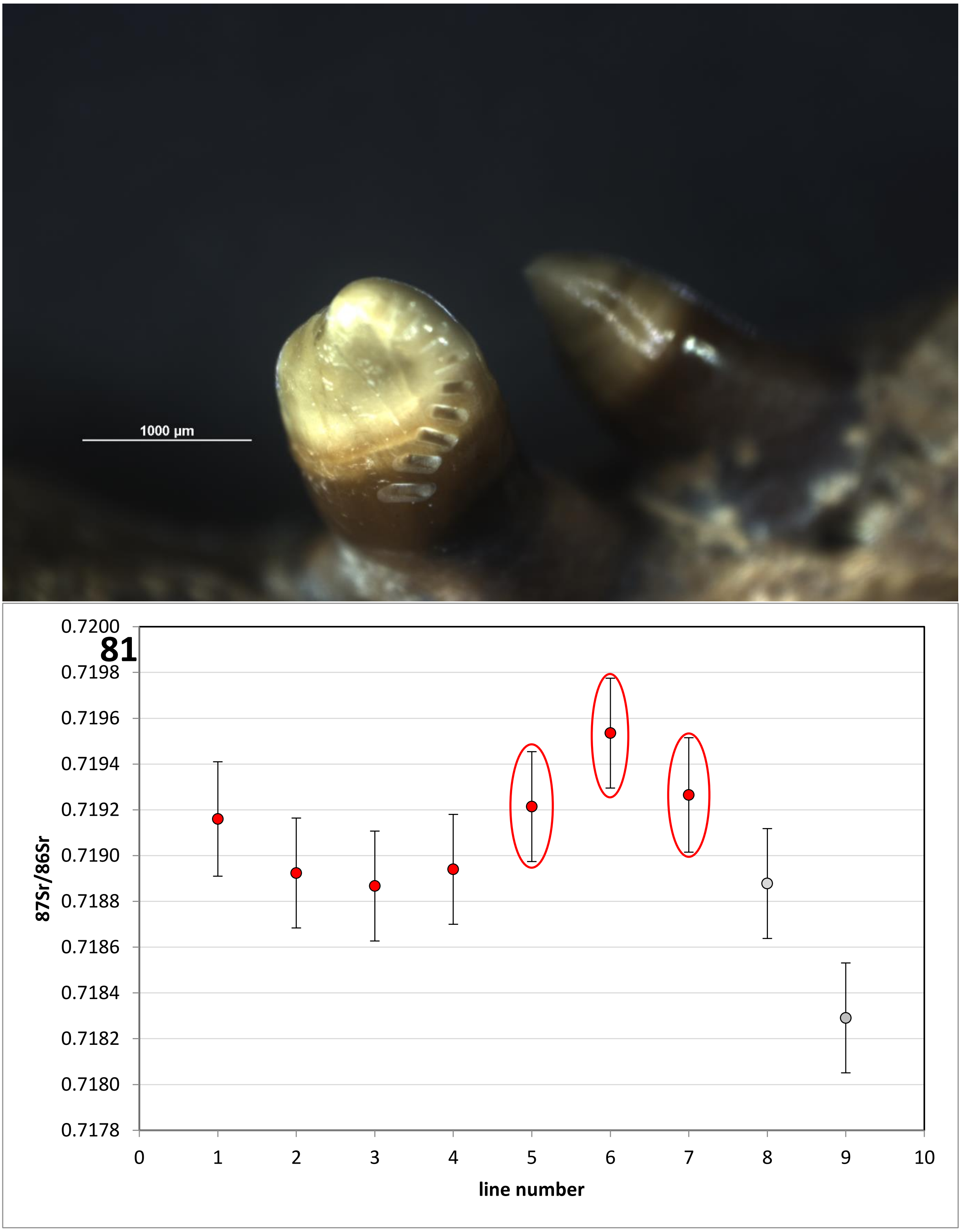

Fig S 7. Close up photograph of ablated cyprinid 81 pharyngeal tooth (upper) and its unique mobility pattern (lower). Red data point indicates 'enamel only' ablation; grey data point indicates enamel-dentine mix and circle around a data point indicate a degree of uncertainty to the assigned category. Photo: Adam Boethius

Cyprinid 82, Roach (Rutilus rutilus), fermentation pit

Table S 15. $^{87}Sr/^{86}Sr$ measurements for Cyprinid 82. Coloured $^{87}Sr/^{86}Sr$ data marks 'enamel only' ablations as inferred from ocular examination of the close-up photograph and/or through changes in $^{87}Rb/^{86}Sr$ ratios. Mean $^{84}Sr/^{86}Sr$ for cyprinid 82 is 0.0564±0.0001 (2SD), with accepted value at 0.0565 (Thirlwall, 1991). [1]Propagated from external reproducibility (2SD) obtained from the primary standard during the analytical session combined with the within-run precision of each analysis (2SE) (Iolite Version 2.5). [2] average of total Sr beam intensity in V (sum of ^{84}Sr, ^{86}Sr, ^{87}Sr, ^{88}Sr). [3] estimated from measured Sr intensity in secondary RM using the same ablation conditions (semi-quantitative).

Line number	Sampling time (sec)	$^{87}Sr/^{86}Sr$	2SE	2SD[1]	$^{87}Rb/^{86}Sr$	2SE	$^{174}Yb^{2+}/^{86}Sr$	2SE	Mean Sr-Beam (V)[2]	Estimated Sr concentration (ppm)[3]	$^{84}Sr/^{86}Sr$	2SE	Data points / line
1	28	0.72029	0.00009	0.00017	0.000418	0.000020	0.000185	0.000025	8.8	590	0.056503	0.000078	57
2	40	0.72061	0.00010	0.00017	0.000499	0.000039	0.000194	0.000026	8.1	540	0.056349	0.000070	80
3	41	0.72061	0.00006	0.00016	0.000351	0.000009	0.000153	0.000024	8.8	590	0.056331	0.000074	81
4	50	0.72073	0.00007	0.00016	0.000243	0.000011	0.000124	0.000021	9.5	640	0.056437	0.000051	101
5	47	0.72044	0.00008	0.00017	0.000959	0.000186	0.000118	0.000022	9.1	610	0.056482	0.000060	94
6	48	0.71930	0.00009	0.00017	0.001609	0.000395	0.000140	0.000016	10.6	720	0.056396	0.000053	96
7	32	0.71874	0.00008	0.00017	0.000938	0.000178	0.000115	0.000019	11.2	750	0.056441	0.000062	64
8	36	0.71786	0.00007	0.00016	0.000854	0.000238	0.000111	0.000016	15.0	1010	0.056388	0.000040	72
9	39	0.71709	0.00009	0.00017	0.000222	0.000020	0.000087	0.000013	16.7	1120	0.056378	0.000041	77
10	43	0.71712	0.00004	0.00015	0.000113	0.000004	0.000068	0.000012	16.8	1130	0.056400	0.000029	87
11	47	0.71730	0.00007	0.00016	0.000110	0.000005	0.000105	0.000013	14.3	960	0.056360	0.000040	95
12	44	0.71737	0.00007	0.00016	0.000120	0.000006	0.000068	0.000011	14.7	990	0.056342	0.000039	89
13	46	0.71737	0.00005	0.00016	0.000132	0.000005	0.000091	0.000011	16.1	1080	0.056318	0.000034	92
14	48	0.71749	0.00009	0.00017	0.000255	0.000012	0.000126	0.000013	14.7	990	0.056318	0.000035	97

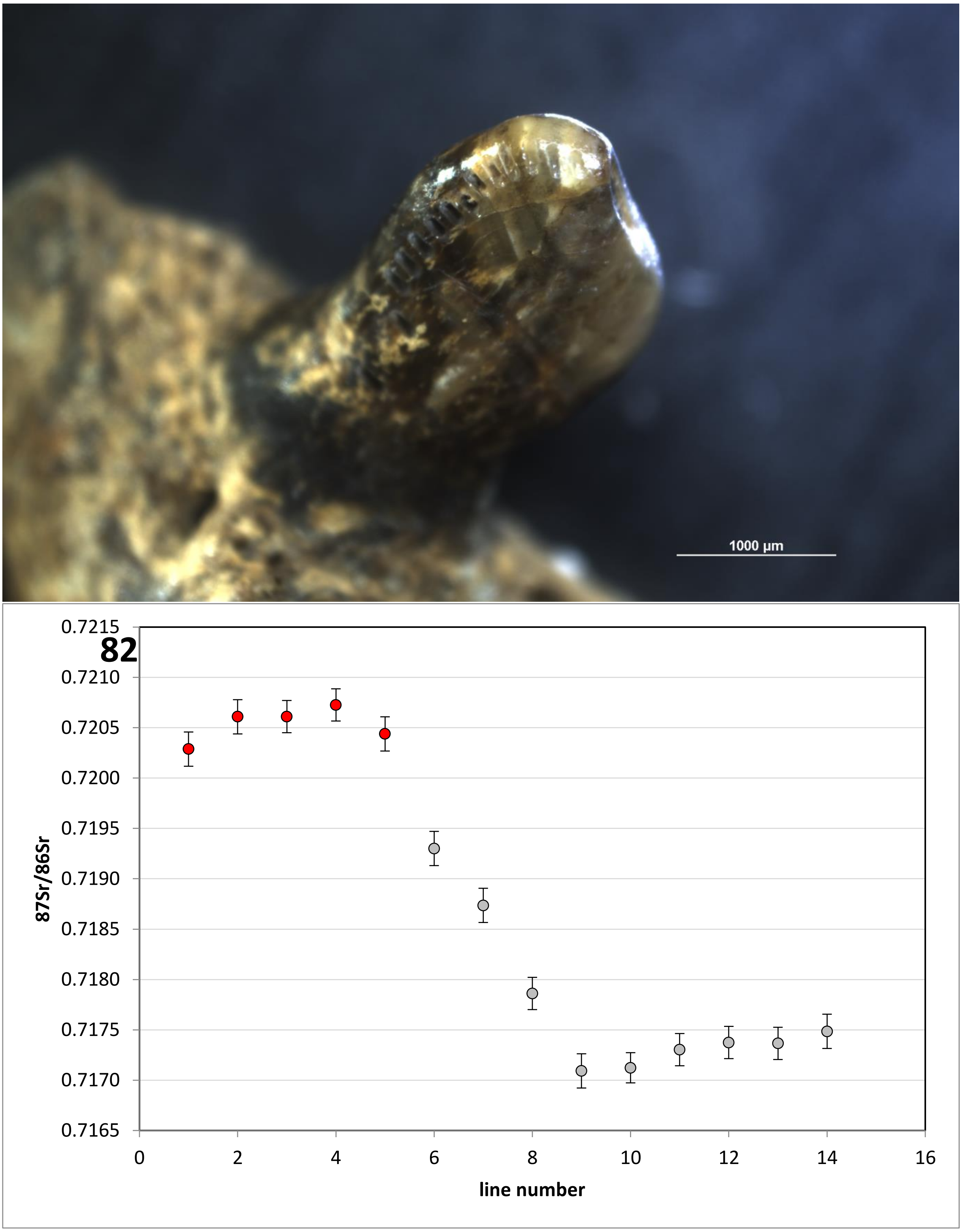

Fig S 8. Close up photograph of ablated cyprinid 82 pharyngeal tooth (upper) and its unique mobility pattern (lower). Red data point indicates ‘enamel only’ ablation; grey data point indicates enamel-dentine mix. Photo: Adam Boethius

Cyprinid 83, Roach (Rutilus rutilus), fermentation pit

Table S 16. $^{87}Sr/^{86}Sr$ measurements for Cyprinid 83. Coloured $^{87}Sr/^{86}Sr$ data marks 'enamel only' ablations as inferred from ocular examination of the close-up photograph and/or through changes in $^{87}Rb/^{86}Sr$ ratios. Mean $^{84}Sr/^{86}Sr$ for cyprinid 83 is 0.0563±0.0003 (2SD), with accepted value at 0.0565 (Thirlwall, 1991). [1]Propagated from external reproducibility (2SD) obtained from the primary standard during the analytical session combined with the within-run precision of each analysis (2SE) (Iolite Version 2.5). [2] average of total Sr beam intensity in V (sum of ^{84}Sr, ^{86}Sr, ^{87}Sr, ^{88}Sr). [3] estimated from measured Sr intensity in secondary RM using the same ablation conditions (semi-quantitative).

Line number	Sampling time (sec)	$^{87}Sr/^{86}Sr$	2SE	2SD[1]	$^{87}Rb/^{86}Sr$	2SE	$^{174}Yb^{2+}/^{86}Sr$	2SE	Mean Sr-Beam (V)[2]	Estimated Sr concentration (ppm)[3]	$^{84}Sr/^{86}Sr$	2SE	Data points / line
1	55	0.71832	0.00005	0.00024	0.000448	0.000011	0.000162	0.000022	9.4	470	0.056412	0.000057	110
2	57	0.71833	0.00003	0.00024	0.000319	0.000006	0.000174	0.000015	11.0	550	0.056464	0.000042	114
3	57	0.71864	0.00005	0.00024	0.000279	0.000008	0.000152	0.000014	12.3	610	0.056433	0.000043	113
4	50	0.71888	0.00004	0.00024	0.000252	0.000005	0.000119	0.000014	15.5	770	0.056373	0.000034	99
5	39	0.71861	0.00005	0.00024	0.000161	0.000011	0.000110	0.000008	22.5	1120	0.056294	0.000026	78
6	39	0.71852	0.00004	0.00024	0.000318	0.000047	0.000116	0.000010	24.4	1210	0.056222	0.000023	77
7	44	0.71844	0.00004	0.00024	0.000337	0.000050	0.000080	0.000007	25.2	1250	0.056160	0.000023	88
8	46	0.71817	0.00004	0.00024	0.000181	0.000013	0.000104	0.000008	25.9	1290	0.056148	0.000026	92
9	38	0.71815	0.00007	0.00025	0.000135	0.000005	0.000103	0.000010	26.3	1310	0.056086	0.000027	75
10	47	0.71826	0.00005	0.00024	0.000118	0.000005	0.000115	0.000007	27.0	1340	0.056157	0.000018	94

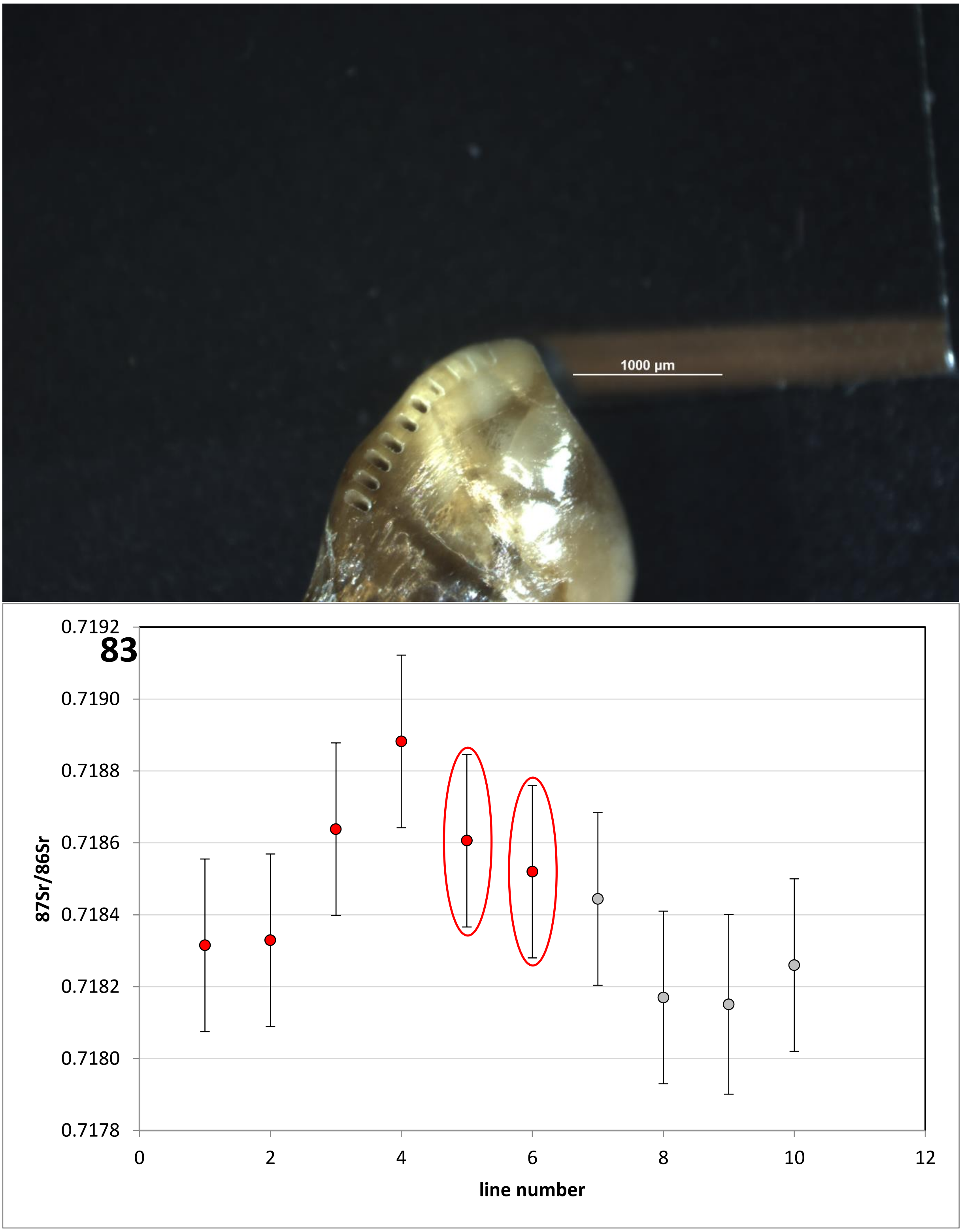

Fig S 9. Close up photograph of ablated cyprinid 83 pharyngeal tooth (upper) and its unique mobility pattern (lower). Red data point indicates 'enamel only' ablation; grey data point indicates enamel-dentine mix and circle around a data point indicate a degree of uncertainty of the assigned category. Photo: Adam Boethius

Cyprinid 84, Roach (Rutilus rutilus), fermentation pit

Table S 17. $^{87}Sr/^{86}Sr$ measurements for Cyprinid 84. Coloured $^{87}Sr/^{86}Sr$ data marks 'enamel only' ablations as inferred from ocular examination of the close-up photograph and/or through changes in $^{87}Rb/^{86}Sr$ ratios. Mean $^{84}Sr/^{86}Sr$ for cyprinid 84 is 0.0564±0.0001 (2SD), with accepted value at 0.0565 (Thirlwall, 1991). [1]Propagated from external reproducibility (2SD) obtained from the primary standard during the analytical session combined with the within-run precision of each analysis (2SE) (Iolite Version 2.5). [2] average of total Sr beam intensity in V (sum of ^{84}Sr, ^{86}Sr, ^{87}Sr, ^{88}Sr). [3] estimated from measured Sr intensity in secondary RM using the same ablation conditions (semi-quantitative).

Line number	Sampling time (sec)	$^{87}Sr/^{86}Sr$	2SE	2SD[1]	$^{87}Rb/^{86}Sr$	2SE	$^{174}Yb^{2+}/^{86}Sr$	2SE	Mean Sr-Beam (V)[2]	Estimated Sr concentration (ppm)[3]	$^{84}Sr/^{86}Sr$	2SE	Data points / line
1	34	0.71902	0.00010	0.00026	0.000470	0.000021	0.000107	0.000018	12.8	640	0.056475	0.000053	67
2	45	0.71907	0.00007	0.00025	0.000435	0.000018	0.000119	0.000017	13.2	650	0.056411	0.000044	89
3	47	0.71912	0.00007	0.00025	0.000288	0.000014	0.000138	0.000017	12.9	640	0.056386	0.000037	94
4	46	0.71912	0.00005	0.00024	0.000205	0.000006	0.000139	0.000014	14.6	720	0.056427	0.000042	92
5	41	0.71827	0.00006	0.00024	0.000158	0.000005	0.000082	0.000012	17.9	890	0.056425	0.000037	83
6	34	0.71719	0.00007	0.00025	0.000399	0.000079	0.000081	0.000012	21.3	1060	0.056387	0.000027	68
7	40	0.71602	0.00007	0.00025	0.000404	0.000082	0.000095	0.000009	23.1	1150	0.056330	0.000030	81
8	42	0.71573	0.00004	0.00024	0.000196	0.000020	0.000096	0.000010	22.8	1130	0.056255	0.000027	85
9	34	0.71583	0.00005	0.00024	0.000162	0.000009	0.000077	0.000010	22.0	1100	0.056269	0.000028	68

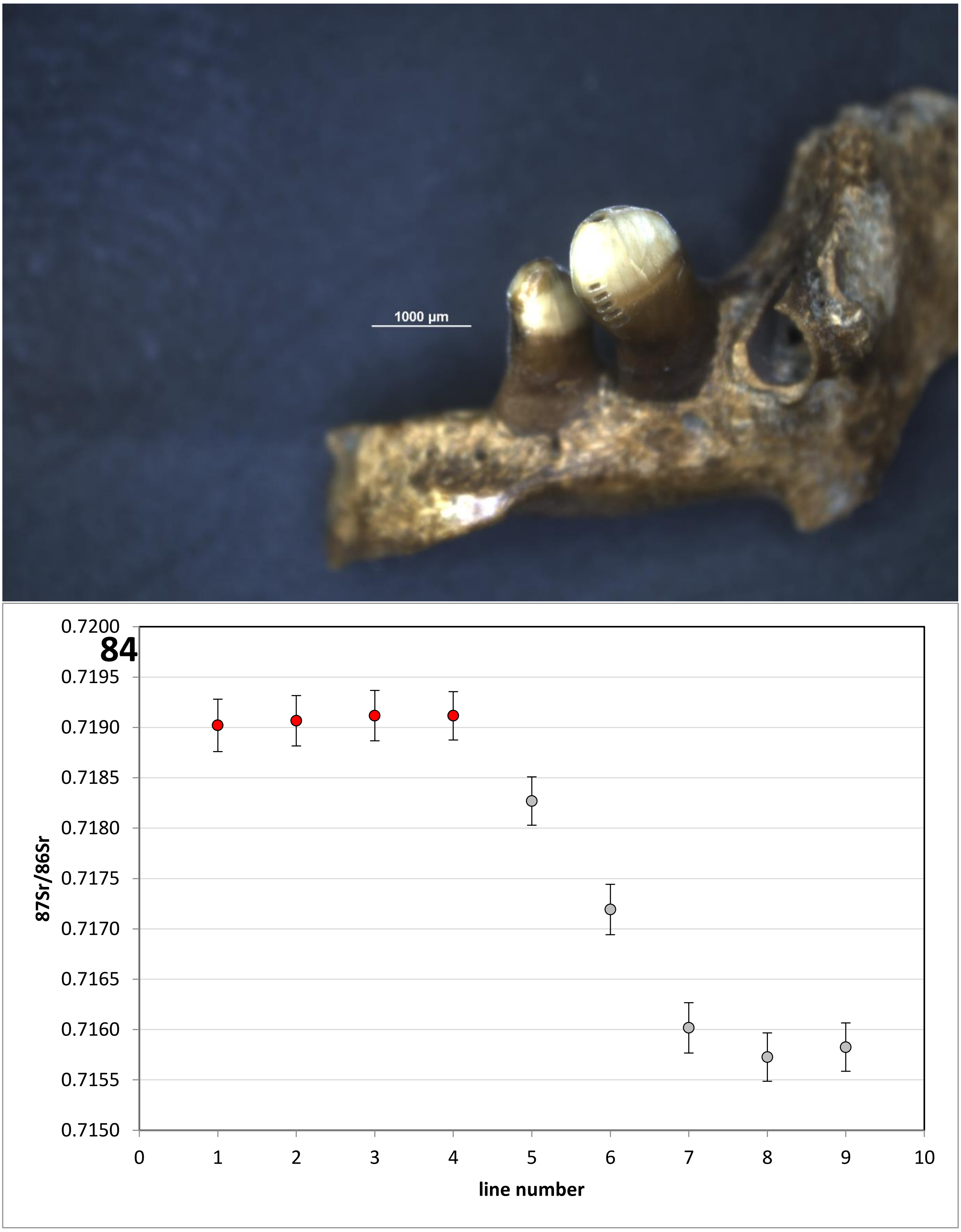

Fig S 10. Close up photograph of ablated cyprinid 84 pharyngeal tooth (upper) and its unique mobility pattern (lower). Red data point indicates ‘enamel only’ ablation; grey data point indicates enamel-dentine mix. Photo: Adam Boethius

Cyprinid 90, Roach (Rutilus rutilus), other areas

Table S 18. $^{87}Sr/^{86}Sr$ measurements for Cyprinid 90. Coloured $^{87}Sr/^{86}Sr$ data marks 'enamel only' ablations as inferred from ocular examination of the close-up photograph and/or through changes in $^{87}Rb/^{86}Sr$ ratios. Mean $^{84}Sr/^{86}Sr$ for cyprinid 90 is 0.0562±0.0002 (2SD), with accepted value at 0.0565 (Thirlwall, 1991). [1]Propagated from external reproducibility (2SD) obtained from the primary standard during the analytical session combined with the within-run precision of each analysis (2SE) (Iolite Version 2.5). [2] average of total Sr beam intensity in V (sum of ^{84}Sr, ^{86}Sr, ^{87}Sr, ^{88}Sr). [3] estimated from measured Sr intensity in secondary RM using the same ablation conditions (semi-quantitative).

Line number	Sampling time (sec)	$^{87}Sr/^{86}Sr$	2SE	2SD[1]	$^{87}Rb/^{86}Sr$	2SE	$^{174}Yb^{2+}/^{86}Sr$	2SE	Mean Sr-Beam (V)[2]	Estimated Sr concentration (ppm)[3]	$^{84}Sr/^{86}Sr$	2SE	Data points / line
1	30	0.71613	0.00007	0.00025	0.000585	0.000012	0.000139	0.000019	14.6	730	0.056151	0.000043	59
2	44	0.71656	0.00005	0.00024	0.000541	0.000009	0.000153	0.000014	13.7	680	0.056213	0.000040	88
3	48	0.71668	0.00005	0.00024	0.000555	0.000015	0.000177	0.000015	14.3	710	0.056309	0.000042	95
4	31	0.71631	0.00006	0.00024	0.000487	0.000018	0.000123	0.000012	16.6	830	0.056225	0.000036	63
5	37	0.71533	0.00006	0.00024	0.000339	0.000016	0.000103	0.000012	20.8	1030	0.056184	0.000025	73
6	46	0.71600	0.00005	0.00024	0.000376	0.000015	0.000122	0.000014	16.0	800	0.056214	0.000034	91
7	50	0.71497	0.00004	0.00024	0.000310	0.000014	0.000048	0.000010	19.1	950	0.056052	0.000026	100

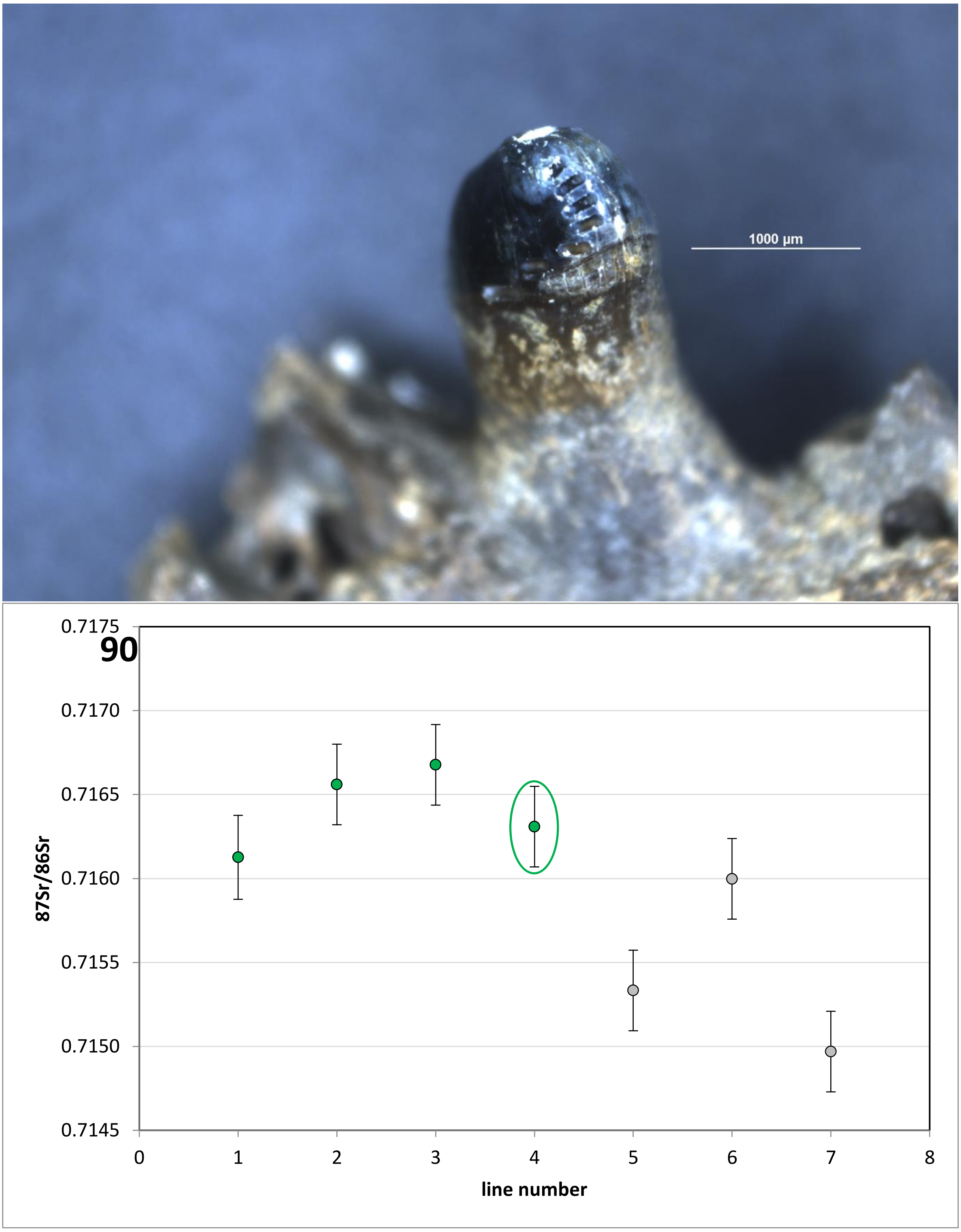

Fig S 11. Close up photograph of ablated cyprinid 90 pharyngeal tooth (upper) and its unique mobility pattern (lower). Greed data point indicates 'enamel only' ablation; grey data point indicates enamel-dentine mix and circle around a data point indicate a degree of uncertainty to the assigned category. Photo: Adam Boethius

Cyprinid 91, Roach (Rutilus rutilus), other areas

Table S 19. $^{87}Sr/^{86}Sr$ measurements for Cyprinid 91. Coloured $^{87}Sr/^{86}Sr$ data marks 'enamel only' ablations as inferred from ocular examination of the close-up photograph and/or through changes in $^{87}Rb/^{86}Sr$ ratios. Mean $^{84}Sr/^{86}Sr$ for cyprinid 91 is 0.0565±0.0001 (2SD), with accepted value at 0.0565 (Thirlwall, 1991). [1]Propagated from external reproducibility (2SD) obtained from the primary standard during the analytical session combined with the within-run precision of each analysis (2SE) (Iolite Version 2.5). [2] average of total Sr beam intensity in V (sum of ^{84}Sr, ^{86}Sr, ^{87}Sr, ^{88}Sr). [3] estimated from measured Sr intensity in secondary RM using the same ablation conditions (semi-quantitative).

Line number	Sampling time (sec)	$^{87}Sr/^{86}Sr$	2SE	2SD[1]	$^{87}Rb/^{86}Sr$	2SE	$^{174}Yb^{2+}/^{86}Sr$	2SE	Mean Sr-Beam (V)[2]	Estimated Sr concentration (ppm)[3]	$^{84}Sr/^{86}Sr$	2SE	Data points / line
1	33	0.71756	0.00016	0.00021	0.000516	0.000015	0.000207	0.000030	7.8	520	0.056448	0.000088	66
2	43	0.71711	0.00015	0.00021	0.000481	0.000015	0.000189	0.000024	8.4	560	0.056539	0.000067	86
3	42	0.71684	0.00013	0.00020	0.000498	0.000011	0.000189	0.000021	8.3	560	0.056405	0.000072	84
4	44	0.71735	0.00011	0.00018	0.000554	0.000014	0.000119	0.000024	7.5	500	0.056468	0.000072	88
5	39	0.71745	0.00007	0.00016	0.000490	0.000012	0.000226	0.000027	7.1	480	0.056509	0.000077	78
6	38	0.71604	0.00008	0.00016	0.000278	0.000009	0.000149	0.000021	9.6	640	0.056433	0.000051	77
7	41	0.71478	0.00008	0.00017	0.000293	0.000018	0.000147	0.000016	12.3	830	0.056350	0.000046	81

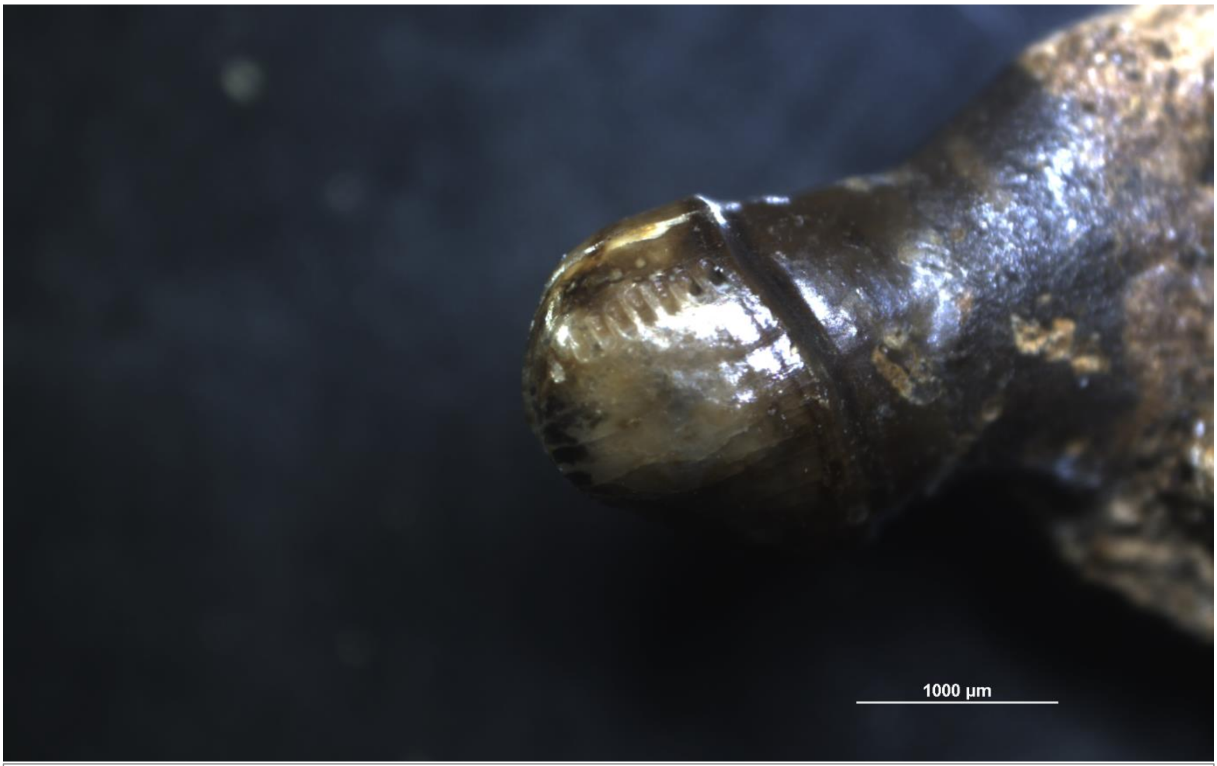

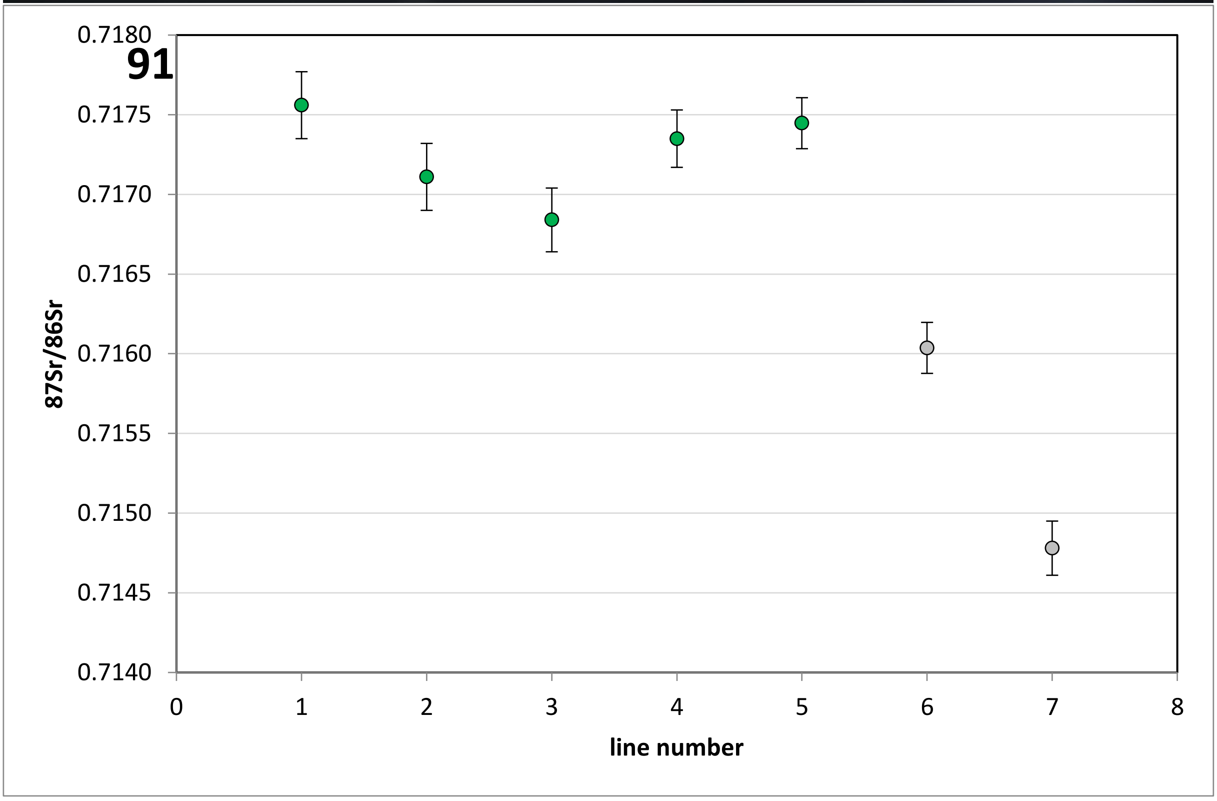

Fig S 12. Close up photograph of ablated cyprinid 91 pharyngeal tooth (upper) and unique mobility pattern (lower). Green data point indicates ‘enamel only’ ablation; grey data point indicates enamel-dentine mix. Photo: Adam Boethius

Cyprinid 95, Cyprinidae indet., other areas

Table S 20. $^{87}Sr/^{86}Sr$ measurements for Cyprinid 95. Coloured $^{87}Sr/^{86}Sr$ data marks 'enamel only' ablations as inferred from ocular examination of the close-up photograph and/or through changes in $^{87}Rb/^{86}Sr$ ratios. Mean $^{84}Sr/^{86}Sr$ for cyprinid 95 is 0.0564±0.0001 (2SD), with accepted value at 0.0565 (Thirlwall. 1991). [1]Propagated from external reproducibility (2SD) obtained from the primary standard during the analytical session combined with the within-run precision of each analysis (2SE) (Iolite Version 2.5). [2] average of total Sr beam intensity in V (sum of ^{84}Sr, ^{86}Sr, ^{87}Sr, ^{88}Sr). [3] estimated from measured Sr intensity in secondary RM using the same ablation conditions (semi-quantitative).

Line number	Sampling time (sec)	$^{87}Sr/^{86}Sr$	2SE	2SD[1]	$^{87}Rb/^{86}Sr$	2SE	$^{174}Yb^{2+}/^{86}Sr$	2SE	Mean Sr-Beam (V)[2]	Estimated Sr concentration (ppm)[3]	$^{84}Sr/^{86}Sr$	2SE	Data points / line
1	42	0.71759	0.00005	0.00024	0.000692	0.000015	0.000316	0.000022	9.1	450	0.056395	0.000057	85
2	49	0.71725	0.00005	0.00024	0.000693	0.000014	0.000452	0.000025	8.4	420	0.056460	0.000057	97
3	48	0.71733	0.00005	0.00024	0.000799	0.000048	0.000354	0.000023	9.0	450	0.056352	0.000061	96
4	42	0.71738	0.00005	0.00024	0.000576	0.000011	0.000373	0.000025	9.2	460	0.056376	0.000059	85
5	30	0.71705	0.00009	0.00025	0.000540	0.000013	0.000491	0.000047	9.6	480	0.056363	0.000060	60
6	42	0.71715	0.00007	0.00025	0.000579	0.000011	0.000329	0.000025	9.2	460	0.056395	0.000054	83
7	43	0.71738	0.00006	0.00024	0.000709	0.000019	0.000324	0.000022	9.2	460	0.056352	0.000064	86
8	45	0.71695	0.00005	0.00024	0.000888	0.000011	0.000259	0.000019	10.2	510	0.056397	0.000047	90
9	45	0.71667	0.00009	0.00025	0.000815	0.000018	0.000617	0.000049	10.1	500	0.056394	0.000049	90
10	47	0.71726	0.00007	0.00025	0.000660	0.000017	0.000278	0.000023	9.7	480	0.056463	0.000055	94
11	43	0.71633	0.00007	0.00025	0.000688	0.000035	0.000259	0.000022	12.8	640	0.056340	0.000044	86
12	41	0.71441	0.00007	0.00024	0.000546	0.000056	0.000509	0.000043	20.9	1040	0.056419	0.000028	81
13	46	0.71391	0.00006	0.00024	0.000342	0.000010	0.000823	0.000034	25.2	1250	0.056463	0.000026	92

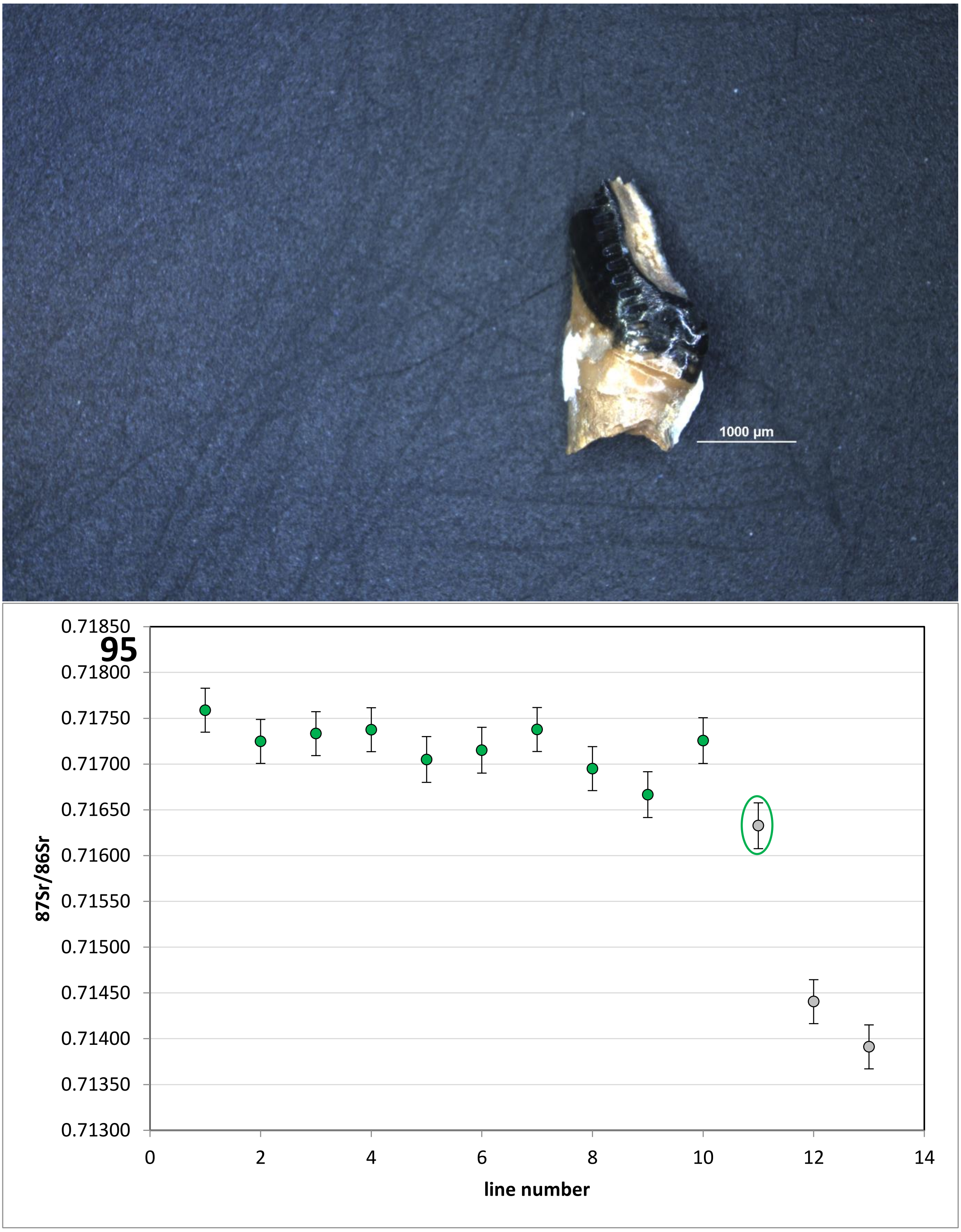

Fig S 13. Close up photograph of ablated cyprinid 95 pharyngeal tooth (upper) and unique mobility pattern (lower). Green data point indicates ‘enamel only’ ablation; grey data point indicates enamel-dentine mix and circle around a data point indicate a degree of uncertainty to the assigned category. Photo: Adam Boethius

Cyprinid 119, Roach (Rutilus rutilus), other areas

Table S 21. $^{87}Sr/^{86}Sr$ measurements for Cyprinid 119. Coloured $^{87}Sr/^{86}Sr$ data marks 'enamel only' ablations as inferred from ocular examination of the close-up photograph and/or through changes in $^{87}Rb/^{86}Sr$ ratios. Mean $^{84}Sr/^{86}Sr$ for cyprinid 119 is 0.0563±0.0001 (2SD), with accepted value at 0.0565 (Thirlwall, 1991). [1]Propagated from external reproducibility (2SD) obtained from the primary standard during the analytical session combined with the within-run precision of each analysis (2SE) (Iolite Version 2.5). [2] average of total Sr beam intensity in V (sum of ^{84}Sr, ^{86}Sr, ^{87}Sr, ^{88}Sr). [3] estimated from measured Sr intensity in secondary RM using the same ablation conditions (semi-quantitative).

Line number	Sampling time (sec)	$^{87}Sr/^{86}Sr$	2SE	2SD[1]	$^{87}Rb/^{86}Sr$	2SE	$^{174}Yb^{2+}/^{86}Sr$	2SE	Mean Sr-Beam (V)[2]	Estimated Sr concentration (ppm)[3]	$^{84}Sr/^{86}Sr$	2SE	Data points / line
1	44	0.71568	0.00017	0.00026	0.000451	0.000013	0.000110	0.000018	11.0	580	0.056219	0.000052	87
2	37	0.71507	0.00021	0.00029	0.000390	0.000017	0.000128	0.000022	12.2	640	0.056347	0.000044	74
3	40	0.71511	0.00017	0.00026	0.000454	0.000011	0.000126	0.000015	13.3	700	0.056323	0.000036	79
4	36	0.71511	0.00022	0.00029	0.000394	0.000032	0.000138	0.000023	13.0	680	0.056351	0.000056	72
5	33	0.71459	0.00010	0.00022	0.000915	0.000131	0.000131	0.000018	15.2	800	0.056319	0.000031	66

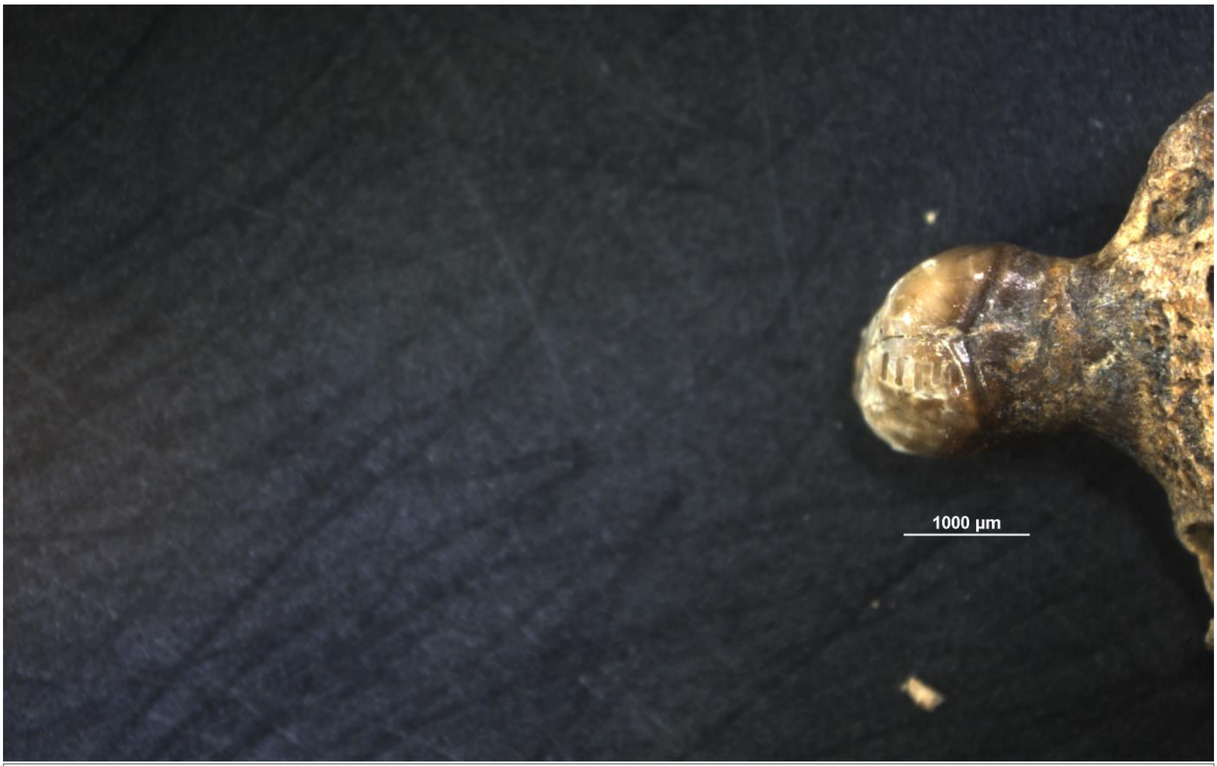

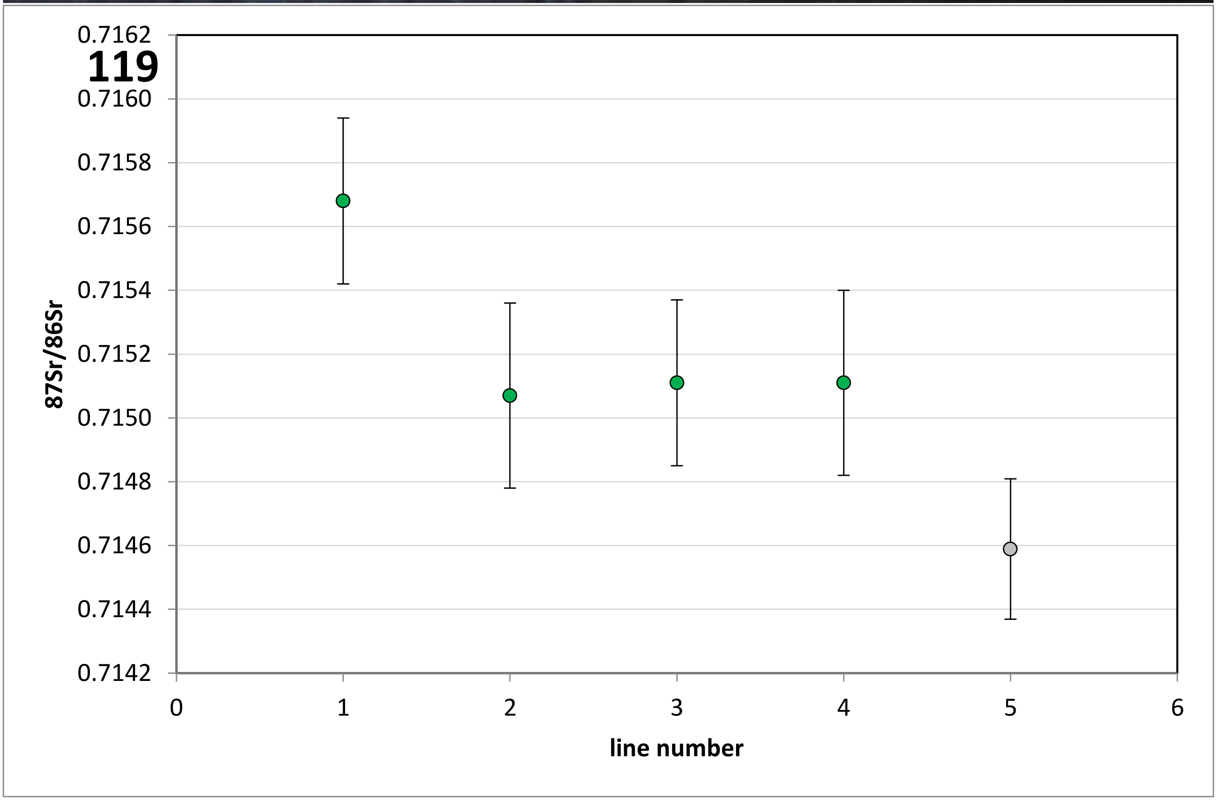

Fig S 14. Close up photograph of ablated cyprinid 119 pharyngeal tooth (upper) and unique mobility pattern (lower). Green data point indicates 'enamel only' ablation; grey data point indicates enamel-dentine mix. Photo: Adam Boethius

Cyprinid 120, Roach (Rutilus rutilus), other areas

Table S 22. $^{87}Sr/^{86}Sr$ measurements for Cyprinid 120. Coloured $^{87}Sr/^{86}Sr$ data marks 'enamel only' ablations as inferred from ocular examination of the close-up photograph and/or through changes in $^{87}Rb/^{86}Sr$ ratios. Mean $^{84}Sr/^{86}Sr$ for cyprinid 120 is 0.0564±0.0002 (2SD), with accepted value at 0.0565 (Thirlwall, 1991). [1]Propagated from external reproducibility (2SD) obtained from the primary standard during the analytical session combined with the within-run precision of each analysis (2SE) (Iolite Version 2.5). [2] average of total Sr beam intensity in V (sum of ^{84}Sr, ^{86}Sr, ^{87}Sr, ^{88}Sr). [3] estimated from measured Sr intensity in secondary RM using the same ablation conditions (semi-quantitative).

Line number	Sampling time (sec)	$^{87}Sr/^{86}Sr$	2SE	2SD[1]	$^{87}Rb/^{86}Sr$	2SE	$^{174}Yb^{2+}/^{86}Sr$	2SE	Mean Sr-Beam (V)[2]	Estimated Sr concentration (ppm)[3]	$^{84}Sr/^{86}Sr$	2SE	Data points / line
1	41	0.71543	0.00012	0.00024	0.000789	0.000031	0.000155	0.000024	10.1	530	0.056364	0.000054	82
2	48	0.71551	0.00010	0.00022	0.000658	0.000033	0.000187	0.000020	10.6	550	0.056407	0.000048	96
3	47	0.71546	0.00009	0.00022	0.000632	0.000031	0.000145	0.000016	11.5	600	0.056446	0.000044	94
4	45	0.71500	0.00007	0.00021	0.000552	0.000048	0.000145	0.000016	11.1	580	0.056394	0.000048	89
5	40	0.71457	0.00007	0.00021	0.000462	0.000034	0.000149	0.000019	12.0	630	0.056426	0.000044	80
6	38	0.71396	0.00009	0.00022	0.000560	0.000049	0.000135	0.000018	13.2	690	0.056314	0.000052	75
7	37	0.71382	0.00007	0.00021	0.000628	0.000066	0.000106	0.000013	14.2	750	0.056247	0.000042	74
8	38	0.71399	0.00006	0.00021	0.000498	0.000044	0.000117	0.000012	17.2	900	0.056203	0.000028	75

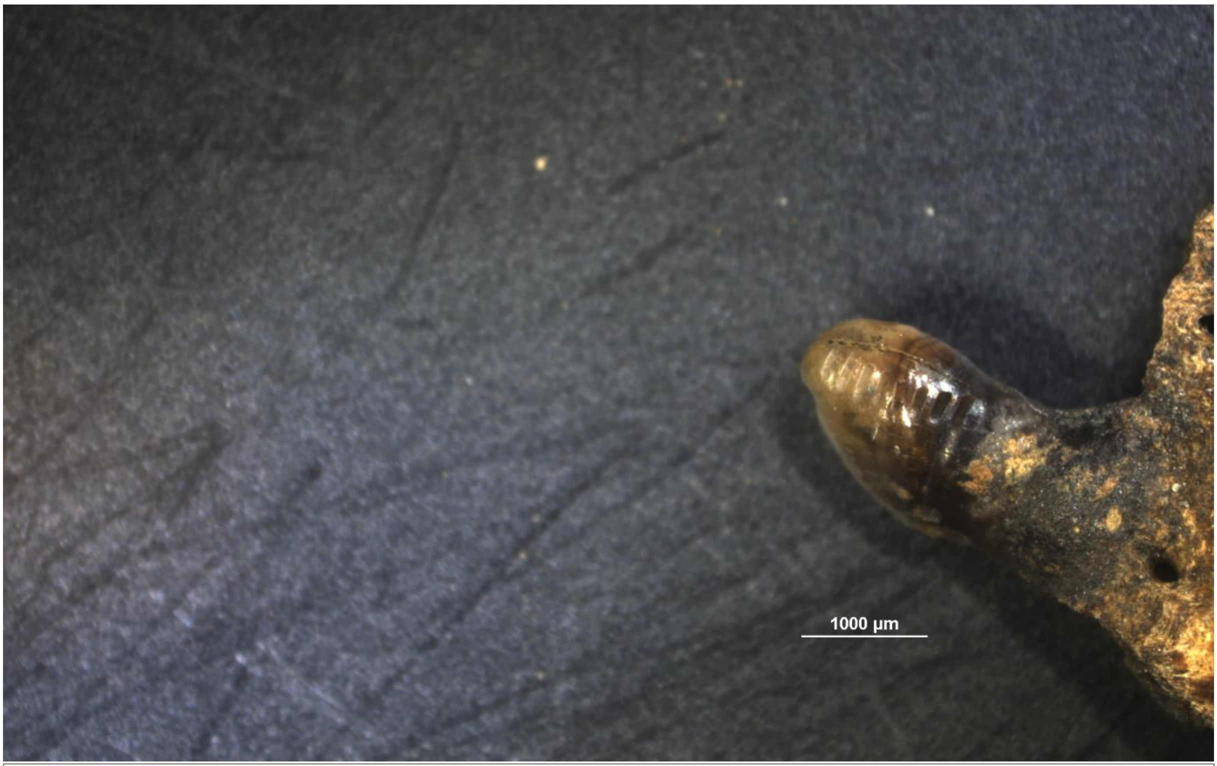

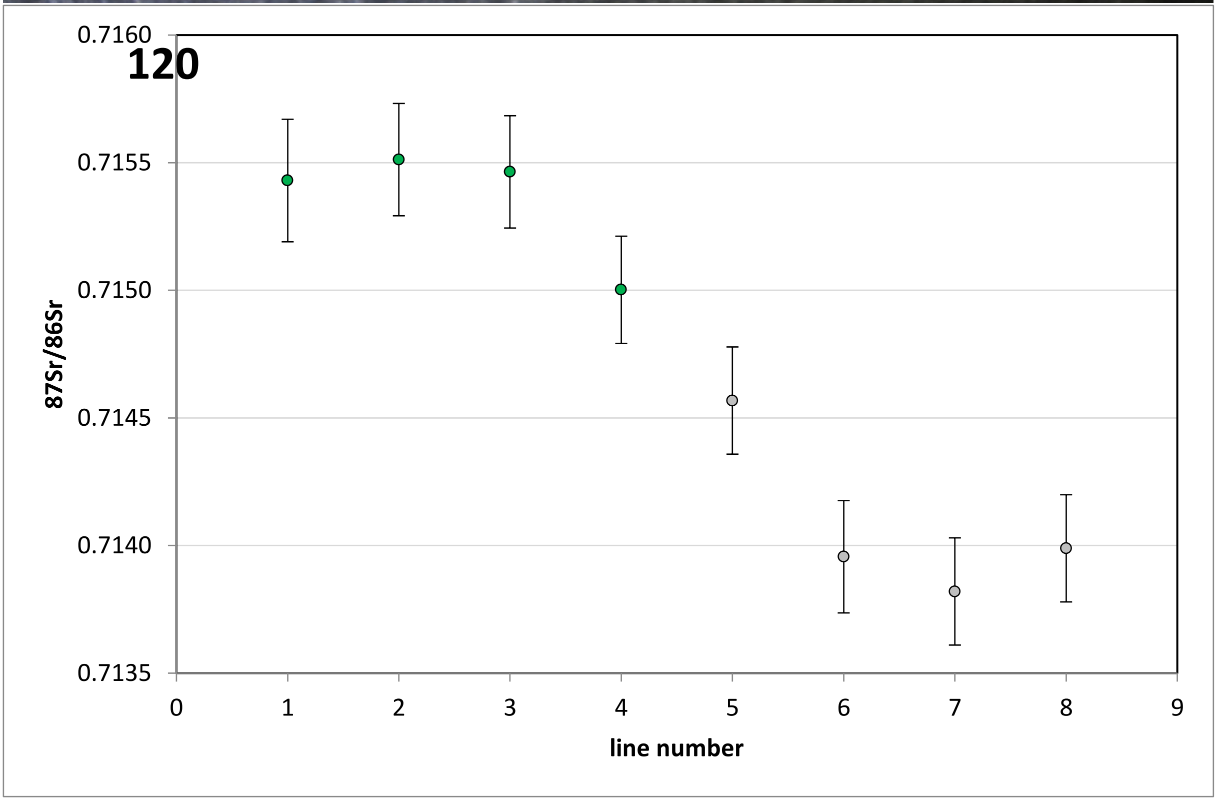

Fig S 15. Close up photograph of ablated cyprinid 120 pharyngeal tooth (upper) and unique mobility pattern (lower). Green data point indicates ‘enamel only’ ablation; grey data point indicates enamel-dentine mix. Photo: Adam Boethius

Cyprinid 121, Roach (Rutilus rutilus), other areas

Table S 23. $^{87}Sr/^{86}Sr$ measurements for Cyprinid 121. Coloured $^{87}Sr/^{86}Sr$ data marks 'enamel only' ablations as inferred from ocular examination of the close-up photograph and/or through changes in $^{87}Rb/^{86}Sr$ ratios. Mean $^{84}Sr/^{86}Sr$ for cyprinid 121 is 0.0561±0.0004 (2SD), with accepted value at 0.0565 (Thirlwall, 1991). [1]Propagated from external reproducibility (2SD) obtained from the primary standard during the analytical session combined with the within-run precision of each analysis (2SE) (Iolite Version 2.5). [2] average of total Sr beam intensity in V (sum of ^{84}Sr, ^{86}Sr, ^{87}Sr, ^{88}Sr). [3] estimated from measured Sr intensity in secondary RM using the same ablation conditions (semi-quantitative).

Line number	Sampling time (sec)	$^{87}Sr/^{86}Sr$	2SE	2SD[1]	$^{87}Rb/^{86}Sr$	2SE	$^{174}Yb^{2+}/^{86}Sr$	2SE	Mean Sr-Beam (V)[2]	Estimated Sr concentration (ppm)[3]	$^{84}Sr/^{86}Sr$	2SE	Data points / line
1	22	0.71533	0.00014	0.00024	0.001143	0.000085	0.000843	0.000039	12.1	630	0.055601	0.000070	44
2	34	0.71569	0.00016	0.00025	0.000832	0.000051	0.000714	0.000082	11.9	620	0.055922	0.000064	68
3	38	0.71531	0.00010	0.00022	0.000850	0.000049	0.000814	0.000038	13.5	710	0.056013	0.000046	77
4	41	0.71597	0.00010	0.00022	0.000615	0.000027	0.000599	0.000030	11.8	620	0.055989	0.000048	81
5	38	0.71653	0.00008	0.00022	0.000566	0.000037	0.000513	0.000035	11.1	580	0.056252	0.000059	75
6	36	0.71634	0.00008	0.00022	0.000759	0.000049	0.000530	0.000037	10.6	560	0.056232	0.000057	73
7	37	0.71640	0.00010	0.00022	0.000546	0.000036	0.000482	0.000048	11.0	580	0.056193	0.000056	74
8	43	0.71665	0.00007	0.00021	0.000549	0.000046	0.000344	0.000025	10.5	550	0.056304	0.000051	85
9	45	0.71480	0.00010	0.00022	0.000328	0.000027	0.000633	0.000079	16.1	840	0.056063	0.000049	91

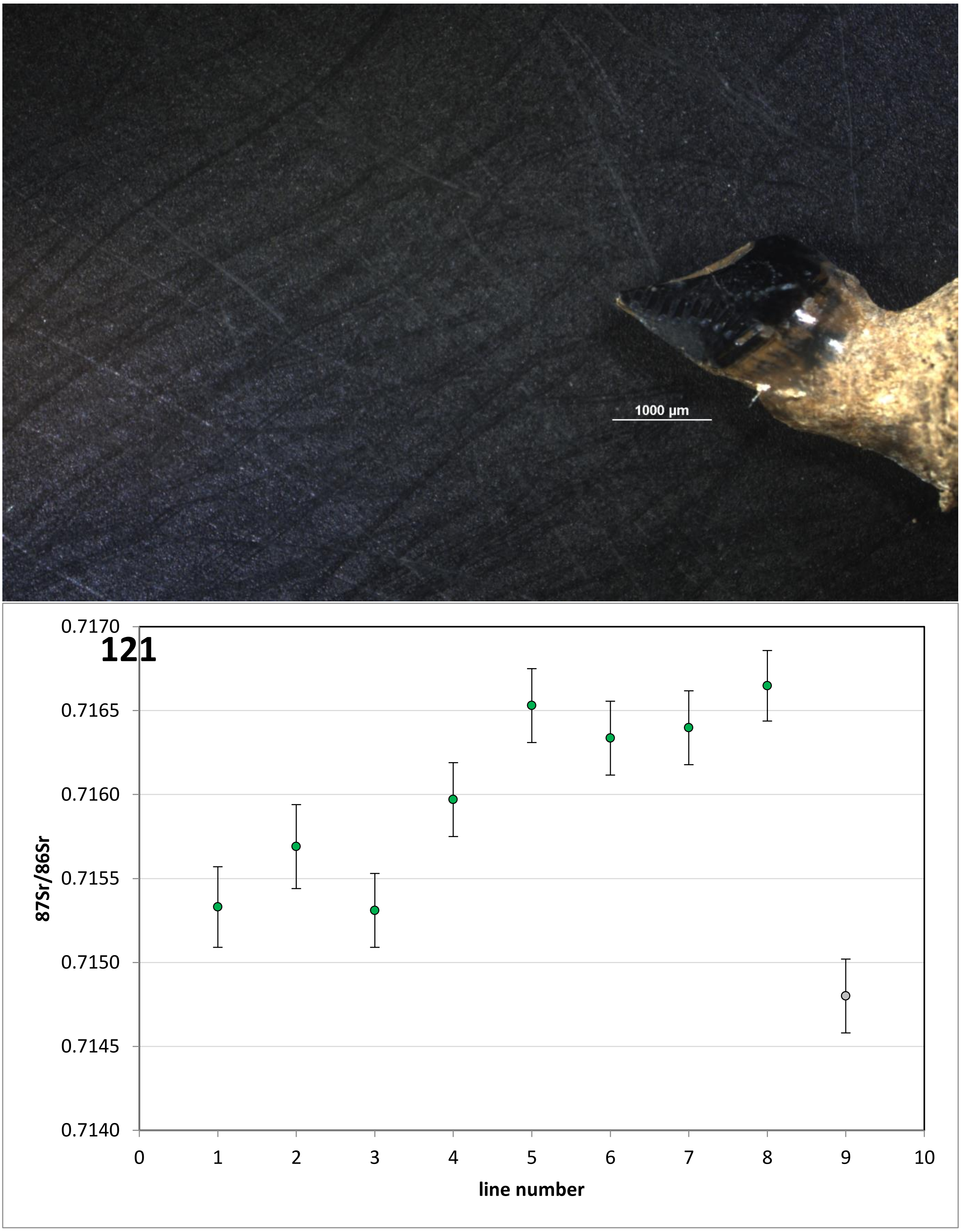

Fig S 16. Close up photograph of ablated cyprinid 121 pharyngeal tooth (upper) and unique mobility pattern (lower). Green data point indicates ‘enamel only’ ablation; grey data point indicates enamel-dentine mix. Photo: Adam Boethius

Cyprinid 122, Cyprinidae indet., other areas

Table S 24. $^{87}Sr/^{86}Sr$ measurements for Cyprinid 122. Coloured $^{87}Sr/^{86}Sr$ data marks 'enamel only' ablations as inferred from ocular examination of the close-up photograph and/or through changes in $^{87}Rb/^{86}Sr$ ratios. Mean $^{84}Sr/^{86}Sr$ for cyprinid 122 is 0.0562±0.0002 (2SD), with accepted value at 0.0565 (Thirlwall, 1991). [1]Propagated from external reproducibility (2SD) obtained from the primary standard during the analytical session combined with the within-run precision of each analysis (2SE) (Iolite Version 2.5). [2] average of total Sr beam intensity in V (sum of ^{84}Sr, ^{86}Sr, ^{87}Sr, ^{88}Sr). [3] estimated from measured Sr intensity in secondary RM using the same ablation conditions (semi-quantitative).

Line number	Sampling time (sec)	$^{87}Sr/^{86}Sr$	2SE	2SD[1]	$^{87}Rb/^{86}Sr$	2SE	$^{174}Yb^{2+}/^{86}Sr$	2SE	Mean Sr-Beam (V)[2]	Estimated Sr concentration (ppm)[3]	$^{84}Sr/^{86}Sr$	2SE	Data points / line
1	28	0.71535	0.00012	0.00023	0.000486	0.000022	0.000515	0.000035	11.3	590	0.056123	0.000064	56
2	40	0.71494	0.00008	0.00021	0.000384	0.000016	0.000500	0.000023	13.3	700	0.056243	0.000045	80
3	41	0.71504	0.00007	0.00021	0.000510	0.000018	0.000506	0.000020	12.9	680	0.056245	0.000051	83
4	45	0.71504	0.00008	0.00021	0.000928	0.000043	0.000370	0.000019	13.4	700	0.056173	0.000039	90
5	43	0.71516	0.00007	0.00021	0.000453	0.000023	0.000425	0.000023	13.6	710	0.056126	0.000052	87
6	43	0.71513	0.00012	0.00023	0.000451	0.000026	0.000541	0.000054	12.7	670	0.056151	0.000052	86
7	42	0.71542	0.00005	0.00021	0.000440	0.000012	0.000352	0.000017	13.2	690	0.056229	0.000046	85
8	43	0.71525	0.00008	0.00021	0.000425	0.000021	0.000386	0.000023	13.7	720	0.056346	0.000042	87
9	39	0.71539	0.00005	0.00021	0.000444	0.000026	0.000333	0.000023	12.4	650	0.056255	0.000044	78
10	44	0.71551	0.00006	0.00021	0.000566	0.000012	0.000320	0.000018	12.5	660	0.056277	0.000042	88
11	45	0.71555	0.00008	0.00021	0.000890	0.000032	0.000342	0.000018	12.6	660	0.056197	0.000045	91
12	50	0.71536	0.00007	0.00021	0.000804	0.000027	0.000286	0.000016	14.2	740	0.056252	0.000041	100
13	51	0.71533	0.00006	0.00021	0.000683	0.000041	0.000333	0.000019	14.6	760	0.056048	0.000068	103
14	48	0.71537	0.00006	0.00021	0.000542	0.000016	0.000384	0.000022	15.4	810	0.056219	0.000041	96
15	46	0.71522	0.00006	0.00021	0.000843	0.000038	0.000439	0.000021	14.6	760	0.056069	0.000046	91

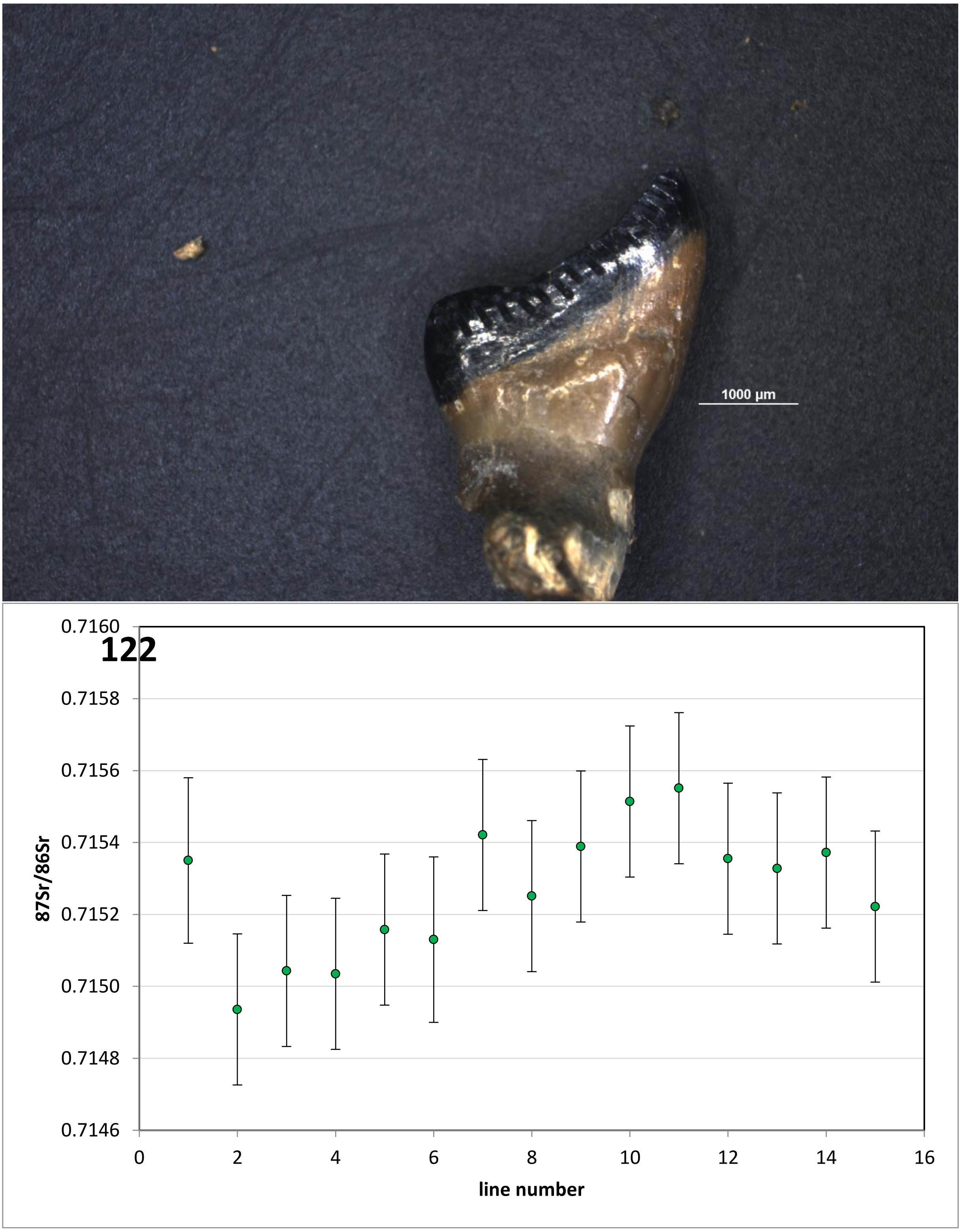

Fig S 17. Close up photograph of ablated cyprinid 122 pharyngeal tooth (upper) and unique mobility pattern (lower). Green data points indicate 'enamel only' ablations. Photo: Adam Boethius

Pikes (Esox lucius)

Pike 85 (Esox lucius), fermentation pit

Table S 25. $^{87}Sr/^{86}Sr$ measurements for Pike 85. Coloured $^{87}Sr/^{86}Sr$ data marks 'enamel only' ablations as inferred from ocular examination of the close-up photograph and/or through changes in $^{87}Rb/^{86}Sr$ ratios. Mean $^{84}Sr/^{86}Sr$ for Pike 85 is 0.0561±0.0002 (2SD), with accepted value at 0.0565 (Thirlwall, 1991). [1]Propagated from external reproducibility (2SD) obtained from the primary standard during the analytical session combined with the within-run precision of each analysis (2SE) (Iolite Version 2.5). [2] average of total Sr beam intensity in V (sum of ^{84}Sr, ^{86}Sr, ^{87}Sr, ^{88}Sr). [3] estimated from measured Sr intensity in secondary RM using the same ablation conditions (semi-quantitative).

Line number	Sampling time (sec)	$^{87}Sr/^{86}Sr$	2SE	2SD[1]	$^{87}Rb/^{86}Sr$	2SE	$^{174}Yb^{2+}/^{86}Sr$	2SE	Mean Sr-Beam (V)[2]	Estimated Sr concentration (ppm)[3]	$^{84}Sr/^{86}Sr$	2SE	Data points / line
1	58	0.71674	0.00004	0.00020	0.000410	0.000013	0.000121	0.000011	17.6	920	0.056163	0.000027	115
2	65	0.71679	0.00005	0.00021	0.000538	0.000029	0.000110	0.000010	19.7	1030	0.056196	0.000026	129
3	66	0.71689	0.00006	0.00021	0.000663	0.000046	0.000108	0.000011	18.0	950	0.056159	0.000026	132
4	67	0.71693	0.00006	0.00021	0.001020	0.000119	0.000118	0.000010	17.7	930	0.056213	0.000026	133
5	67	0.71674	0.00007	0.00021	0.000816	0.000048	0.000104	0.000010	18.7	980	0.056156	0.000023	134
6	54	0.71672	0.00008	0.00022	0.000695	0.000039	0.000115	0.000011	18.3	960	0.056243	0.000026	108
7	63	0.71662	0.00006	0.00021	0.001412	0.000089	0.000101	0.000010	17.8	930	0.056178	0.000029	125
8	45	0.71664	0.00006	0.00021	0.000797	0.000049	0.000104	0.000011	19.5	1020	0.056112	0.000029	91
9	62	0.71672	0.00007	0.00021	0.002156	0.000198	0.000106	0.000009	18.2	950	0.056052	0.000024	124
10	69	0.71689	0.00006	0.00021	0.001543	0.000088	0.000112	0.000010	18.0	940	0.056069	0.000027	138
11	75	0.71661	0.00008	0.00021	0.002559	0.000156	0.000112	0.000011	18.0	940	0.055817	0.000035	149

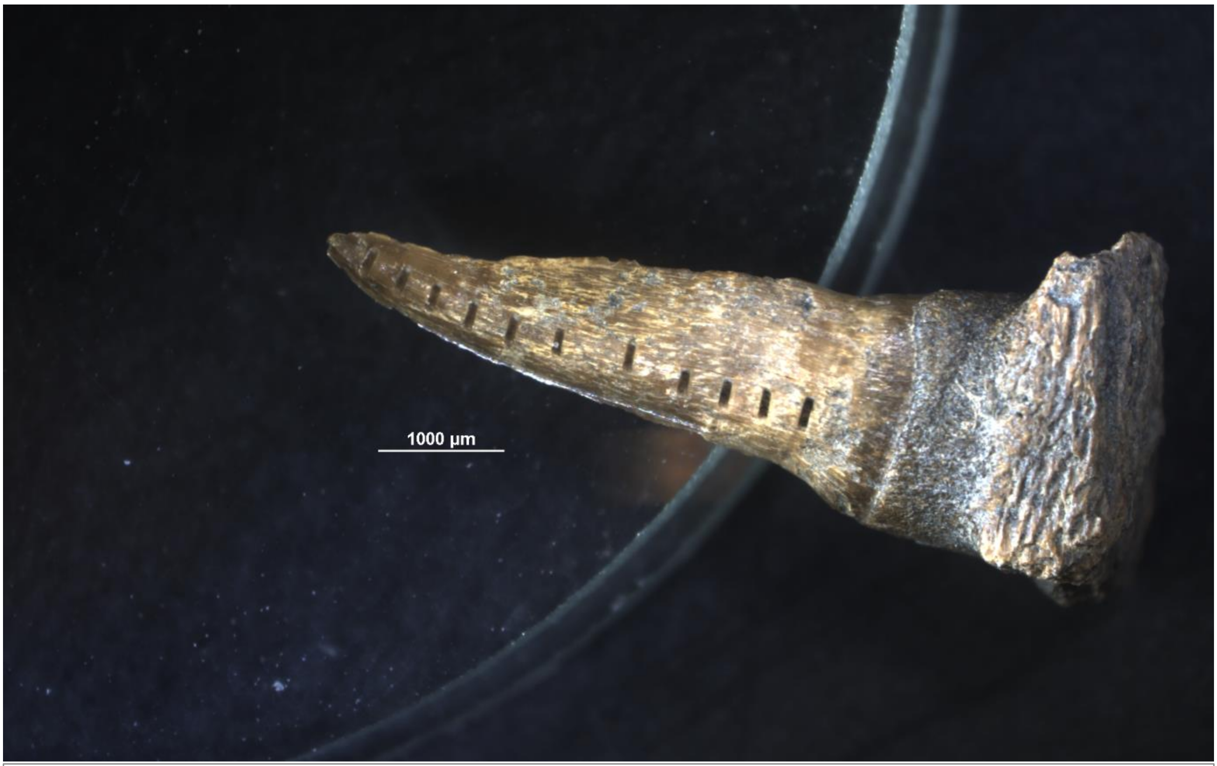

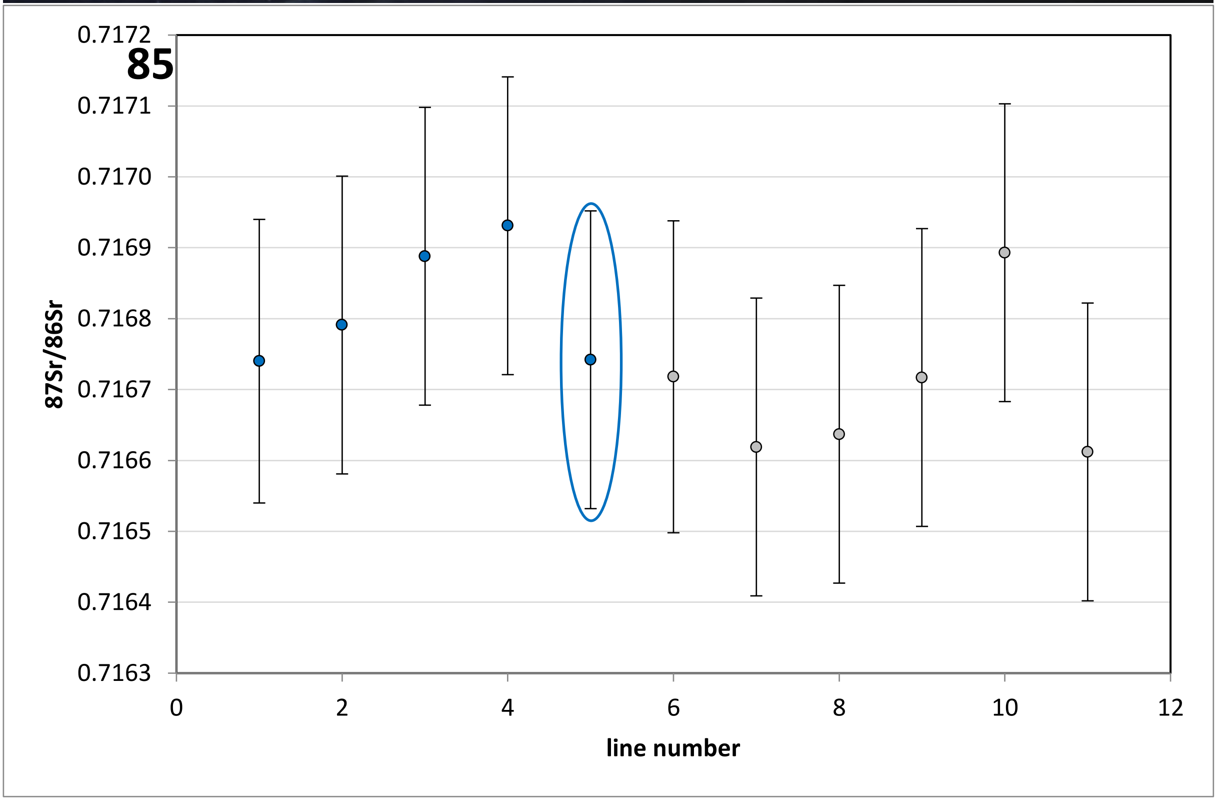

Fig S 18. Close up photograph of ablated Pike 85 tooth (upper) and unique mobility pattern (lower). Blue data point indicates ‘enamel only’ ablation; grey data point indicates enamel-dentine mix and circle around a data point indicate a degree of uncertainty to the assigned category. Photo: Adam Boethius

Pike 86 (Esox Lucius), fermentation pit

Table S 26. $^{87}Sr/^{86}Sr$ measurements for Pike 86. Coloured $^{87}Sr/^{86}Sr$ data marks 'enamel only' ablations as inferred from ocular examination of the close-up photograph and/or through changes in $^{87}Rb/^{86}Sr$ ratios. Mean $^{84}Sr/^{86}Sr$ for Pike 86 is 0.0564±0.0001 (2SD), with accepted value at 0.0565 (Thirlwall, 1991). [1]Propagated from external reproducibility (2SD) obtained from the primary standard during the analytical session combined with the within-run precision of each analysis (2SE) (Iolite Version 2.5). [2] average of total Sr beam intensity in V (sum of ^{84}Sr, ^{86}Sr, ^{87}Sr, ^{88}Sr). [3] estimated from measured Sr intensity in secondary RM using the same ablation conditions (semi-quantitative).

Line number	Sampling time (sec)	$^{87}Sr/^{86}Sr$	2SE	2SD[1]	$^{87}Rb/^{86}Sr$	2SE	$^{174}Yb^{2+}/^{86}Sr$	2SE	Mean Sr-Beam (V)[2]	Estimated Sr concentration (ppm)[3]	$^{84}Sr/^{86}Sr$	2SE	Data points / line
1	33	0.71611	0.00006	0.00015	0.000154	0.000007	0.000106	0.000014	14.1	950	0.056346	0.000041	66
2	49	0.71642	0.00006	0.00015	0.000213	0.000010	0.000137	0.000014	13.1	880	0.056322	0.000040	97
3	50	0.71638	0.00005	0.00015	0.000302	0.000017	0.000149	0.000011	13.9	940	0.056471	0.000039	99
4	75	0.71666	0.00004	0.00014	0.000359	0.000015	0.000132	0.000010	14.5	980	0.056394	0.000026	150
5	77	0.71653	0.00004	0.00014	0.000274	0.000015	0.000110	0.000009	14.8	1000	0.056464	0.000031	153
6	63	0.71644	0.00005	0.00014	0.000300	0.000016	0.000080	0.000009	14.7	990	0.056316	0.000030	127
7	63	0.71628	0.00004	0.00014	0.000330	0.000030	0.000112	0.000010	15.1	1020	0.056410	0.000033	127
8	47	0.71649	0.00005	0.00015	0.000389	0.000032	0.000109	0.000012	14.7	990	0.056373	0.000041	94
9	48	0.71647	0.00006	0.00015	0.000331	0.000022	0.000119	0.000013	14.1	950	0.056469	0.000035	96
10	56	0.71658	0.00005	0.00015	0.000277	0.000020	0.000104	0.000013	14.0	940	0.056359	0.000032	111
11	56	0.71646	0.00006	0.00015	0.000435	0.000022	0.000113	0.000012	14.2	950	0.056451	0.000034	111
12	58	0.71665	0.00005	0.00014	0.000439	0.000027	0.000110	0.000012	14.5	980	0.056472	0.000031	117
13	53	0.71675	0.00006	0.00015	0.000989	0.000071	0.000122	0.000011	14.0	940	0.056407	0.000037	106
14	53	0.71686	0.00007	0.00015	0.001267	0.000115	0.000088	0.000010	14.8	1000	0.056347	0.000037	106
15	60	0.71684	0.00006	0.00015	0.001825	0.000164	0.000158	0.000012	15.4	1040	0.056328	0.000034	120
16	60	0.71639	0.00006	0.00015	0.001222	0.000102	0.000118	0.000010	16.5	1110	0.056402	0.000029	120
17	48	0.71691	0.00013	0.00019	0.038616	0.003912	0.000205	0.000017	11.1	740	0.056251	0.000047	96

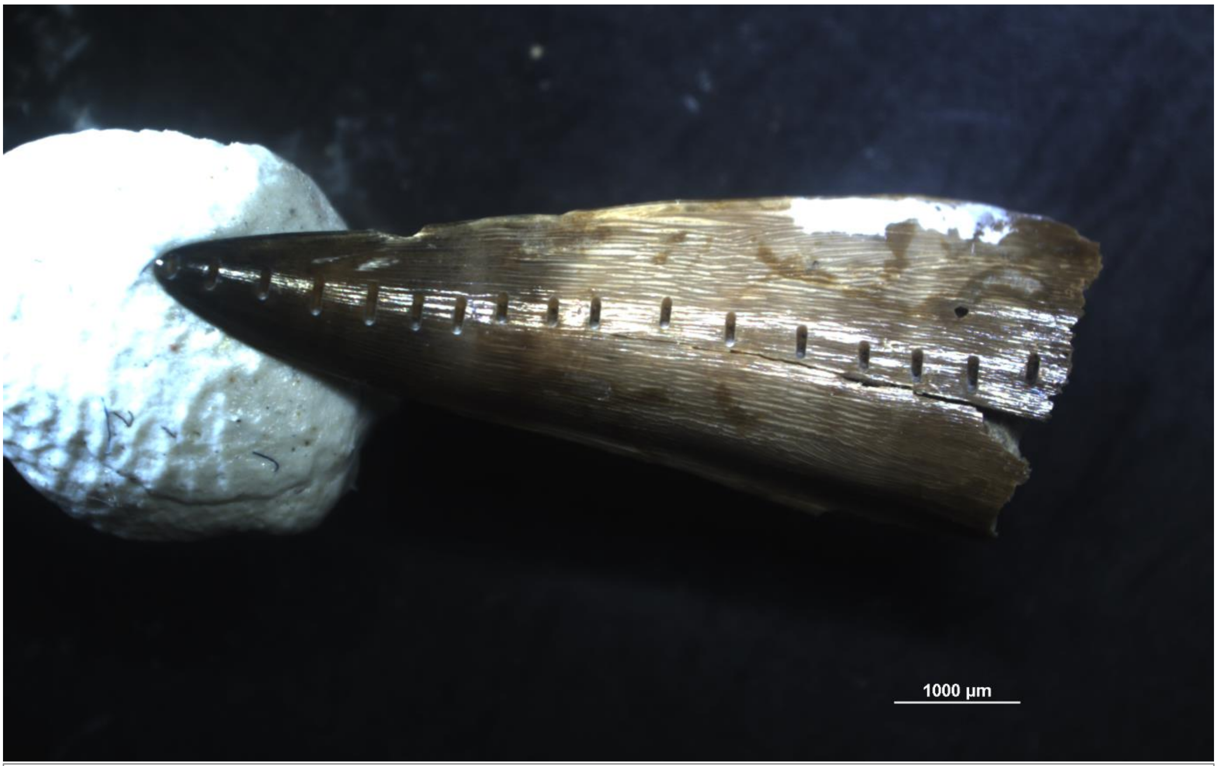

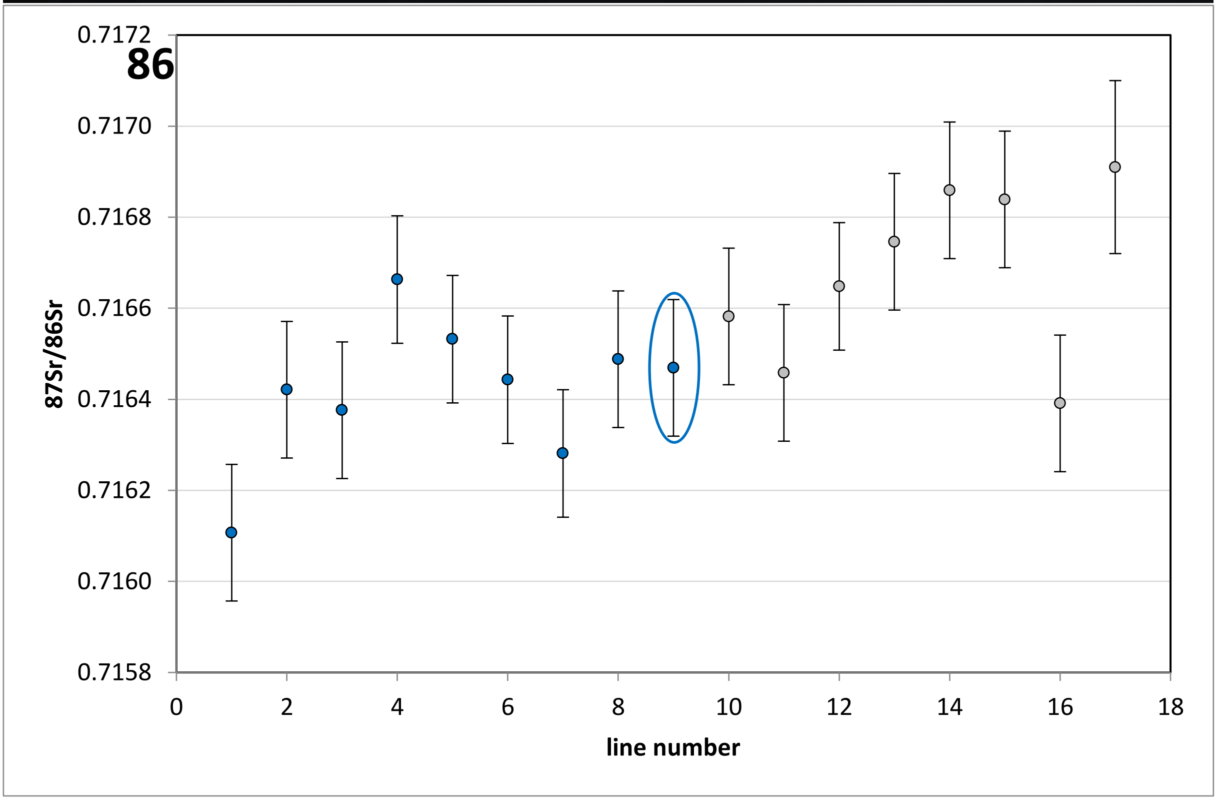

Fig S 19. Close up photograph of ablated Pike 86 tooth (upper) and unique mobility pattern (lower). Blue data point indicates ‘enamel only’ ablation; grey data point indicates enamel-dentine mix and circle around a data point indicate a degree of uncertainty to the assigned category. Photo: Adam Boethius

Pike 87 (Esox lucius). fermentation pit

Table S 27. $^{87}Sr/^{86}Sr$ measurements for Pike 87. Coloured $^{87}Sr/^{86}Sr$ data marks 'enamel only' ablations as inferred from ocular examination of the close-up photograph and/or through changes in $^{87}Rb/^{86}Sr$ ratios. Mean $^{84}Sr/^{86}Sr$ for Pike 87 is 0.0561±0.0001 (2SD), with accepted value at 0.0565 (Thirlwall, 1991). [1]Propagated from external reproducibility (2SD) obtained from the primary standard during the analytical session combined with the within-run precision of each analysis (2SE) (Iolite Version 2.5). [2] average of total Sr beam intensity in V (sum of ^{84}Sr, ^{86}Sr, ^{87}Sr, ^{88}Sr). [3] estimated from measured Sr intensity in secondary RM using the same ablation conditions (semi-quantitative).

Line number	Sampling time (sec)	$^{87}Sr/^{86}Sr$	2SE	2SD[1]	$^{87}Rb/^{86}Sr$	2SE	$^{174}Yb^{2+}/^{86}Sr$	2SE	Mean Sr-Beam (V)[2]	Estimated Sr concentration (ppm)[3]	$^{84}Sr/^{86}Sr$	2SE	Data points / line
1	38	0.71753	0.00008	0.00022	0.001232	0.000030	0.000193	0.000017	12.0	630	0.056024	0.000048	76
2	36	0.71842	0.00009	0.00022	0.002468	0.000047	0.000198	0.000018	14.1	740	0.056015	0.000043	71
3	41	0.71876	0.00007	0.00021	0.003400	0.000055	0.000193	0.000017	14.2	750	0.056091	0.000045	82
4	44	0.71887	0.00009	0.00022	0.003782	0.000046	0.000180	0.000015	14.9	780	0.056134	0.000035	88
5	44	0.71905	0.00007	0.00021	0.004400	0.000049	0.000162	0.000017	15.4	810	0.056114	0.000037	88
6	41	0.71917	0.00008	0.00022	0.004972	0.000054	0.000167	0.000015	15.1	790	0.056134	0.000041	81
7	40	0.71918	0.00011	0.00023	0.005318	0.000095	0.000157	0.000015	15.3	800	0.056153	0.000032	80
8	42	0.71880	0.00011	0.00023	0.003281	0.000200	0.000179	0.000016	15.9	830	0.056095	0.000034	84
9	49	0.71926	0.00010	0.00022	0.004814	0.000148	0.000149	0.000012	15.7	820	0.056113	0.000035	97
10	44	0.71940	0.00014	0.00024	0.005919	0.000164	0.000141	0.000014	14.9	780	0.056150	0.000037	88
11	47	0.71960	0.00009	0.00022	0.006144	0.000113	0.000148	0.000014	17.0	890	0.056204	0.000032	93
12	48	0.71989	0.00006	0.00021	0.005494	0.000129	0.000128	0.000011	19.2	1000	0.056153	0.000032	95
13	50	0.72016	0.00023	0.00030	0.003646	0.000108	0.000131	0.000011	19.0	990	0.056202	0.000031	100

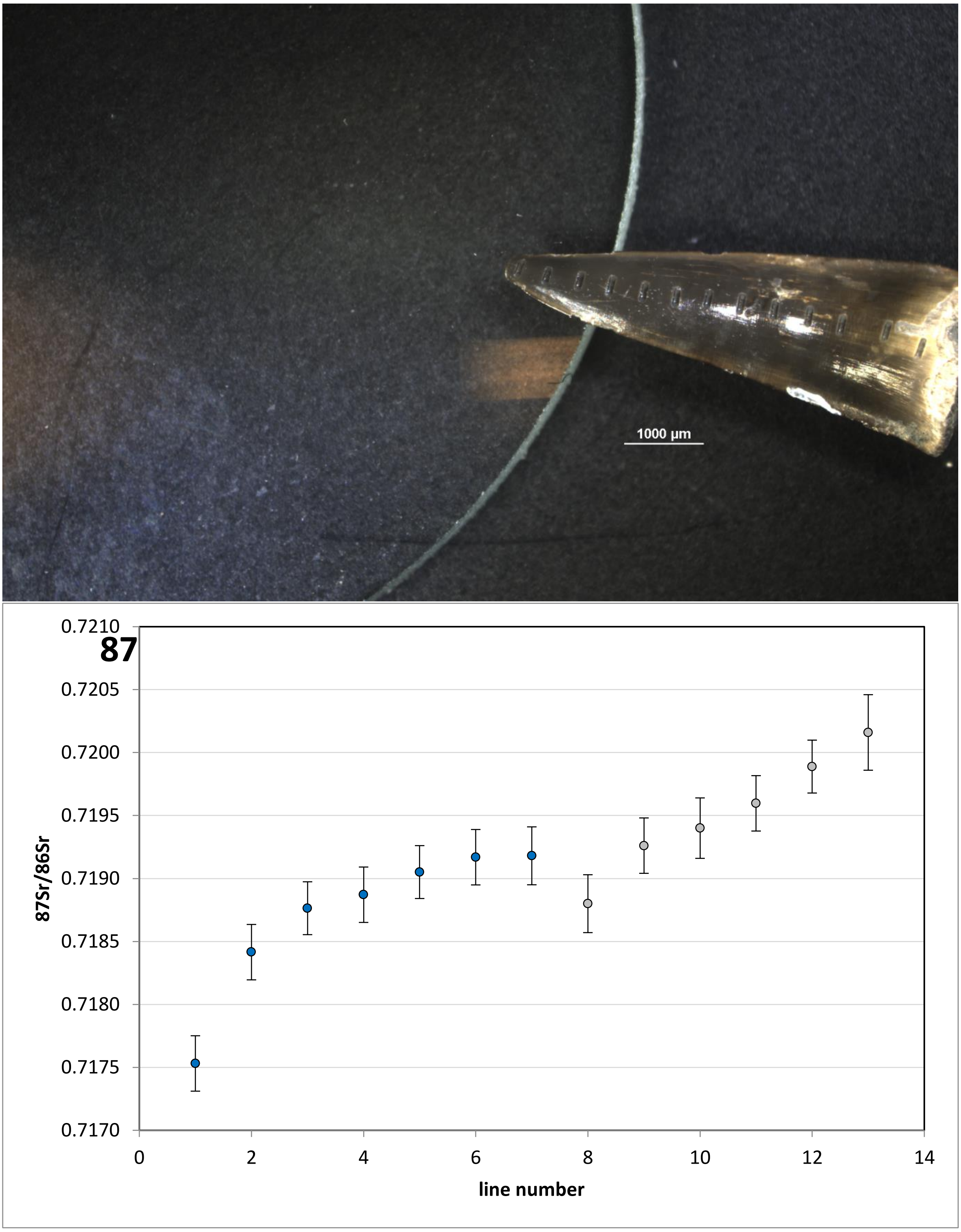

Fig S 20. Close up photograph of ablated Pike 87 tooth (upper) and unique mobility pattern (lower). Blue data point indicates ‘enamel only’ ablation; grey data point indicates enamel-dentine mix. Photo: Adam Boethius

Pike 88 (Esox lucius), fermentation pit

Table S 28. $^{87}Sr/^{86}Sr$ measurements for Pike 88. Coloured $^{87}Sr/^{86}Sr$ data marks 'enamel only' ablations as inferred from ocular examination of the close-up photograph and/or through changes in $^{87}Rb/^{86}Sr$ ratios. Mean $^{84}Sr/^{86}Sr$ for Pike 88 is 0.0561±0.0001 (2SD), with accepted value at 0.0565 (Thirlwall, 1991). [1]Propagated from external reproducibility (2SD) obtained from the primary standard during the analytical session combined with the within-run precision of each analysis (2SE) (Iolite Version 2.5). [2] average of total Sr beam intensity in V (sum of ^{84}Sr, ^{86}Sr, ^{87}Sr, ^{88}Sr). [3] estimated from measured Sr intensity in secondary RM using the same ablation conditions (semi-quantitative).

Line number	Sampling time (sec)	$^{87}Sr/^{86}Sr$	2SE	2SD[1]	$^{87}Rb/^{86}Sr$	2SE	$^{174}Yb^{2+}/^{86}Sr$	2SE	Mean Sr-Beam (V)[2]	Estimated Sr concentration (ppm)[3]	$^{84}Sr/^{86}Sr$	2SE	Data points / line
1	33	0.71719	0.00015	0.00025	0.000187	0.000017	0.000113	0.000023	17.3	910	0.056088	0.000040	65
2	38	0.71682	0.00008	0.00021	0.000189	0.000009	0.000146	0.000014	16.6	870	0.056082	0.000038	75
3	51	0.71683	0.00005	0.00021	0.000199	0.000009	0.000121	0.000011	19.9	1040	0.056097	0.000031	101
4	54	0.71678	0.00007	0.00021	0.000295	0.000013	0.000112	0.000011	18.1	950	0.056094	0.000026	107
5	45	0.71672	0.00008	0.00022	0.000216	0.000014	0.000136	0.000012	18.5	970	0.056162	0.000031	89
6	47	0.71665	0.00008	0.00022	0.000261	0.000023	0.000132	0.000012	18.8	990	0.056276	0.000026	94
7	52	0.71672	0.00010	0.00022	0.000235	0.000025	0.000113	0.000010	20.4	1070	0.056143	0.000025	103
8	49	0.71655	0.00005	0.00021	0.000105	0.000007	0.000105	0.000009	25.1	1320	0.056238	0.000021	98
9	54	0.71645	0.00006	0.00021	0.002586	0.000419	0.000101	0.000010	23.1	1210	0.056146	0.000023	108
10	49	0.71645	0.00010	0.00023	0.004435	0.000327	0.000133	0.000011	21.5	1130	0.056064	0.000029	97

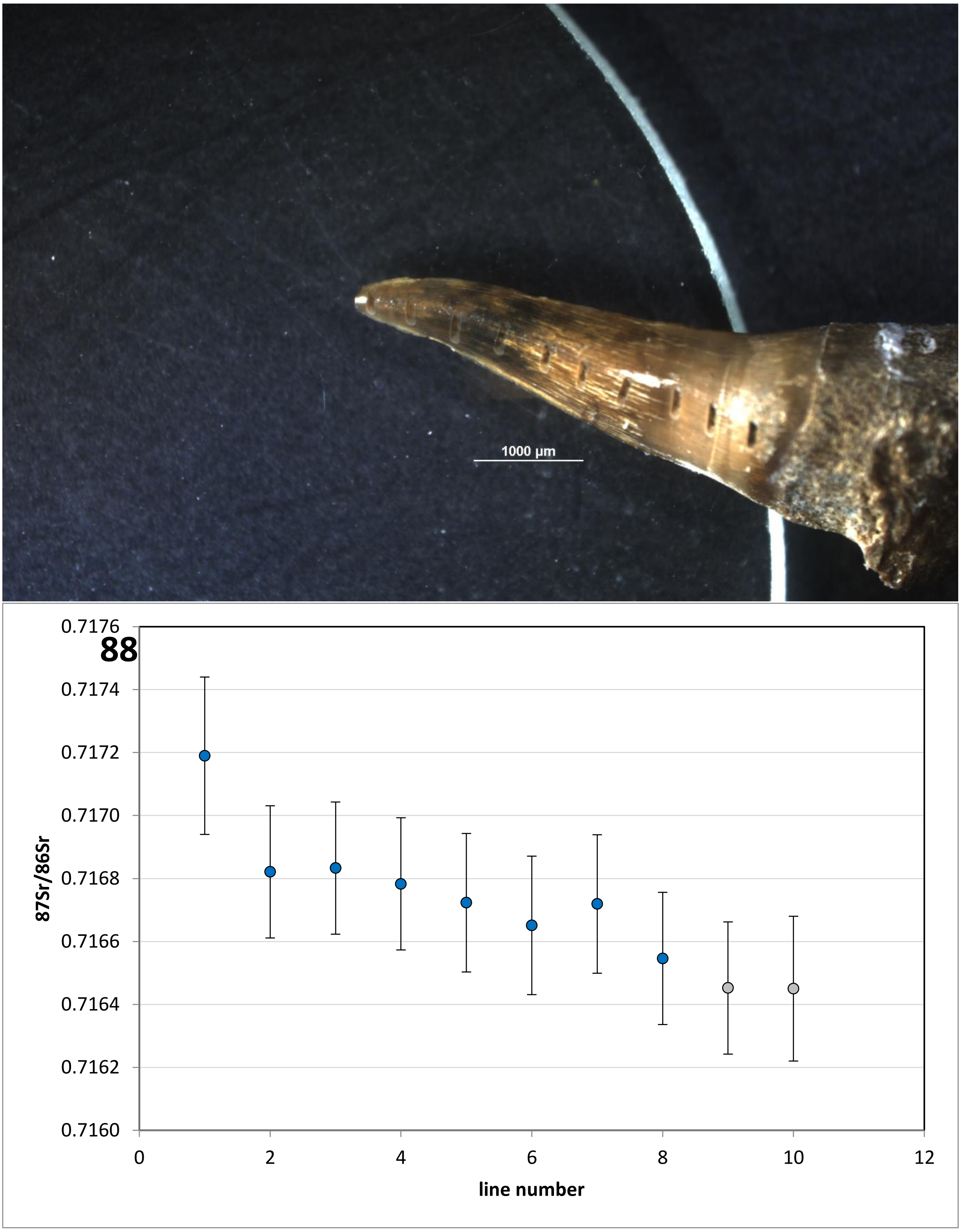

Fig S 21. Close up photograph of ablated Pike 88 tooth (upper) and unique mobility pattern (lower). Blue data point indicates 'enamel only' ablation; grey data point indicates enamel-dentine mix. Photo: Adam Boethius

Pike 89 (Esox lucius), fermentation pit

Table S 29. $^{87}Sr/^{86}Sr$ measurements for Pike 89. Coloured $^{87}Sr/^{86}Sr$ data marks 'enamel only' ablations as inferred from ocular examination of the close-up photograph and/or through changes in $^{87}Rb/^{86}Sr$ ratios. Mean $^{84}Sr/^{86}Sr$ for Pike 89 is 0.0560±0.0002 (2SD), with accepted value at 0.0565 (Thirlwall, 1991). [1]Propagated from external reproducibility (2SD) obtained from the primary standard during the analytical session combined with the within-run precision of each analysis (2SE) (Iolite Version 2.5). [2] average of total Sr beam intensity in V (sum of ^{84}Sr, ^{86}Sr, ^{87}Sr, ^{88}Sr). [3] estimated from measured Sr intensity in secondary RM using the same ablation conditions (semi-quantitative).

Line number	Sampling time (sec)	$^{87}Sr/^{86}Sr$	2SE	2SD[1]	$^{87}Rb/^{86}Sr$	2SE	$^{174}Yb^{2+}/^{86}Sr$	2SE	Mean Sr-Beam (V)[2]	Estimated Sr concentration (ppm)[3]	$^{84}Sr/^{86}Sr$	2SE	Data points / line
1	21	0.71559	0.00015	0.00025	0.000269	0.000017	0.000113	0.000025	12.3	650	0.056145	0.000067	41
2	31	0.71562	0.00010	0.00022	0.000264	0.000009	0.000148	0.000020	12.6	660	0.056011	0.000055	62
3	48	0.71557	0.00007	0.00021	0.000291	0.000013	0.000127	0.000012	14.6	760	0.056034	0.000038	95
4	48	0.71551	0.00006	0.00021	0.000357	0.000019	0.000132	0.000015	14.4	760	0.056077	0.000044	96
5	46	0.71562	0.00006	0.00021	0.000647	0.000032	0.000130	0.000015	16.2	850	0.056037	0.000034	92
6	49	0.71574	0.00006	0.00021	0.000733	0.000030	0.000099	0.000012	17.9	940	0.056182	0.000034	97
7	50	0.71581	0.00006	0.00021	0.000653	0.000028	0.000111	0.000011	19.0	1000	0.056092	0.000028	100
8	43	0.71565	0.00009	0.00022	0.000743	0.000073	0.000130	0.000013	18.6	980	0.056018	0.000036	85
9	50	0.71535	0.00014	0.00024	0.016757	0.001291	0.000148	0.000016	15.9	830	0.055933	0.000037	100
10	46	0.71546	0.00011	0.00023	0.011175	0.001125	0.000125	0.000014	13.2	690	0.055893	0.000042	91

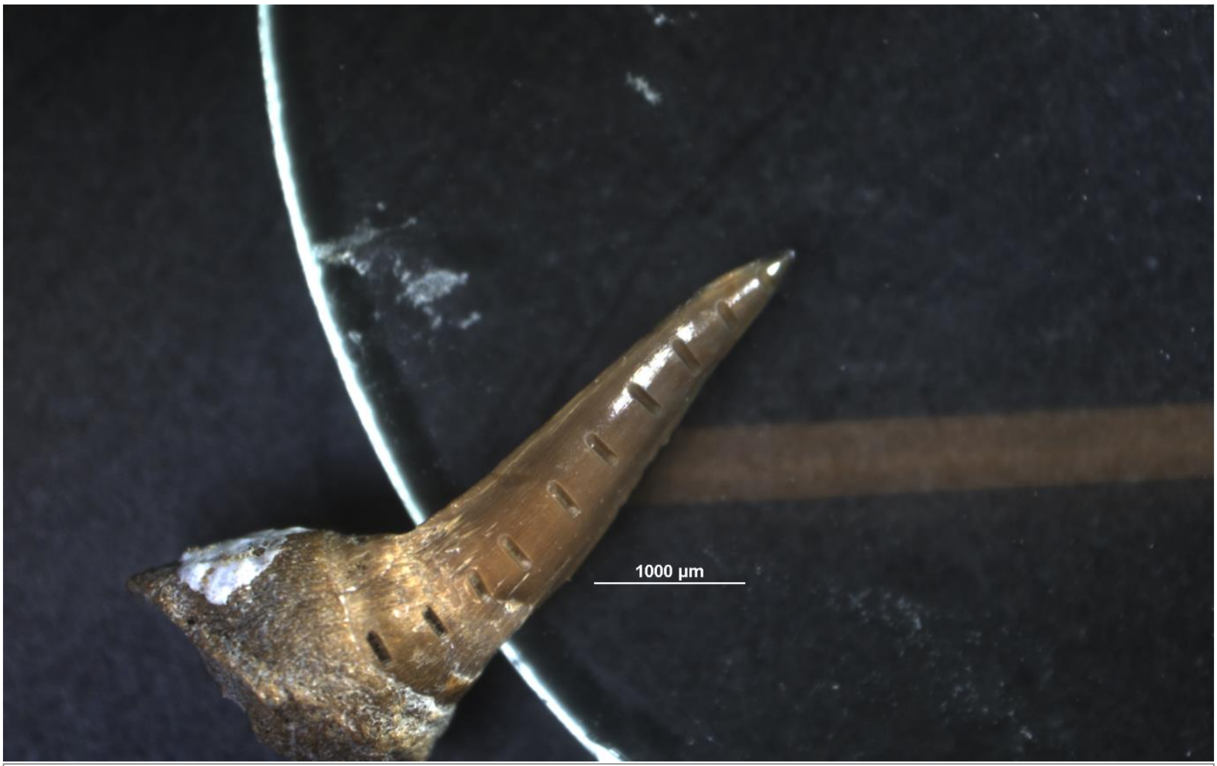

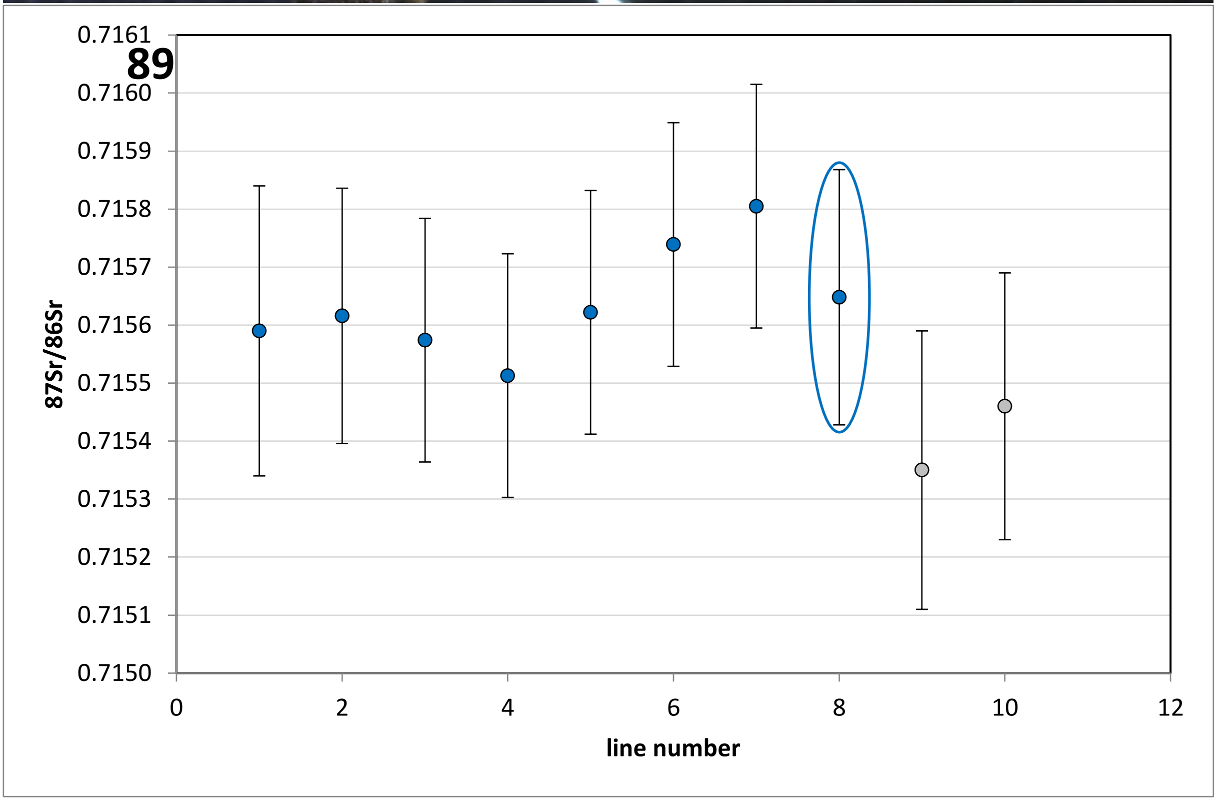

Fig S 22. Close up photograph of ablated Pike 89 tooth (upper) and unique mobility pattern (lower). Blue data point indicates ‘enamel only’ ablation; grey data point indicates enamel-dentine mix and circle around a data point indicate a degree of uncertainty to the assigned category. Photo: Adam Boethius

Pike 92 (Esox lucius), other areas

Table S 30. $^{87}Sr/^{86}Sr$ measurements for Pike 92. Coloured $^{87}Sr/^{86}Sr$ data marks 'enamel only' ablations as inferred from ocular examination of the close-up photograph and/or through changes in $^{87}Rb/^{86}Sr$ ratios. Mean $^{84}Sr/^{86}Sr$ for Pike 92 is 0.0563±0.0001 (2SD), with accepted value at 0.0565 (Thirlwall, 1991). [1]Propagated from external reproducibility (2SD) obtained from the primary standard during the analytical session combined with the within-run precision of each analysis (2SE) (Iolite Version 2.5). [2] average of total Sr beam intensity in V (sum of ^{84}Sr, ^{86}Sr, ^{87}Sr, ^{88}Sr). [3] estimated from measured Sr intensity in secondary RM using the same ablation conditions (semi-quantitative).

Line number	Sampling time (sec)	$^{87}Sr/^{86}Sr$	2SE	2SD[1]	$^{87}Rb/^{86}Sr$	2SE	$^{174}Yb^{2+}/^{86}Sr$	2SE	Mean Sr-Beam (V)[2]	Estimated Sr concentration (ppm)[3]	$^{84}Sr/^{86}Sr$	2SE	Data points / line
1	23	0.71450	0.00014	0.00021	0.000489	0.000017	0.000066	0.000025	10.4	760	0.056268	0.000081	46
2	32	0.71437	0.00009	0.00018	0.000487	0.000027	0.000144	0.000026	11.0	810	0.056415	0.000046	64
3	36	0.71481	0.00015	0.00022	0.000728	0.000167	0.000130	0.000023	12.0	880	0.056373	0.000068	73
4	36	0.71484	0.00011	0.00019	0.000555	0.000020	0.000123	0.000016	12.6	920	0.056390	0.000055	73
5	37	0.71464	0.00007	0.00017	0.000795	0.000037	0.000115	0.000017	12.3	900	0.056265	0.000048	75
6	36	0.71476	0.00008	0.00018	0.000902	0.000039	0.000103	0.000019	12.7	930	0.056288	0.000038	71
7	40	0.71478	0.00007	0.00017	0.001227	0.000052	0.000121	0.000017	12.8	940	0.056270	0.000050	79
8	41	0.71456	0.00012	0.00020	0.001548	0.000060	0.000098	0.000015	13.7	1000	0.056294	0.000044	82
9	39	0.71474	0.00011	0.00019	0.001955	0.000162	0.000088	0.000015	12.5	920	0.056271	0.000051	78
10	40	0.71498	0.00014	0.00021	0.004234	0.000241	0.000140	0.000015	12.4	910	0.056259	0.000044	79
11	43	0.71519	0.00012	0.00020	0.007330	0.000317	0.000119	0.000014	12.2	890	0.056199	0.000045	85
12	51	0.71467	0.00009	0.00018	0.002676	0.000200	0.000109	0.000013	12.7	930	0.056163	0.000044	102
13	48	0.71432	0.00014	0.00021	0.005392	0.000490	0.000088	0.000016	12.5	910	0.056250	0.000044	96

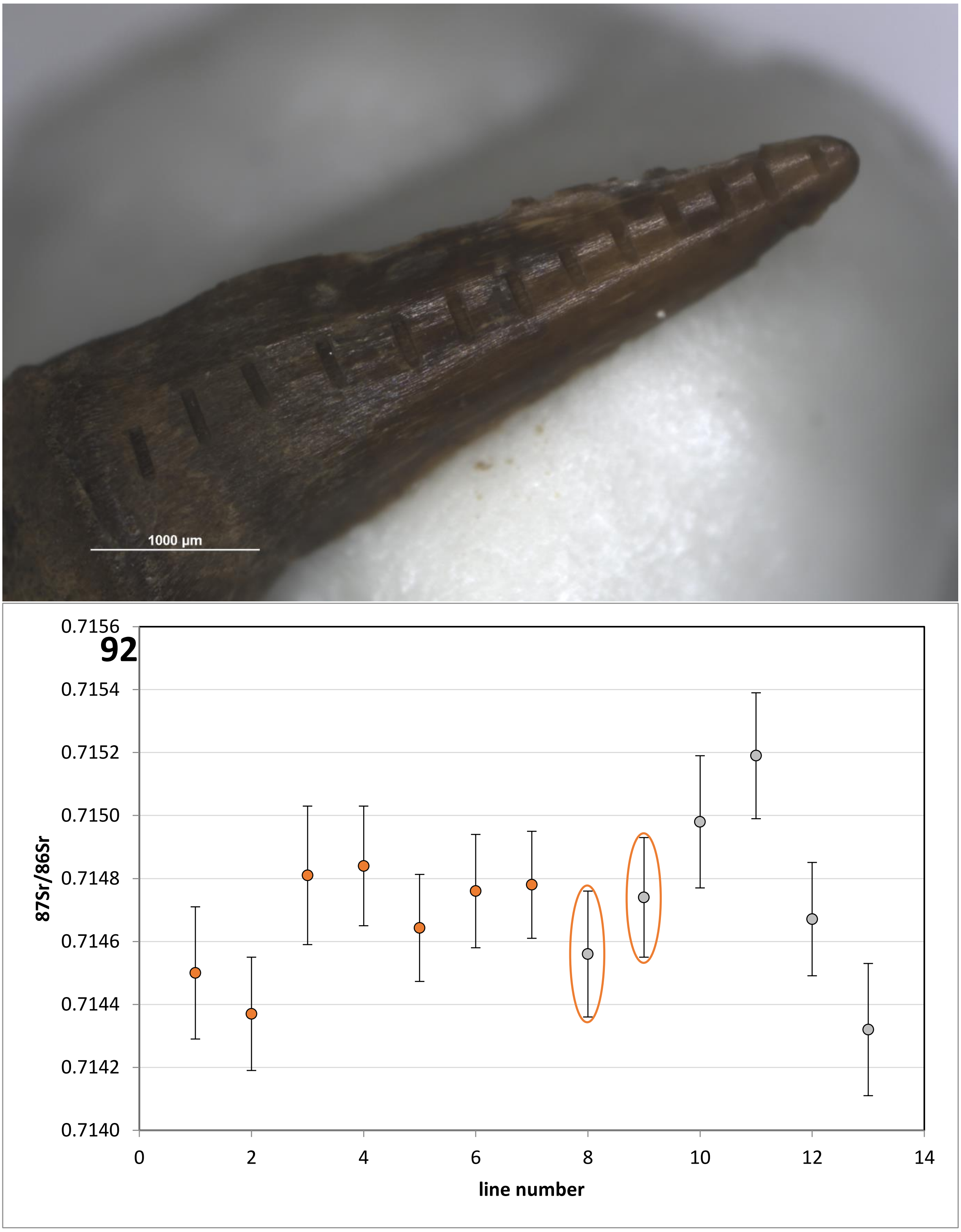

Fig S 23. Close up photograph of ablated Pike 92 tooth (upper) and unique mobility pattern (lower). Orange data point indicates ‘enamel only’ ablation; grey data point indicates enamel-dentine mix and circle around a data point indicate a degree of uncertainty to the assigned category. Photo: Adam Boethius

Pike 93 (Esox lucius), other areas

Table S 31. $^{87}Sr/^{86}Sr$ measurements for Pike 93. Coloured $^{87}Sr/^{86}Sr$ data marks 'enamel only' ablations as inferred from ocular examination of the close-up photograph and/or through changes in $^{87}Rb/^{86}Sr$ ratios. Mean $^{84}Sr/^{86}Sr$ for Pike 93 is 0.0562±0.0002 (2SD), with accepted value at 0.0565 (Thirlwall, 1991). [1]Propagated from external reproducibility (2SD) obtained from the primary standard during the analytical session combined with the within-run precision of each analysis (2SE) (Iolite Version 2.5). [2] average of total Sr beam intensity in V (sum of ^{84}Sr, ^{86}Sr, ^{87}Sr, ^{88}Sr). [3] estimated from measured Sr intensity in secondary RM using the same ablation conditions (semi-quantitative).

Line number	Sampling time (sec)	$^{87}Sr/^{86}Sr$	2SE	2SD[1]	$^{87}Rb/^{86}Sr$	2SE	$^{174}Yb^{2+}/^{86}Sr$	2SE	Mean Sr-Beam (V)[2]	Estimated Sr concentration (ppm)[3]	$^{84}Sr/^{86}Sr$	2SE	Data points / line
1	34	0.71500	0.00005	0.00024	0.000221	0.000005	0.000077	0.000011	22.4	1110	0.056257	0.000029	68
2	41	0.71514	0.00004	0.00024	0.000193	0.000003	0.000120	0.000009	23.0	1140	0.056344	0.000024	83
3	34	0.71516	0.00004	0.00024	0.000184	0.000003	0.000112	0.000010	22.7	1130	0.056284	0.000026	69
4	36	0.71512	0.00004	0.00024	0.000161	0.000003	0.000108	0.000010	23.4	1160	0.056329	0.000031	73
5	45	0.71501	0.00004	0.00024	0.000131	0.000003	0.000099	0.000008	24.7	1230	0.056271	0.000022	91
6	42	0.71503	0.00004	0.00024	0.000121	0.000002	0.000093	0.000008	25.8	1280	0.056238	0.000025	83
7	36	0.71527	0.00006	0.00024	0.000112	0.000003	0.000088	0.000010	23.7	1180	0.056226	0.000027	72
8	43	0.71516	0.00005	0.00024	0.000136	0.000006	0.000113	0.000009	23.2	1150	0.056218	0.000024	87
9	45	0.71526	0.00007	0.00025	0.000133	0.000005	0.000102	0.000010	22.7	1130	0.056176	0.000025	90
10	47	0.71522	0.00006	0.00024	0.000149	0.000013	0.000111	0.000009	23.1	1150	0.056141	0.000024	93
11	41	0.71521	0.00004	0.00024	0.000130	0.000005	0.000126	0.000007	23.7	1180	0.056213	0.000024	83
12	43	0.71508	0.00005	0.00024	0.000172	0.000012	0.000109	0.000009	24.2	1200	0.056235	0.000023	87
13	48	0.71505	0.00003	0.00024	0.000125	0.000003	0.000094	0.000011	22.8	1140	0.056199	0.000022	96
14	41	0.71502	0.00004	0.00024	0.000121	0.000003	0.000123	0.000010	22.8	1130	0.056206	0.000027	82
15	43	0.71508	0.00003	0.00024	0.000175	0.000009	0.000092	0.000008	23.5	1170	0.056199	0.000026	86
16	51	0.71505	0.00003	0.00024	0.000172	0.000009	0.000113	0.000008	22.5	1120	0.056173	0.000021	101
17	42	0.71498	0.00004	0.00024	0.000185	0.000013	0.000101	0.000009	23.2	1160	0.056176	0.000027	84
18	47	0.71511	0.00003	0.00024	0.000176	0.000012	0.000124	0.000009	23.5	1170	0.056172	0.000021	94
19	49	0.71509	0.00003	0.00024	0.000169	0.000006	0.000103	0.000009	22.6	1130	0.056176	0.000025	98
20	44	0.71516	0.00004	0.00024	0.000202	0.000009	0.000128	0.000009	22.0	1090	0.056133	0.000027	87
21	43	0.71516	0.00004	0.00024	0.000326	0.000027	0.000126	0.000009	23.0	1150	0.055964	0.000028	87
22	40	0.71512	0.00004	0.00024	0.001038	0.000075	0.000122	0.000010	23.7	1180	0.055961	0.000022	80
23	50	0.71504	0.00006	0.00024	0.001897	0.000141	0.000098	0.000008	22.4	1120	0.055931	0.000024	100

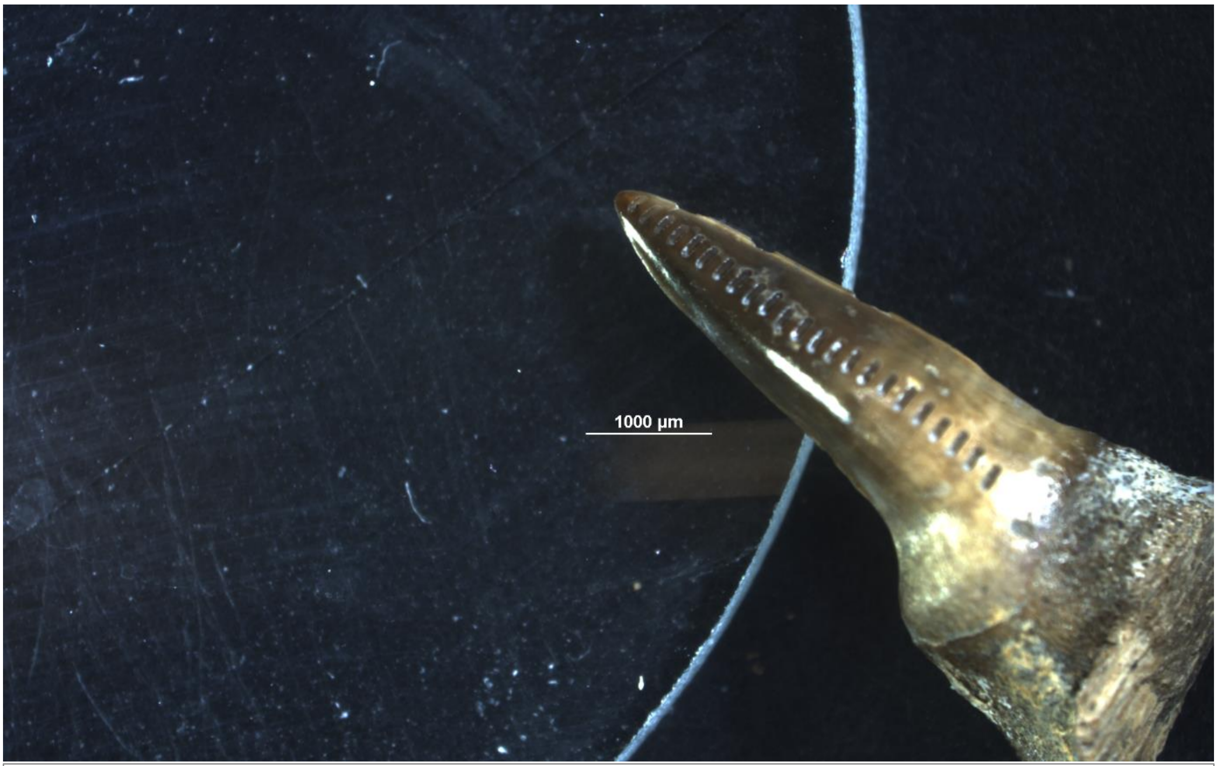

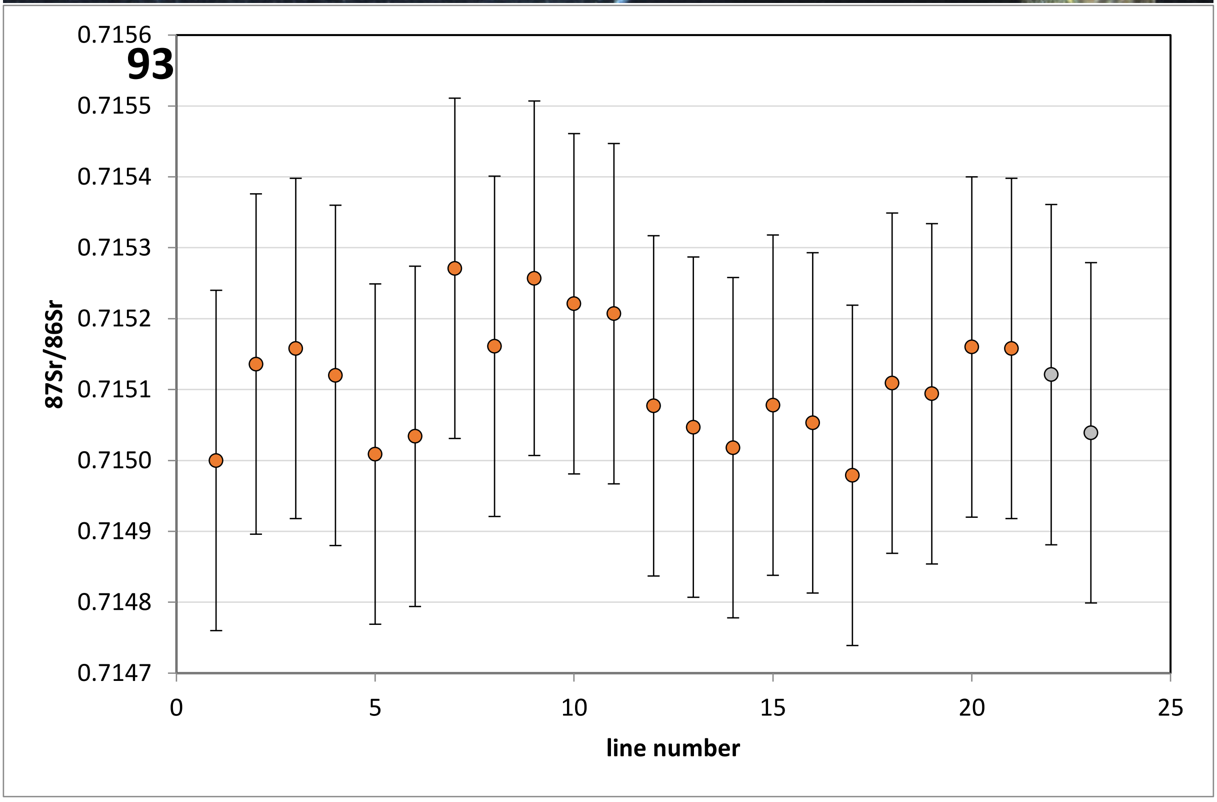

Fig S 24. Close up photograph of ablated Pike 93 tooth (upper) and unique mobility pattern (lower). Orange data point indicates 'enamel only' ablation; grey data point indicates enamel-dentine mix. Photo: Adam Boethius

Pike 94 (Esox lucius), other areas

Table S 32. $^{87}Sr/^{86}Sr$ measurements for Pike 94. Coloured $^{87}Sr/^{86}Sr$ data marks 'enamel only' ablations as inferred from ocular examination of the close-up photograph and/or through changes in $^{87}Rb/^{86}Sr$ ratios. Mean $^{84}Sr/^{86}Sr$ for Pike 94 is 0.0565±0.0001 (2SD), with accepted value at 0.0565 (Thirlwall, 1991). [1]Propagated from external reproducibility (2SD) obtained from the primary standard during the analytical session combined with the within-run precision of each analysis (2SE) (Iolite Version 2.5). [2] average of total Sr beam intensity in V (sum of ^{84}Sr, ^{86}Sr, ^{87}Sr, ^{88}Sr). [3] estimated from measured Sr intensity in secondary RM using the same ablation conditions (semi-quantitative).

Line number	Sampling time (sec)	$^{87}Sr/^{86}Sr$	2SE	2SD[1]	$^{87}Rb/^{86}Sr$	2SE	$^{174}Yb^{2+}/^{86}Sr$	2SE	Mean Sr-Beam (V)[2]	Estimated Sr concentration (ppm)[3]	$^{84}Sr/^{86}Sr$	2SE	Data points / line
1	26	0.71438	0.00006	0.00015	0.000270	0.000014	0.001385	0.000034	17.3	1160	0.056452	0.000051	52
2	34	0.71439	0.00004	0.00014	0.000303	0.000010	0.001295	0.000041	16.7	1120	0.056503	0.000042	68
3	48	0.71436	0.00005	0.00014	0.000325	0.000018	0.001377	0.000039	17.2	1160	0.056532	0.000034	96
4	55	0.71442	0.00004	0.00014	0.000335	0.000010	0.001111	0.000018	16.9	1140	0.056442	0.000035	110
5	57	0.71444	0.00004	0.00014	0.000421	0.000020	0.001103	0.000016	17.3	1160	0.056472	0.000032	115
6	46	0.71436	0.00004	0.00014	0.000535	0.000016	0.001122	0.000023	16.9	1140	0.056435	0.000037	92
7	43	0.71437	0.00005	0.00014	0.000523	0.000021	0.001132	0.000027	17.1	1150	0.056486	0.000037	87
8	43	0.71432	0.00006	0.00015	0.000887	0.000045	0.001201	0.000035	16.6	1120	0.056525	0.000036	85
9	42	0.71427	0.00005	0.00014	0.000936	0.000046	0.001201	0.000029	16.9	1130	0.056450	0.000036	83
10	51	0.71417	0.00006	0.00015	0.001116	0.000056	0.001314	0.000021	16.8	1130	0.056438	0.000029	103
11	51	0.71409	0.00005	0.00014	0.000845	0.000047	0.001186	0.000020	16.1	1080	0.056376	0.000039	103
12	45	0.71411	0.00007	0.00015	0.000808	0.000036	0.001164	0.000021	14.8	1000	0.056442	0.000041	90
13	50	0.71405	0.00009	0.00016	0.004056	0.000881	0.001238	0.000026	15.6	1050	0.056459	0.000031	99
14	44	0.71415	0.00007	0.00015	0.005431	0.000557	0.001517	0.000031	16.7	1120	0.056487	0.000041	89

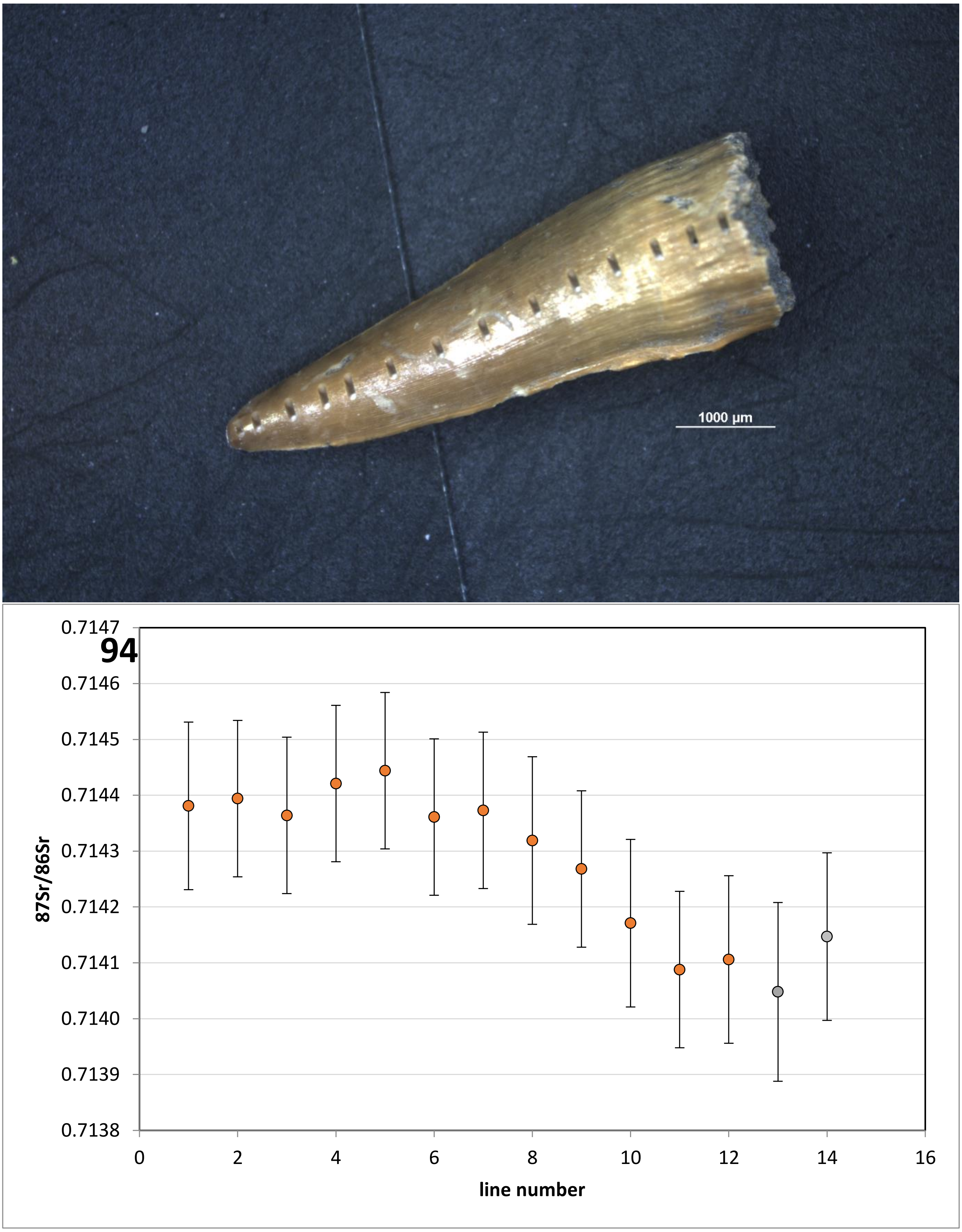

Fig S 25. Close up photograph of ablated Pike 94 tooth (upper) and unique mobility pattern (lower). Orange data point indicates 'enamel only' ablation; grey data point indicates enamel-dentine mix. Photo: Adam Boethius

Water vole (Arvicola amphibious)

Table S 33. $^{87}Sr/^{86}Sr$ measurements for Water vole 307. Mean $^{84}Sr/^{86}Sr$ is 0.0561±0.0002 (2SD), with accepted value at 0.0565 (Thirlwall, 1991). [1]Propagated from external reproducibility (2SD) obtained from the primary standard during the analytical session combined with the within-run precision of each analysis (2SE) (Iolite Version 2.5). [2] average of total Sr beam intensity in V (sum of ^{84}Sr, ^{86}Sr, ^{87}Sr, ^{88}Sr). [3] estimated from measured Sr intensity in secondary RM using the same ablation conditions (semi-quantitative).

Line number	Sampling time (sec)	$^{87}Sr/^{86}Sr$	2SE	2SD[1]	$^{87}Rb/^{86}Sr$	2SE	$^{174}Yb^{2+}/^{86}Sr$	2SE	Mean Sr-Beam (V)[2]	Estimated Sr concentration (ppm)[3]	$^{84}Sr/^{86}Sr$	2SE	Data points / line
1	33	0.71949	0.00012	0.00028	0.000801	0.000039	0.000322	0.000056	3.8	197	0.05595	0.00017	65
2	35	0.71923	0.00010	0.00027	0.000951	0.000023	0.000419	0.000053	3.8	196	0.05610	0.00016	70
3	27	0.71892	0.00015	0.00030	0.000751	0.000032	0.000428	0.000077	3.4	178	0.05610	0.00020	54

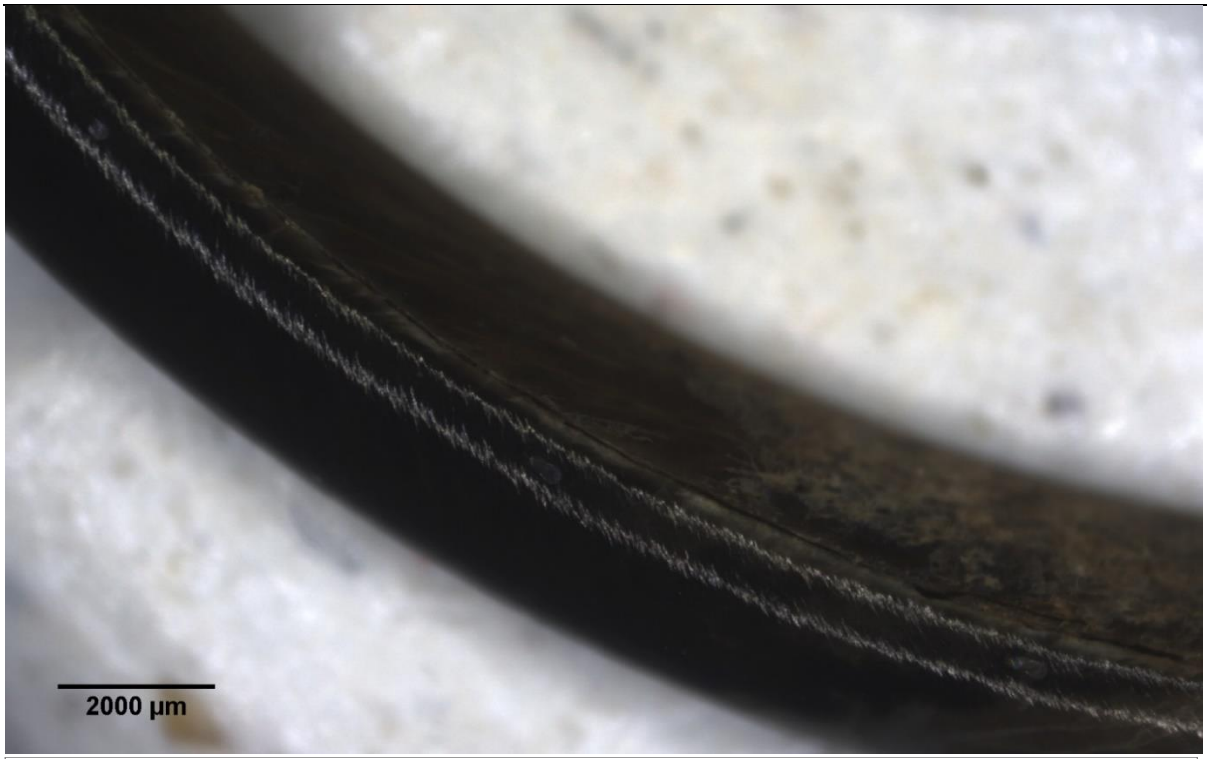

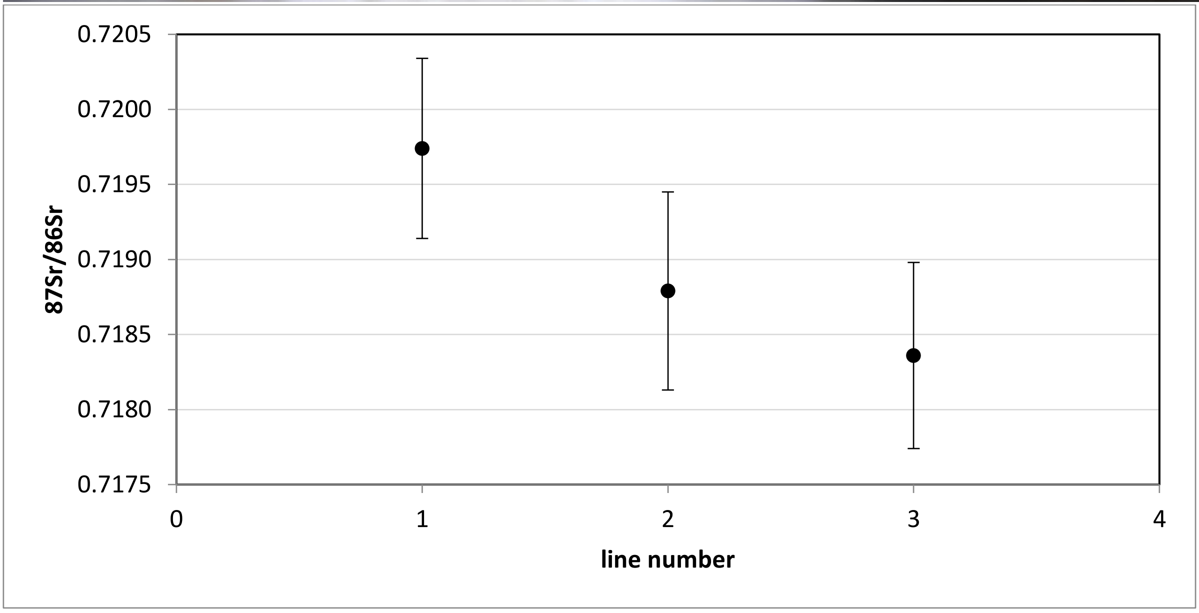

Fig S 26. Close up photograph of ablated Water vole 307 tooth (upper) and unique mobility pattern (lower). All black data points indicate 'enamel only' ablations. Photo: Adam Boethius

Table S 34. $^{87}Sr/^{86}Sr$ measurements for Water vole 308. Mean $^{84}Sr/^{86}Sr$ is 0.0561±0.0018 (2SD), with accepted value at 0.0565 (Thirlwall, 1991). [1]Propagated from external reproducibility (2SD) obtained from the primary standard during the analytical session combined with the within-run precision of each analysis (2SE) (Iolite Version 2.5). [2] average of total Sr beam intensity in V (sum of ^{84}Sr, ^{86}Sr, ^{87}Sr, ^{88}Sr). [3] estimated from measured Sr intensity in secondary RM using the same ablation conditions (semi-quantitative).

Line number	Sampling time (sec)	$^{87}Sr/^{86}Sr$	2SE	2SD[1]	$^{87}Rb/^{86}Sr$	2SE	$^{174}Yb^{2+}/^{86}Sr$	2SE	Mean Sr-Beam (V)[2]	Estimated Sr concentration (ppm)[3]	$^{84}Sr/^{86}Sr$	2SE	Data points / line
1	28	0.72096	0.00012	0.00028	0.00199	0.00003	0.00051	0.00005	2.8	146	0.05591	0.00021	55
2	34	0.72087	0.00010	0.00027	0.00202	0.00006	0.00107	0.00010	2.4	126	0.05524	0.00028	69
3	35	0.72144	0.00012	0.00028	0.00182	0.00003	0.00055	0.00008	2.2	117	0.05704	0.00026	71

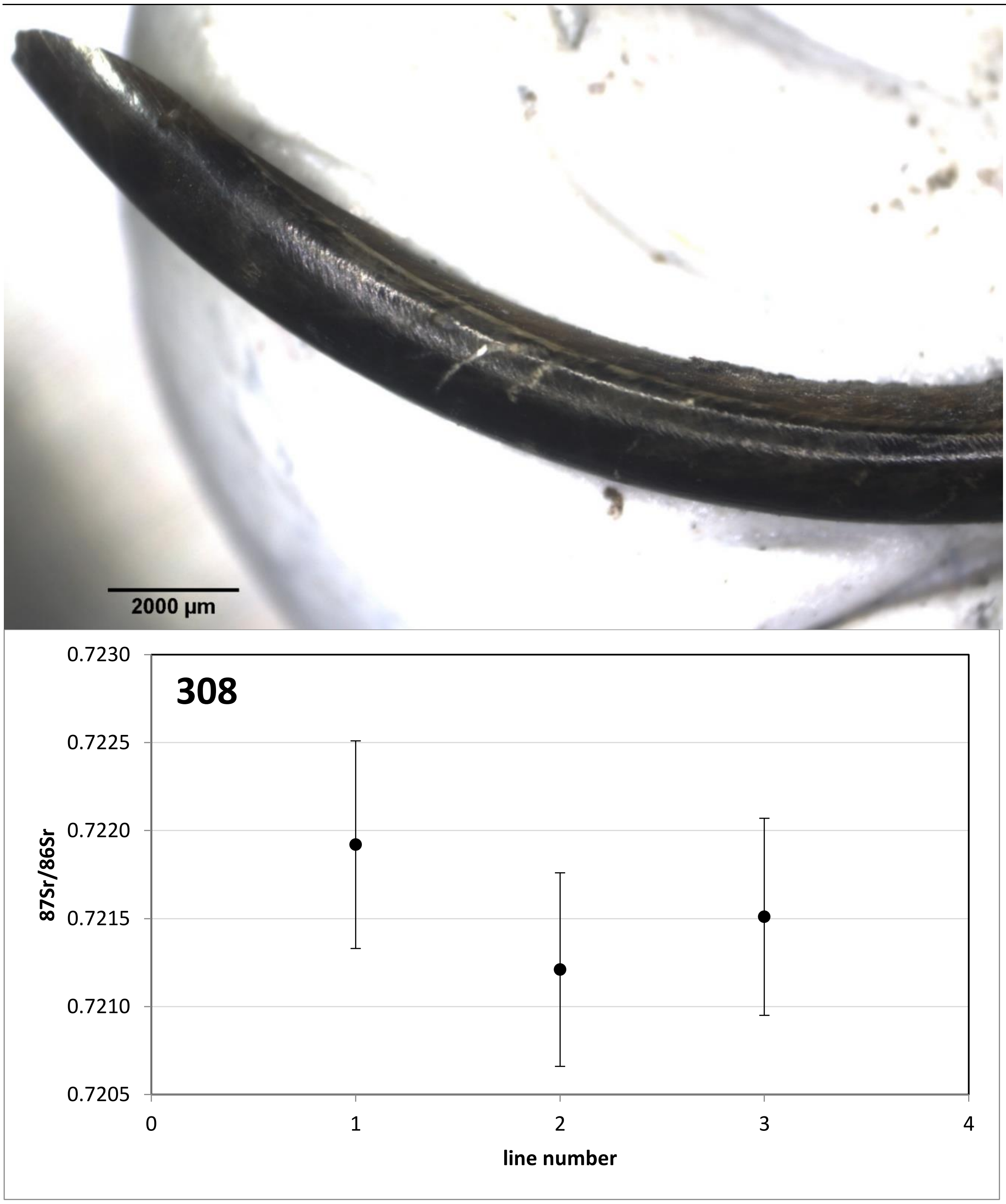

Fig S 27 Close up photograph of ablated Water vole 308 tooth (upper) and unique mobility pattern (lower). All black data points indicate 'enamel only' ablations. Photo: Adam Boethius

Table S 35. $^{87}Sr/^{86}Sr$ measurements for Water vole 347. Mean $^{84}Sr/^{86}Sr$ is 0.0565±0.0001 (2SD), with accepted value at 0.0565 (Thirlwall, 1991). [1]Propagated from external reproducibility (2SD) obtained from the primary standard during the analytical session combined with the within-run precision of each analysis (2SE) (Iolite Version 2.5). [2] average of total Sr beam intensity in V (sum of ^{84}Sr, ^{86}Sr, ^{87}Sr, ^{88}Sr). [3] estimated from measured Sr intensity in secondary RM using the same ablation conditions (semi-quantitative).

Line number	Sampling time (sec)	$^{87}Sr/^{86}Sr$	2SE	2SD[1]	$^{87}Rb/^{86}Sr$	2SE	$^{174}Yb^{2+}/^{86}Sr$	2SE	Mean Sr-Beam (V)[2]	Estimated Sr concentration (ppm)[3]	$^{84}Sr/^{86}Sr$	2SE	Data points / line
1	40	0.71955	0.00009	0.00018	0.000784	0.000049	0.000193	0.000039	5.5	307	0.05646	0.00009	80
2	49	0.71996	0.00009	0.00018	0.000611	0.000022	0.000220	0.000035	5.4	307	0.05645	0.00008	97
3	40	0.71935	0.00008	0.00017	0.001061	0.000021	0.000232	0.000046	5.2	294	0.05652	0.00010	80

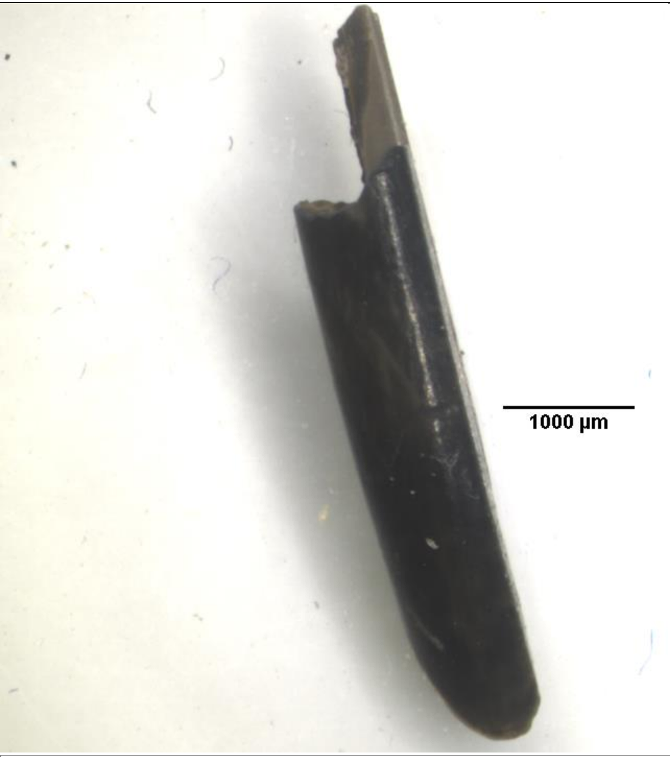

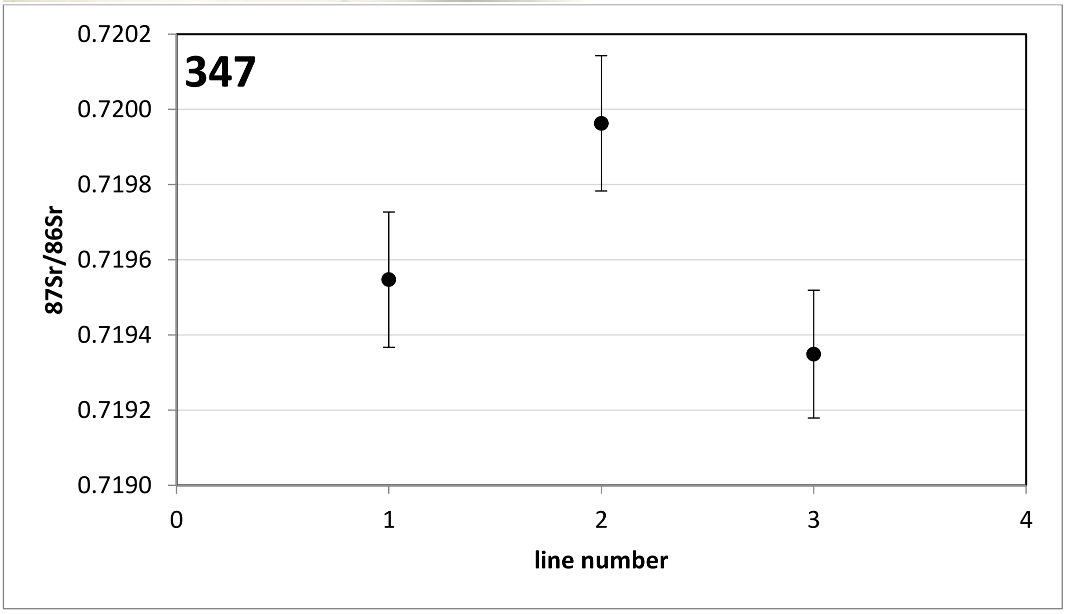

Fig S 28 Close up photograph of ablated Water vole 347 tooth (upper) and unique mobility pattern (lower). All black data points indicate 'enamel only' ablations. Photo: Adam Boethius

Table S 36. $^{87}Sr/^{86}Sr$ measurements for Water vole 348. Mean $^{84}Sr/^{86}Sr$ is 0.0563±0.0002 (2SD), with accepted value at 0.0565 (Thirlwall, 1991). [1]Propagated from external reproducibility (2SD) obtained from the primary standard during the analytical session combined with the within-run precision of each analysis (2SE) (Iolite Version 2.5). [2] average of total Sr beam intensity in V (sum of ^{84}Sr, ^{86}Sr, ^{87}Sr, ^{88}Sr). [3] estimated from measured Sr intensity in secondary RM using the same ablation conditions (semi-quantitative).

Line number	Sampling time (sec)	$^{87}Sr/^{86}Sr$	2SE	2SD[1]	$^{87}Rb/^{86}Sr$	2SE	$^{174}Yb^{2+}/^{86}Sr$	2SE	Mean Sr-Beam (V)[2]	Estimated Sr concentration (ppm)[3]	$^{84}Sr/^{86}Sr$	2SE	Data points / line
1	60	0.72054	0.00008	0.00017	0.00049	0.00004	0.00022	0.00003	5.0	282	0.05624	0.00012	119
2	59	0.72059	0.00007	0.00017	0.00045	0.00001	0.00019	0.00004	5.1	288	0.05646	0.00011	117
3	50	0.72048	0.00008	0.00017	0.00041	0.00002	0.00017	0.00004	4.9	275	0.05631	0.00009	99

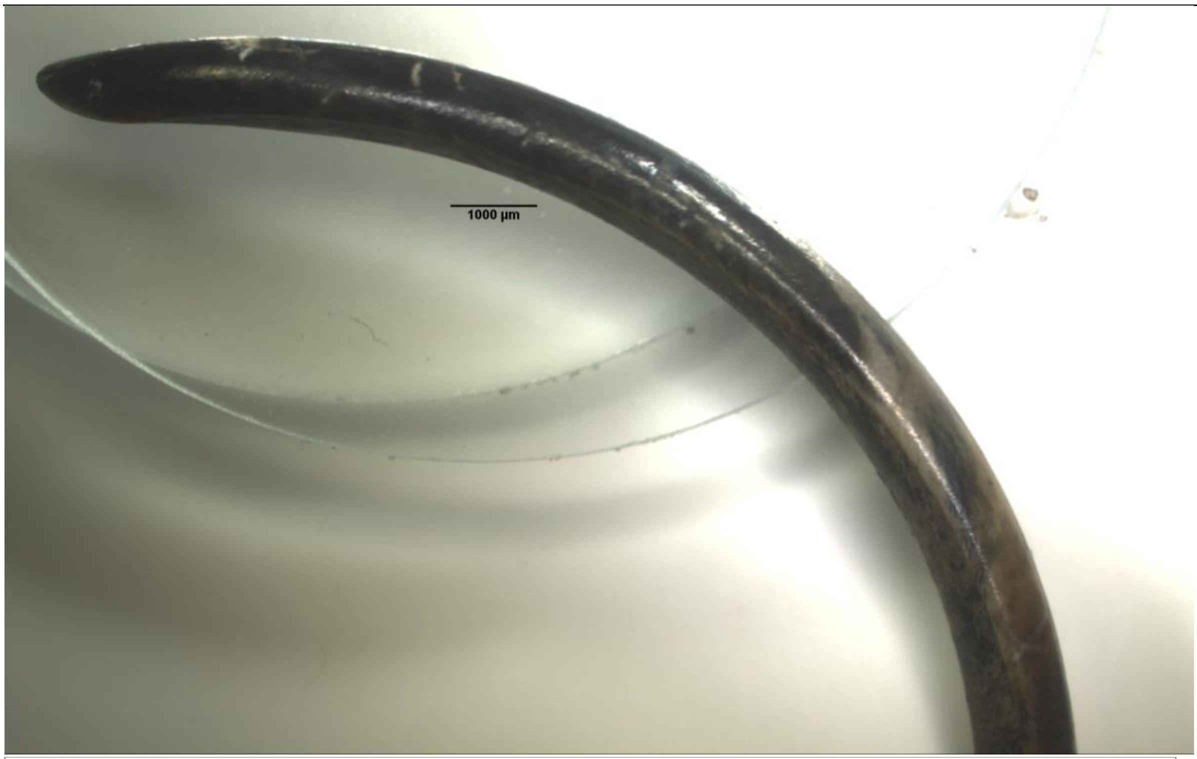

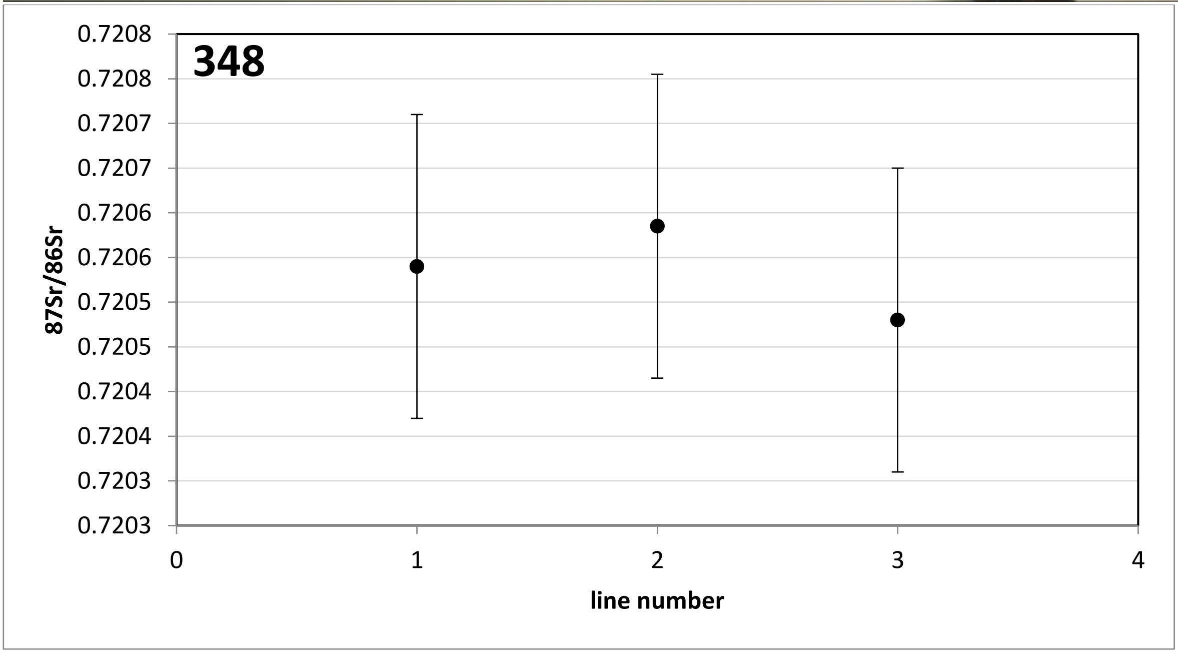

Fig S 29. Close up photograph of ablated Water vole 348 tooth (upper) and unique mobility pattern (lower). All black data points indicate 'enamel only' ablations. Photo: Adam Boethius

Table S 37. $^{87}Sr/^{86}Sr$ measurements for Water vole 349. Mean $^{84}Sr/^{86}Sr$ is 0.0564±0.0003 (2SD), with accepted value at 0.0565 (Thirlwall, 1991). [1]Propagated from external reproducibility (2SD) obtained from the primary standard during the analytical session combined with the within-run precision of each analysis (2SE) (Iolite Version 2.5). [2] average of total Sr beam intensity in V (sum of ^{84}Sr, ^{86}Sr, ^{87}Sr, ^{88}Sr). [3] estimated from measured Sr intensity in secondary RM using the same ablation conditions (semi-quantitative).

Line number	Sampling time (sec)	$^{87}Sr/^{86}Sr$	2SE	2SD[1]	$^{87}Rb/^{86}Sr$	2SE	$^{174}Yb^{2+}/^{86}Sr$	2SE	Mean Sr-Beam (V)[2]	Estimated Sr concentration (ppm)[3]	$^{84}Sr/^{86}Sr$	2SE	Data points / line
1	46	0.71982	0.00008	0.00018	0.00456	0.00018	0.00030	0.00005	3.9	222	0.05637	0.00013	91
2	54	0.71979	0.00010	0.00018	0.00305	0.00016	0.00031	0.00004	4.1	231	0.05650	0.00012	108
3	44	0.72031	0.00009	0.00018	0.00294	0.00018	0.00029	0.00005	4.2	236	0.05625	0.00012	87

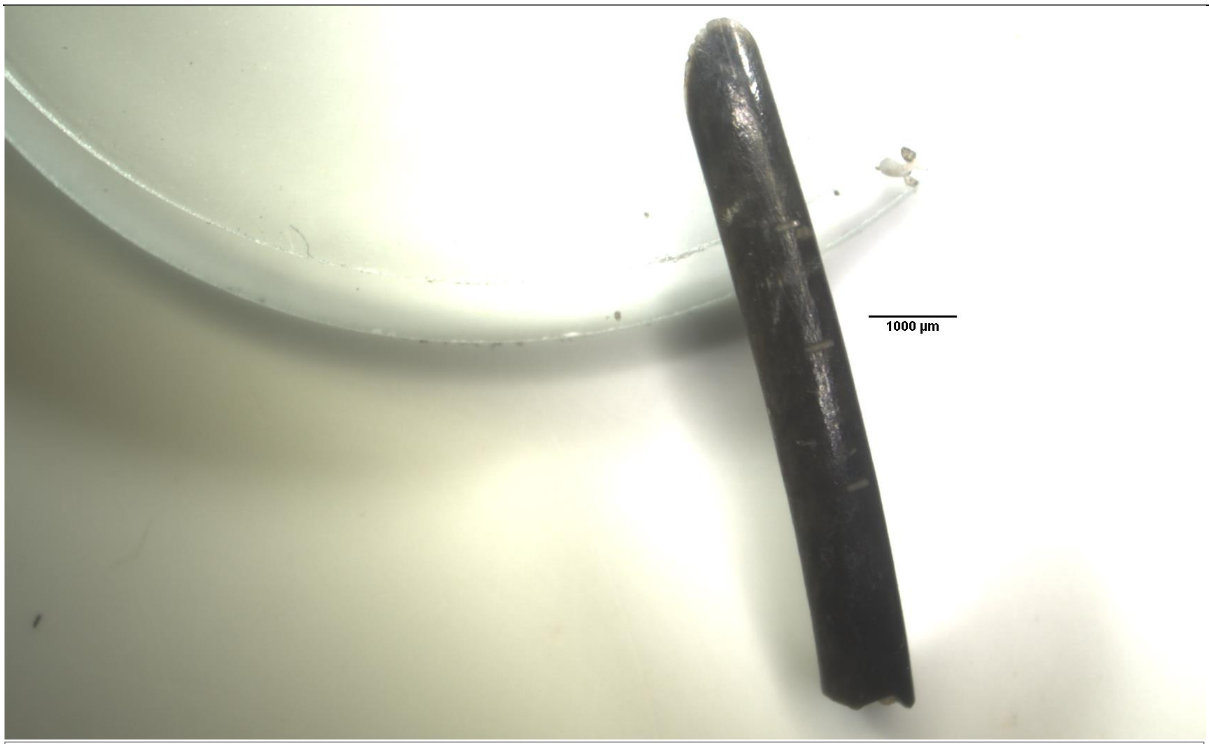

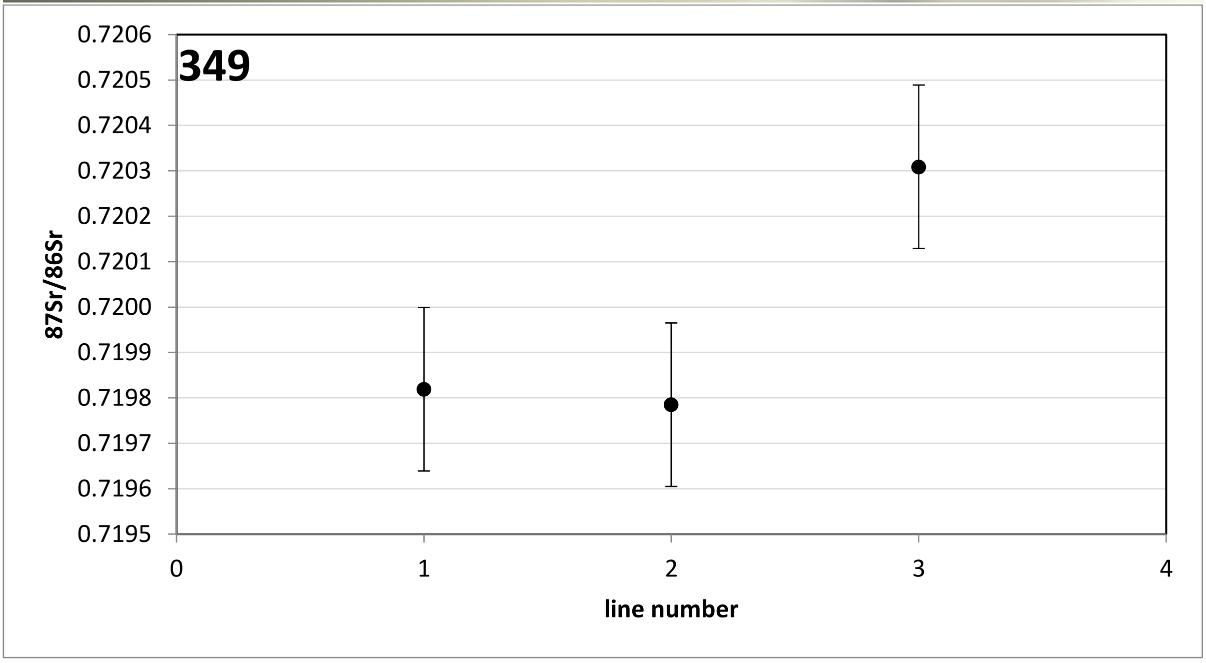

Fig S 30. Close up photograph of ablated Water vole 349 tooth (upper) and unique mobility pattern (lower). All black data points indicate 'enamel only' ablations. Photo: Adam Boethius

Table S 38. $^{87}Sr/^{86}Sr$ measurements for Water vole 350. Black coloured $^{87}Sr/^{86}Sr$ data marks 'enamel only' ablations as inferred from ocular examination of the close-up photograph and/or through changes in $^{87}Rb/^{86}Sr$ ratios. Mean $^{84}Sr/^{86}Sr$ for Water vole 350 is 0.0565±0.0004 (2SD), with accepted value at 0.0565 (Thirlwall, 1991). [1]Propagated from external reproducibility (2SD) obtained from the primary standard during the analytical session combined with the within-run precision of each analysis (2SE) (Iolite Version 2.5). [2] average of total Sr beam intensity in V (sum of ^{84}Sr, ^{86}Sr, ^{87}Sr, ^{88}Sr). [3] estimated from measured Sr intensity in secondary RM using the same ablation conditions (semi-quantitative).

Line number	Sampling time (sec)	$^{87}Sr/^{86}Sr$	2SE	2SD[1]	$^{87}Rb/^{86}Sr$	2SE	$^{174}Yb^{2+}/^{86}Sr$	2SE	Mean Sr-Beam (V)[2]	Estimated Sr concentration (ppm)[3]	$^{84}Sr/^{86}Sr$	2SE	Data points / line
1	44	0.71838	0.00017	0.00023	0.00094	0.00005	0.00014	0.00004	5.3	300	0.05638	0.00011	87
2	52	0.71978	0.00011	0.00019	0.00083	0.00004	0.00019	0.00005	3.9	220	0.05673	0.00013	103
3	52	0.71978	0.00007	0.00017	0.00078	0.00002	0.00024	0.00004	4.4	250	0.05648	0.00012	103

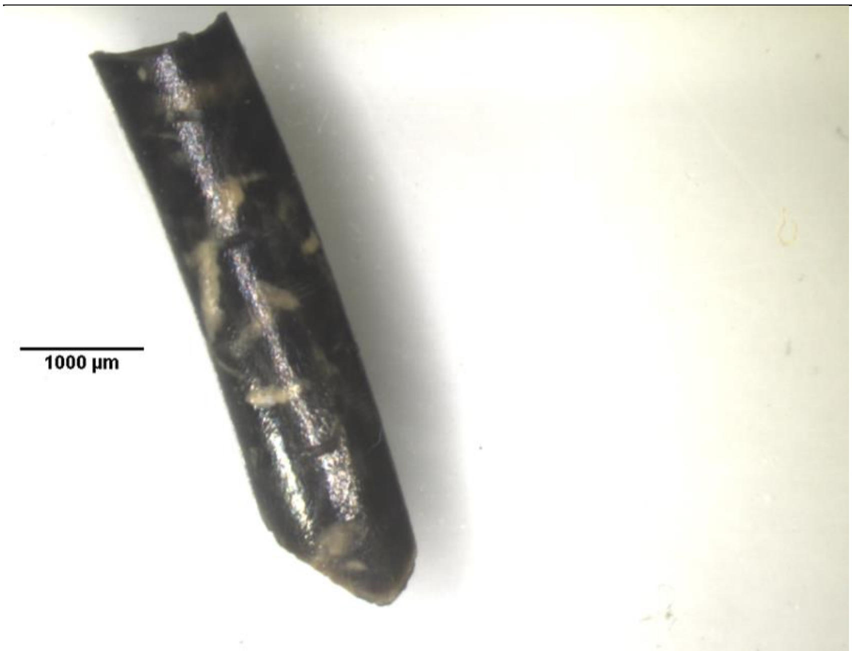

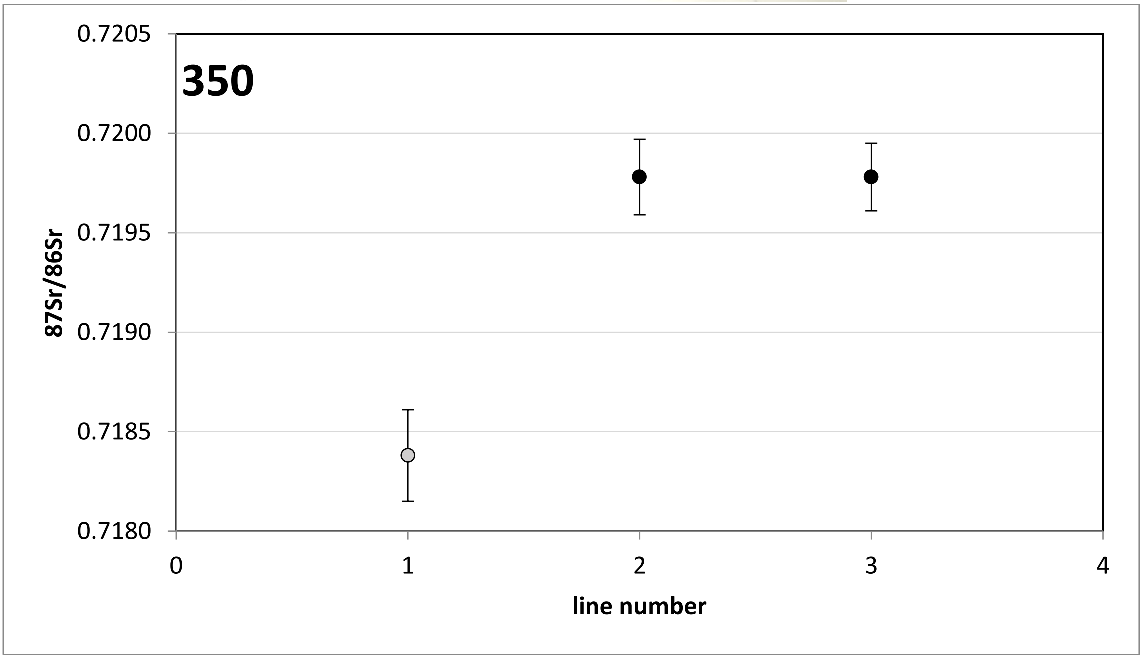

Fig S 31. Close up photograph of ablated Water vole 350 tooth (upper) and unique mobility pattern (lower). Black data points indicate 'enamel only' ablation; grey data point indicates enamel-dentine mix. Photo: Adam Boethius

Table S 39. $^{87}Sr/^{86}Sr$ measurements for Water vole 351. Mean $^{84}Sr/^{86}Sr$ is 0.0564±0.0002 (2SD), with accepted value at 0.0565 (Thirlwall, 1991). [1]Propagated from external reproducibility (2SD) obtained from the primary standard during the analytical session combined with the within-run precision of each analysis (2SE) (Iolite Version 2.5). [2] average of total Sr beam intensity in V (sum of ^{84}Sr, ^{86}Sr, ^{87}Sr, ^{88}Sr). [3] estimated from measured Sr intensity in secondary RM using the same ablation conditions (semi-quantitative).

Line number	Sampling time (sec)	$^{87}Sr/^{86}Sr$	2SE	2SD[1]	$^{87}Rb/^{86}Sr$	2SE	$^{174}Yb^{2+}/^{86}Sr$	2SE	Mean Sr-Beam (V)[2]	Estimated Sr concentration (ppm)[3]	$^{84}Sr/^{86}Sr$	2SE	Data points / line
1	46	0.71982	0.00008	0.00017	0.00065	0.00002	0.00058	0.00004	4.6	262	0.05640	0.00012	92
2	48	0.71985	0.00008	0.00017	0.00061	0.00003	0.00060	0.00004	4.5	253	0.05636	0.00012	96
3	41	0.72024	0.00006	0.00017	0.00120	0.00007	0.00038	0.00004	4.9	276	0.05656	0.00011	82

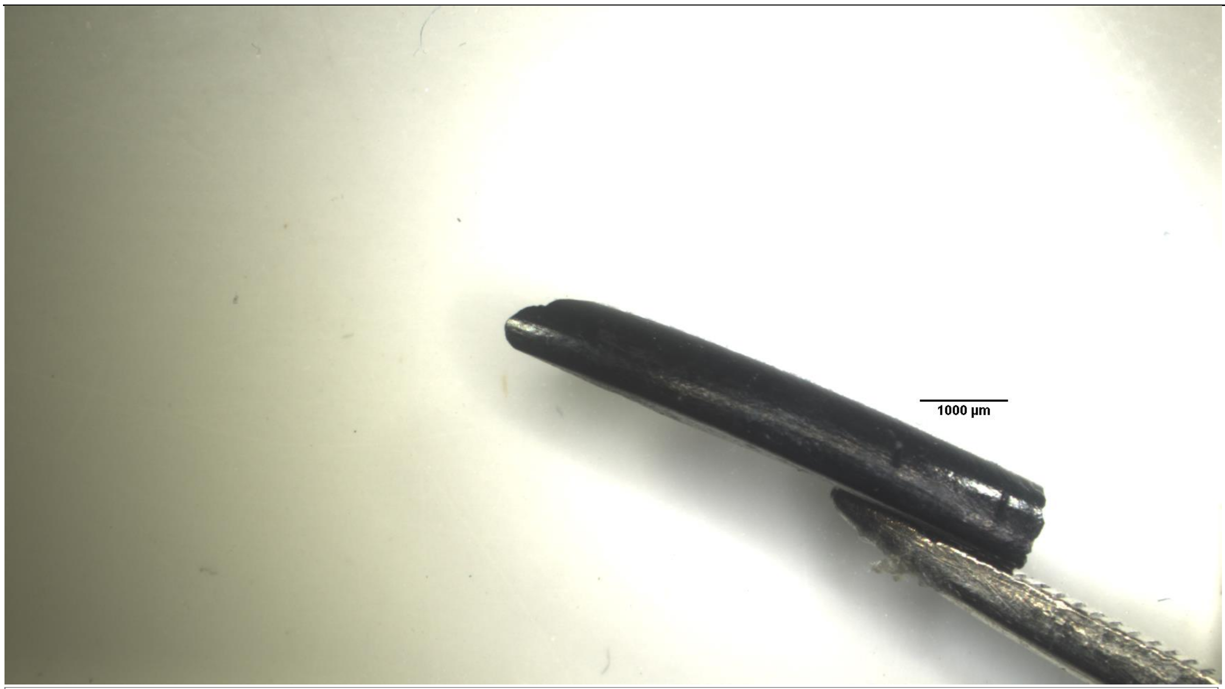

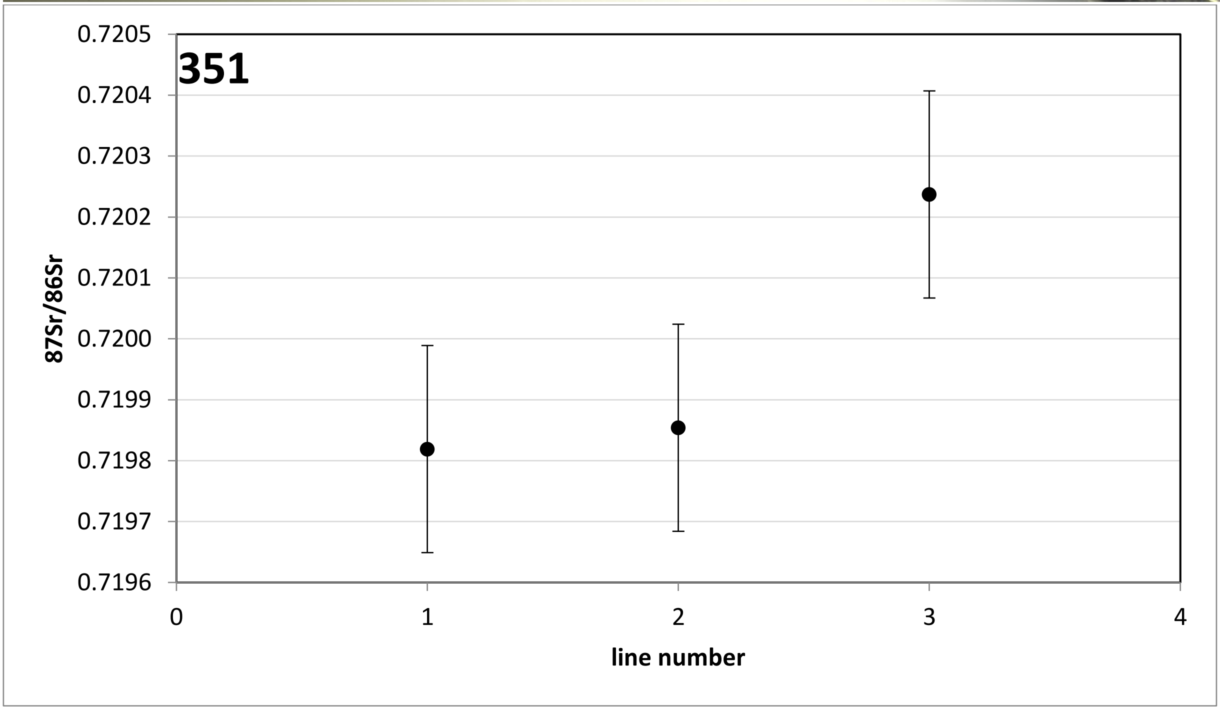

Fig S 32. Close up photograph of ablated Water vole 351 tooth (upper) and unique mobility pattern (lower). All black data points indicate ‘enamel only’ ablations. Photo: Adam Boethius

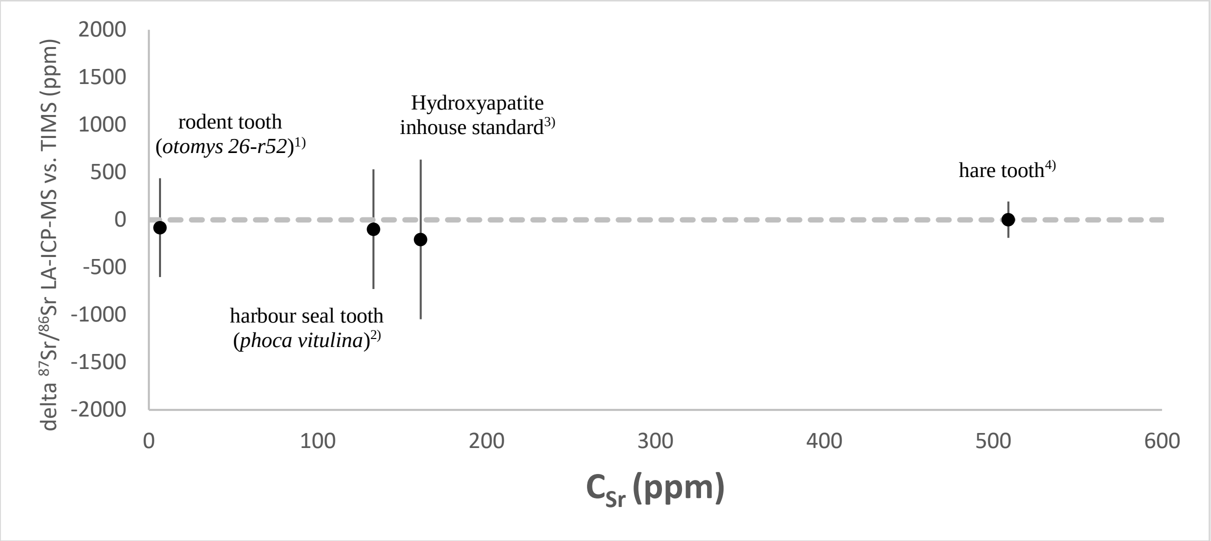

Fig S 33. Accuracy of LA-ICP-MS results in relation to Sr concentration in the sample. Demonstrating high accuracy even at low concentrations; TIMS $^{87}Sr/^{86}Sr$ and concentration data obtained on a Thermo Scientific TRITON TIMS at the Swedish Museum of Natural History, Stockholm, dissolved fraction of the teeth, purified by ion exchange; LA-ICP-MS data from the Vegacenter at the Swedish Museum of Natural History, Stockholm, obtained at optimal low-oxide tuning conditions, all corrections applied, average of 10-50 analyses on the enamel surface; error bars represent 2SD of LA-ICP-MS analyses; 1) rodent tooth (otomys 26-r52), C_{Sr} = 6.6 ppm (Le Roux et al. 2014); 2) harbour seal tooth (Phoca vitulina), C_{Sr} = 133 ppm; 3) hydroxyapatite crystal (Holly Springs), C_{Sr} = 161 ppm; 4) European hare tooth (Lepus europaeus) (Blåbergsholmen, Swedish Westcoast), C_{Sr} = 509 ppm

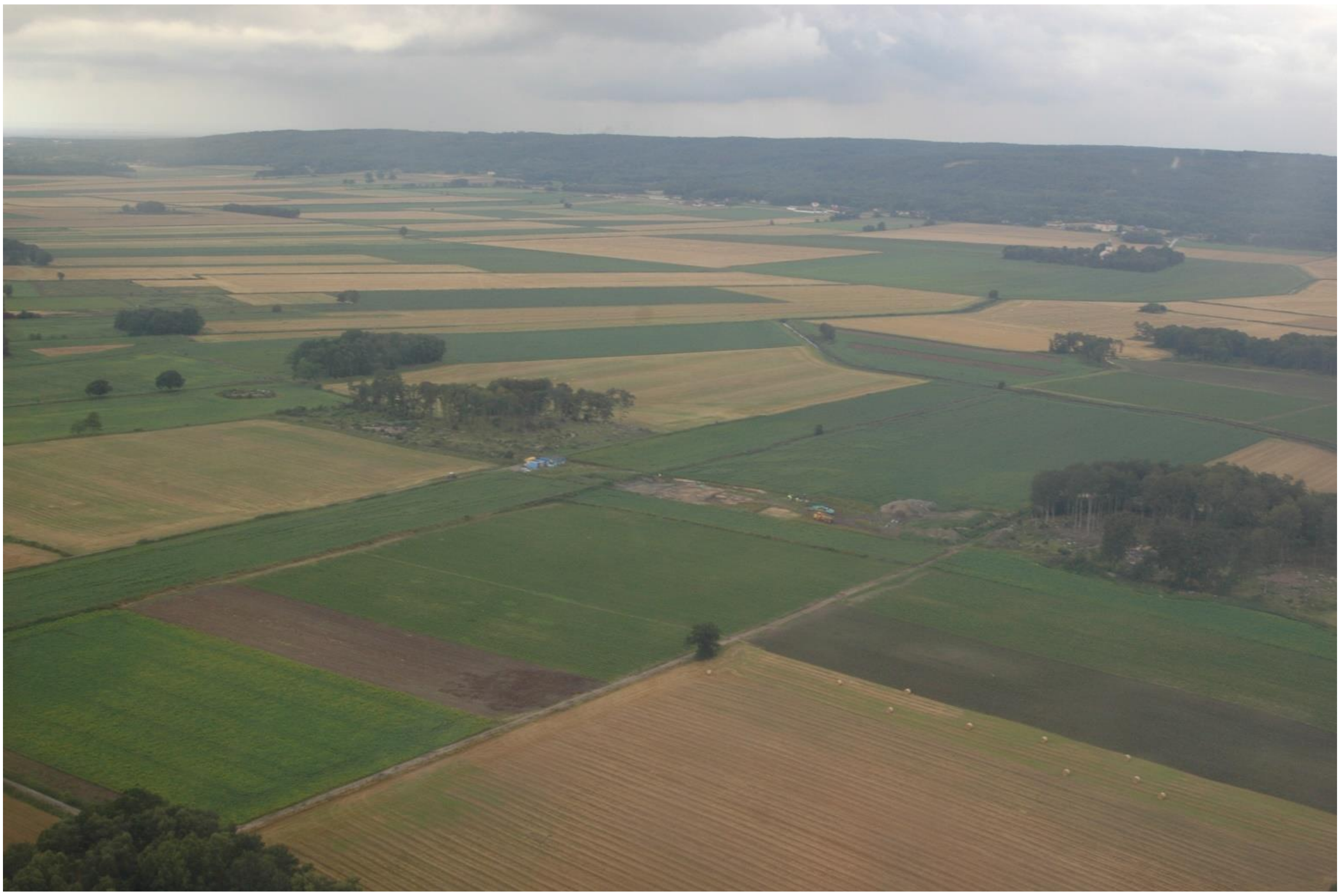

Fig S 34. Aerial photograph showing the site Norje Sunnansund during excavation and its location within the former lake. The whole former lake Vesan area have been drained and turned into farm land. Photo by Blekinge Museum.

Table S 40. Water analyses conducted at TIMS. Analyses conducted at the Museum of Natural History, Stockholm, Sweden, to establish and delimit the bioavailable $^{87}Sr/^{86}Sr$ ratio signals to the north and east of Norje Sunnansund. * Normalized to a NBS987 $^{87}Sr/^{86}Sr$ ratio of 0.710248. ** CIT#39 is an "in house" sea water standard. World average sea water normalized to NBS987 value above gives $^{87}Sr/^{86}Sr$ 0.709171 according to (Mokadem et al., 2015).

Month of analysis	LIG#	Water sample	$^{87}Sr/^{86}Sr$ spiked	Int. prec. 2σm	$^{85}Rb/^{86}Sr$	C_{Sr} ppm	$^{87}Sr/^{86}Sr$ normalized *	Ext. prec. 2σ
August	20103	I. Hålabäck	0.7205742	0.0000036	0.000001	0.015	0.720611	0.000013
	20104	II. Barnakälla	0.7126496	0.0000037	0.000001	0.162	0.712687	0.000013
	20105	III. Vitavatten	0.7210762	0.0000047	0.000001	0.027	0.721113	0.000013
	-**	CIT#39	0.7091377	0.0000039	0.000001	7.76	0.709175	0.000013
October	20178	IV. Skönabäckskällan	0.7130965	0.0000060	0.0564826	0.000012	0.713100	0.000013
	20179	V. Stora Svartsjön	0.7200250	0.0000039	0.0564832	0.000001	0.720029	0.000013
	20180	VI. Bredagylet	0.7190124	0.0000035	0.0564827	0.000001	0.719016	0.000013
	-**	CIT#39	0.7091780	0.0000043	0.0564852	0.000001	0.709182	0.000013

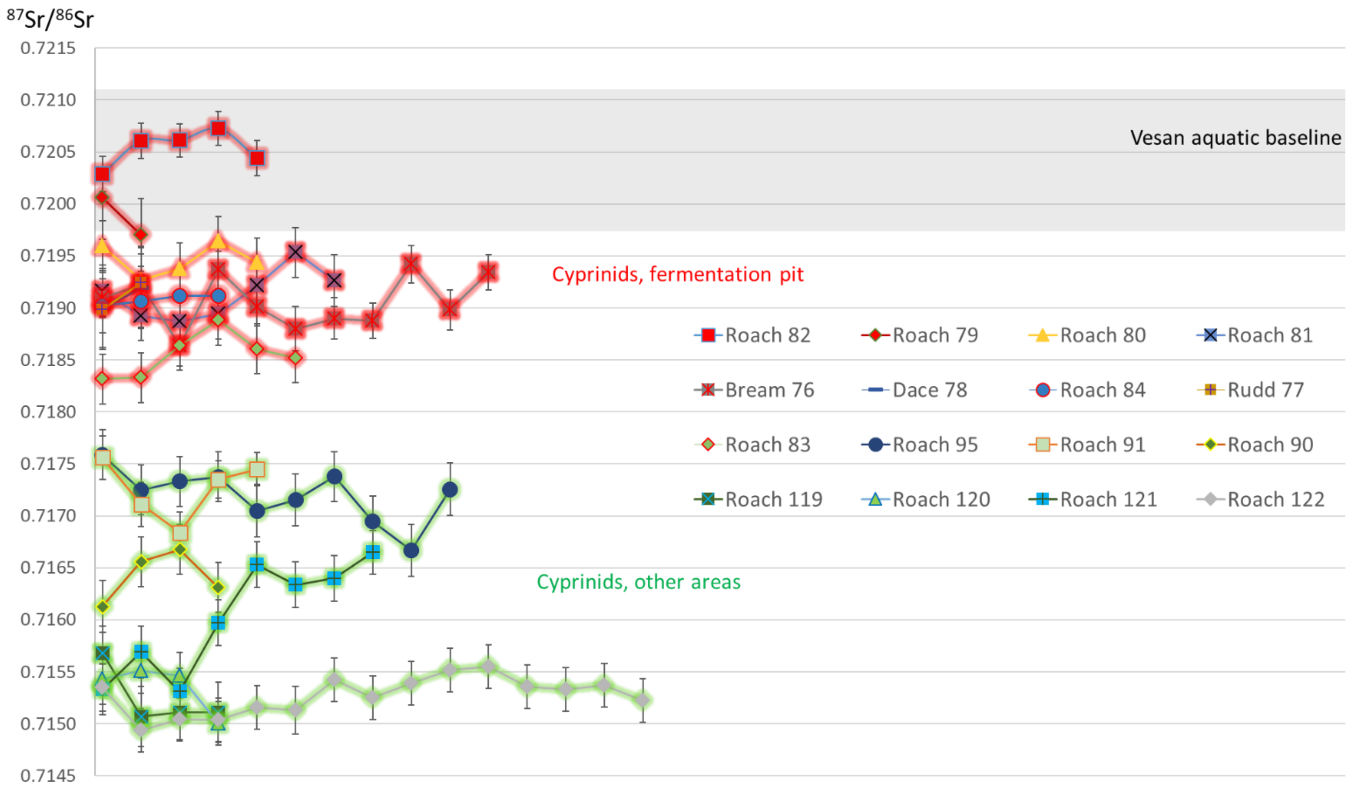

Fig S 35. Sr measurements of cyprinid teeth from Norje Sunnansund. Red represents cyprinids from within the fermentation pit; green represents cyprinids from outside of the pit. The grey rectangle shows the local aquatic baseline.

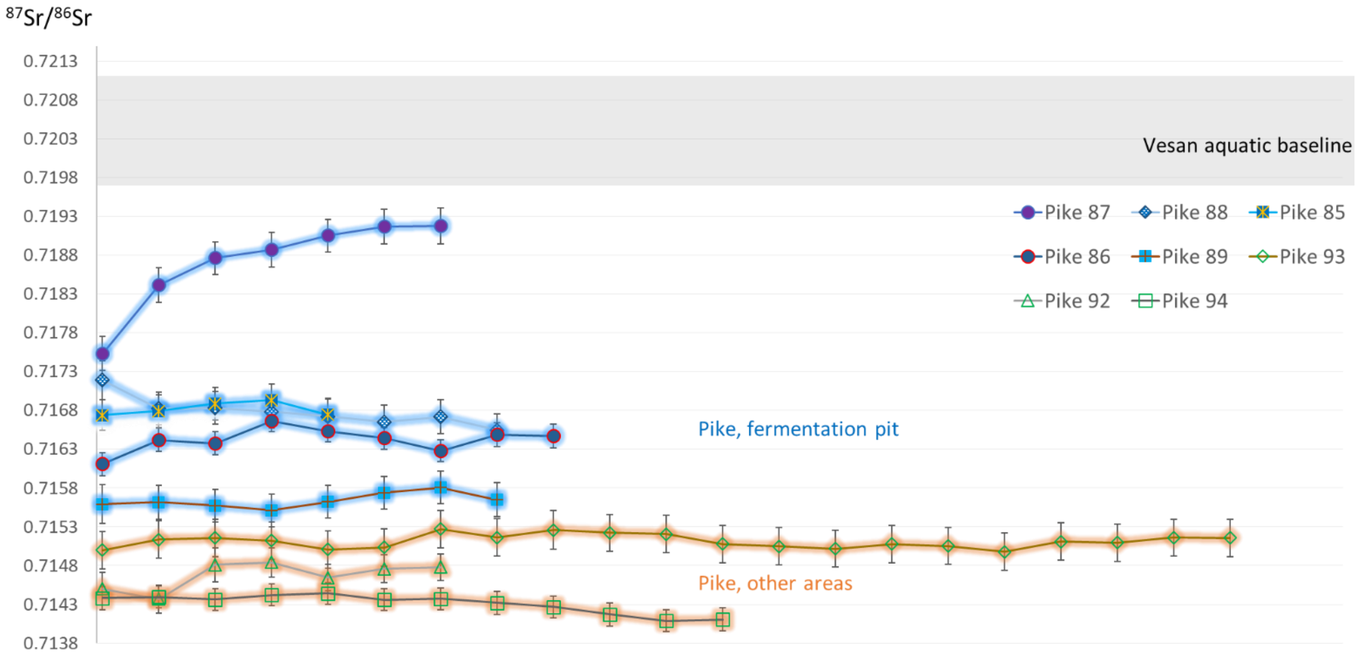

Fig S 36. Sr measurements from pike teeth from Norje Sunnansund. Blue indicates pike found within the fermentation pit; orange indicates pike found outside the pit. The grey rectangle indicates the local aquatic baseline.

References

Kjällquist, M., Boethius, A. & Emilsson, A. 2016. Norje Sunnansund. *Boplatslämningar från tidigmesolitikum och järnålder. Särskild arkeologisk undersökning 2011 och arkeologisk förundersökning 2011 och 2012, Ysane socken, Sölvesborgs kommun i Blekinge län*, Karlskrona: Blekinge museum.

Le Roux, P. J., Lee-Thorp, J. A., Copeland, S. R., Sponheimer, M., & De Ruiter, D. J. 2014. Strontium isotope analysis of curved tooth enamel surfaces by laser-ablation multi-collector ICP-MS. *Palaeogeography, Palaeoclimatology, Palaeoecology*, *416*, 142-149.

Mokadem. F., Parkinson. I. J., Hathorne. E. C., Anand. P., Allen. J. T. & Burton. K. W. 2015. High-precision radiogenic strontium isotope measurements of the modern and glacial ocean: Limits on glacial–interglacial variations in continental weathering. *Earth and Planetary Science Letters*. 415. 111-120.

Thirlwall. M. 1991. Long-term reproducibility of multicollector Sr and Nd isotope ratio analysis. *Chemical Geology: Isotope Geoscience section*. 94. 85-104.
