# Supplementary material for: Early Holocene Scandinavian foragers on a journey to affluence: Mesolithic fish exploitation, seasonal abundance and storage investigated through strontium isotope ratios by laser ablation (LA‐MC-ICP‐MS)
Source: PLoS One. 2021 Jan 20;16(1):e0245222. doi: 10.1371/journal.pone.0245222 (PMC7817046; doi:10.1371/journal.pone.0245222)
Supplement: Attachment — Submitted filename: Response to reviewers.docx [file pone.0245222.s002.docx]

Dear reviewers, thank you for your insightful comments. Below are our replies to your comments, the changes can be found in the updated manuscript.

Reviewer #1: The manuscript titled “Early Holocene Scandinavian foragers on a journey to affluence: Mesolithic fish
exploitation, seasonal abundance and storage investigated through strontium isotope
ratios by laser ablation (LA‐MC-ICP‐MS)” by Boethius et al. describes the use of strontium isotope ratios to identify origin and migration patterns of fish from the archaeological site of Norje Sunnansund, southern Sweden. Overall, 16 cyprinid and 8 pike teeth, from within and from outside of fermentation pits were analyzed using laser-ablation techniques and the results interpreted to support the idea of an year-round fishery with intensified use and high catches during seasonal fish migrations.
Overall, the article is well written and well organized with clear sections outlining the research questions and providing the necessary background and methods. The analytical and statistical methods are sound, and the author have met all ethical standards during data collection.

However, I have some major concerns about the discussion and interpretation of the data that I would like to see addressed.
1) The authors need to decide if the trust the dentine “contaminated” values or not. In several parts of the manuscript the authors rightfully point to the many potential problems in interpreting the dentine data (interferences, Rb content, timing of formation, potential diagenesis,…) and based on this I would suggest to remove these data from the main manuscript and discussion and add them to the supplement. In addition, the uncertain proportion of enamel-dentine mixing make these data incredibly difficult to interpret if the timing of formation of these materials is different. It appears to me that the discussion is still very useful without the dentine data and would be more streamlined and in tune with the archaeology focus. This would change figures 6-11 which as currently formatted are not easy to interpret and change the text of the entire results section. This could focus on potential migration paths and origins of the fish using the known baseline data for Sweden and the Baltic Sea.

Enamel-dentine mix interpretations removed and the concerned figures and text updated throughout the manuscript

2) Local baseline. I don’t think the vole teeth are a good local baseline for fish teeth from a nearby lake and river? I take it the lake has been drained and the river is not accessible anymore? Since a lot of the paper discussion hinges on local vs seasonal migration you will need to establish local values more robustly. Maybe based on the geology of the proposed watershed? Based on known local fish bones? It’s tricky but how would vole teeth be appropriate for a watershed and with only three samples with all different values who is to say the local range is not much larger? Also mean +-sd from 3 samples is not very meaningful. All this needs to be better established and supported by baseline data for the discussion to be more robust.

Agreed it was not good at all. It is very difficult to establish a good baseline for the area due to diverse geology and drainage. The whole former lake has been drained and is now agricultural land which has been repeatedly limed and fertilized over the last century. Furthermore, most of the close by lakes have also been limed and cannot be used to establish any accurate baseline. To remedy this we have created a whole new section in the paper dedicated to establish the aquatic baseline. We have sampled water sources and water voles (from which we also incorporated the diet) to estimate the most likely aquatic baseline of the former a lake) We have updated the manuscript following this new data.

3) If the fish migrated as suggested in figure 12 than why do you not see any Baltic Sea values in the enamel samples?

We do not suspect the fish to originate from the Baltic Sea itself but rather the coastal areas to the east and north of the site for cyprinids and for pike either the archipelago just outside Baltic coast of the site or the “Hanö-bay” region to the south. The whole figure has been remade to better show what we mean and text have been updated at appropriate places.

Minor comments
4) Section - Prerequisites for studying provenance and mobility using strontium isotope ratios. Since this paper combines ecological and archaeological interpretations I would expect a short introduction to Sr isotopes in fishery research here so citing some studies using otoliths. How are isoscapes for these types of studies normally constructed and what kind of information can be gained? Sean Brennan’s work on building robust water isoscapes for pacific salmon could be cited here in addition to more regional papers focusing the Baltic (e.g., Glykou et al., 2018).

Added sentences and references: For more information of how strontium is incorporated into a body see e.g. [75] and for further information of bioavailable strontium and discussions of its use in archaeological research see e.g. [76-78]. For information of its implementation in fish ecological research (primarily done on otoliths) and how fish migrations and mobility is integrated with environmental isotopic variations across space and time, i.e. isoscapes [79, 80], see e.g. [81-84].

5) How did you ensure that each tooth (from the same fish species) represents a unique fish?

Added following argument: … and it limited the likelihood of the same individual being sampled more than once. The risk of double sampling was also deemed unlikely due to the very large number of recovered fish teeth from a large number of identified fish individuals and based on estimations suggesting the possibility of hundreds of tons of originally caught fish at the site [66].

6) Section - Fish tooth development. Could you expand on your best assessment here what time frame these teeth capture? Is it Months? Years? Are there any morphometric features that can provide insights into this? Added sentences: The exact duration and timing of pike teeth development has not been thoroughly researched. However, pike has, because the adult dentition is characterized by numerous teeth of different size and shape throughout the oropharyngeal region, recently been identified as “a powerful model system for studying tooth development” [91]; whereby, more exact data on fish tooth development can hopefully be produced over the coming years.

Just a comment here that the supplementary materials are excellent! This should be required more often and its great to see the authors provide all these details!

Line 26-29: Not clear to me what “the mobility signal of the fish is specific and follows general patterns” means. Changed to:. By applying advanced strontium isotope analyses to archaeological material integrated into an ecological setting, we present a methodology that can be used elsewhere to enhance our understanding of the otherwise elusive indications of storage practices and fish exploitation patterns among ancient foraging societies.

Line 89: Maybe provide more examples instead of “etc.” Changed to: as well as modern land exploitation and more intense land development in certain areas, in combination with a somewhat stronger research tradition in Mesolithic archaeology than in other parts of the world.

Line 107: To clarify today the site is not near the river and the lake has been drained? Some more information about the site today vs before would be useful for a not familiar reader. Added more information about the site in the introduction (yes the lake has been completely drained and the area have been extensively limed).

Line 280-285: Add the number of standards run and what the error represents to the text. (e.g. n=, +-2sd). Changed in text, it now reads: The analyses on the shark spine yielded a ^87^Sr/^86^Sr ratio of 070918 ± 0.00028 (2SD, n = 204, November 2019), 0.70919 ± 0.00032 (2SD, n = 291, February 2020), 0.70918 ± 0.00036 (2SD, n=19, September 2020) and 0.70918 ± 0.00034 (2SD, n=33, October 2020) which agrees well with the value of seawater of 0.709179 ± 0.000002 as established by [98]. The hare tooth yielded a ^87^Sr/^86^Sr ratio of 0.71009 ± 0.00026 (2SD, n = 125, November 2019), 0.71009 ± 0.00027 (2SD, n = 68, February 2020), 0.70991 ± 0.00020 (2SD, n = 4, September 2020) and 0.70999 ± 0.00030 (2SD, n = 7, October 2020). This agrees well with the value determined by thermal ionization mass spectrometry (0.709988 ± 0.000015; Finnigan Triton, Thermo Scientific, Waltham, MA, USA) at the Swedish Museum of Natural History.

Figures general: I strongly suggest to redo these figures in R or python (or any other stat plotting software), especially the boxplots would be much nicer using one of those software packages. Also avoid red and green together. The figures looks good if you download the complete images as .tif, but yes they look bad in the created pdf. Please download the complete image using the inserted link in the manuscript text

Figure 1: I suggest swapping the maps around. The regional map showing the site, river, and lake as the main part of the figure and a small insert showing the location in Sweden. It would be very useful to add other major rivers and lakes to this. Figure updated

Figure 5: Need to add the Baltic sea values to this maybe from Glykou et al., 2018:figure 1? We moved the dot a bit more to the North-East so it would be easier to spot and added “Baltic Sea” in the map.

Figure 6: Sort by species and fermentation rather than by sample number? Figure updated

In summary this paper represents a significant contribution to combining archaeological and ecological research and I expect it will be of great interest to a wide scientific audience once the mentioned concerns are addressed but as it currently stands I suggest it needs major revisions.

Reviewer #2: I am sorry to say that as the data is currently presented, I am not sure I trust the results and would be wary of citing the paper. As the paper is currently presented, the use of dentine data mixed with enamel data here is concerning, and unless much stronger justification can be given, I suggest that all the dentine data and mixed enamel-dentine data should be removed and the interpretations re-written based solely on the enamel-only data. My reasons for thinking this are as follows:

Only enamel data now included in the text. All dentine influenced data points discarded

1. Some of the dentine-contaminated tooth profiles move towards the bioavailable range quoted for the site, but not in every case. This is potentially interesting, but only if the stated baseline bioavailable range is fully representative for the site and the burial contexts. Therefore, more information is needed on how the bioavailable 87Sr/86Sr baseline range for the site was established. Currently, a range is given (0.7166 to 0.7180) with a reference to three rodent teeth, but is this enamel/dentine/whole tooth, where specifically were the teeth found on the site, and are they archaeological or modern, burrowing rodents or not? Also, what is the site stratigraphy and could the “fermentation pit” have been dug through different sedimentary horizons/geological substrates, with different bioavailable ranges? Possibly this information is in some of the cited references but it needs to be given here, at least in summary form as identifying the local bioavailable range correctly is critical to the interpretations (see next comments).

Added new sections to the introduction of the Norje Sunnansund to give more contextual information regarding the site. Reworked the baseline using new water samples and water vole teeth and updated the text throughout the manuscript

2. Dentine is porous and typically equilibrates with the burial environment – this fact is used in many studies to compare enamel and dentine values to identify migrants. Enamel-dentine comparisons are also common when studying large teeth (cows, sheep etc.) with much thicker enamel than the fish teeth reported here, precisely because the dentine equilibrates. The argument that dentine from tiny fish teeth, with very thin enamel, can be preserved without contamination, needs much stronger justification here if the dentine results are going to be treated as giving an original uncontaminated signal.

We have removed all enamel-dentine data from the interpretations and kept only enamel data within main text and have reworked the manuscript accordingly

3. Dentine-only data that falls outside the local bioavailable range would be a good indication that the dentine is preserving something meaningful. But at the moment, the data is presented only as enamel, and enamel-dentine mixed, meaning it is not possible to know if this is the case. The images of the sampled teeth suggest that there should be some dentine-only data, and this needs to be collated and analysed specifically in its own right.

Too uncertain to rely on, we have removed all data including dentine from the interpretations

4. Importantly, if the dentine is contaminated with Sr from the burial environment, data from mixed enamel-dentine samples is essentially meaningless as it is impossible to know how much the contamination has affected the results, and it should be removed entirely from the dataset. we have removed all data including dentine from the interpretations

Aside from the issues with the dentine data, I also think a comment needs to be inserted justifying the notion that the Sr ratio in the very thin enamel is itself unaffected by diagenetic contamination. In my experience it is common for the outer few 10s or 100s of microns of tooth enamel to be contaminated on every enamel surface (including at the enamel-dentine junction). This goes for teeth I have analysed by laser ablation from species with both large teeth with thick enamel and small teeth with thin enamel. Given the enamel in the fish teeth is so thin, it would theoretically be quite easy for the contamination to permeate the entire tooth, including both enamel and dentine. The spread in enamel-only values is good evidence that the enamel is uncontaminated, but some acknowledgement that this potential issue has been thought about is needed.

*It is indeed difficult to argue that the enamel has not been affected by contamination. The preablation remove a few microns, but not much more. We argue, that we don’t observe increased amounts of REEs or Rb in the fish teeth samples, which would hint towards contamination. We added following sentences to clarify our position:* Enamel can also be contaminated by its depositional soil environment. To ensure that the enamel of the teeth was not subjected to soil contamination we preablated each ablation area (thus discarding the outer few µm of the enamel, cf. instrumental settings Tables S1-4). The results indicate that we do not see any increases in either rare earth elements (REE) or Rb, compared to the standards, which would indicate contamination. The spread and variation of the fish enamel measurements offer further indications that the enamel has not been contaminated by its depositional soil environment.

The Supp info gives some summary information for each line sampled in each tooth, but this is not sufficient to judge the data. Specifically, the tables give “total Sr beam” in volts for each line measured in the teeth, but I could not find an explanation of what this represents (masses 84-88 of all integrations??) and it is not useful for assessing the data because it relates mainly to the length of the line and not the data measured. Rather, please modify these tables to show number of integrations / datapoints for each line to have a clearer idea of what the SE and SD relate to (n); and also a MEAN 88Sr (not total), which might give a much better idea of when the concentration of Sr in the sample is increasing and the dentine/enamel are contaminated.

*“Total Sr beam” represents the average total Sr beam (sum of all Sr-masses 84-88). It should be called “mean Sr-beam” to be more clear, which has now been done. Also, data updated in supplementary by adding the categories: “174Yb2+/86Sr”, “estimated Sr concentration (ppm)” and “datapoints/line”*

Also on identifying contamination, 89Y is commonly used in laser ablation studies to monitor for rare earth element contamination (e.g. Wilmes et al. 2016, whom you cite), and can also be a useful indicator for the presence of diagenetic strontium – if high concentrations of REEs have leached into the enamel from the burial environment, there is every chance that elemental Sr has as well. Did you measure 89Y and if yes what does this show? If not, another possibility might be exploring Sr intensities in enamel-only and dentine-only data, and comparing these groups. This is something that should be included, at least in the Supp Info but probably needs a mention in the main article too.

*^89^Y could not be measured, because we do not have enough detectors available to detect additional masses. However we measured ^173^Yb^2+^, which could be detected on the un-interfered m/z 86.5. Natural abundances of Yb have been used to calculate the potential contribution of ^174^Yb^2+^ of m/z 87, and ^174^Yb^2+^/^86^Sr is a good indicator for potential contamination of the sample. We added this information to the data table in the updated supplementary file.*

Figure 6 – group the samples according to where they were found (inside or outside the fermentation pit). This would make it a lot easier to read. Figure updated

Figs 7 and 8 – it took me a long time to recognise what you meant by “shading”. Perhaps “thickened line” is a better description (this may be a result of how the graphs appeared on my screen). Probably, images need to be fully downloaded as .tif to look good. Also, since the dentine measurements are now removed there are no thin lines anymore.

Figure 12 needs a scale. Figure updated

Reviewer #3:

I am unfamiliar with the archaeology of this location and period so I will restrict my comments to the quality of the LA strontium isotopic data.
Unlike conventional solution Sr isotope analysis, where ion exchange chromatography is used to remove most of the elemental/molecular interferences, LA Sr isotopic analysis suffered from a whole range of potential interferences. The authors use standard procedures to mitigate most of these (REEs Rb K etc), though I have some concerns about whether then have demonstrated that they have eliminated the Ca/Ar PO interference. This interference (predominantly on mass 87) has the potential to cause a positive offset on measured 87Sr/86Sr of several 000s ppm which would be highly significant given the c. 6000 ppm range of the authors’ data. The magnitude of the interference will vary with Sr concentration in the analyte but not with sample throughput (e.g. from varying laser spot size or repetition rate).The author’s attempt to eliminate this by careful control of oxide formation, and claim that they have been successful through the measurement of two internal standards of a shark spine and a hare’s tooth which show negligible offset from their known Sr isotopic values. This is a standard approach, but only valid if the Sr concentrations of the standards is equal to or lower than the Sr concentrations of the samples. This has not been demonstrated in this paper. The issue is further confused by the different ablation conditions used for standards and samples, rending Sr voltages difficult to relate to concentrations, and there is some confusion what the Sr Voltages quoted actually represent. Are the Vs integrated over the whole run, or averaged; it is unclear why Sr V is reported as ‘total beam average’ for the standards and ‘total beam’ for the samples? If both are meant to be the former, then the difference in spot size would mean c. 3-4x greater voltage per Sr concentration for the samples than the standards. If the latter is the V integrated over the whole run then the difference is far greater than this.
The LA results may well be unaffected by a Ca/Ar PO interference, but the authors need to robustly demonstrate this before I would recommend publication. I would suggest the authors:
- Include details of the Sr concentrations of the standards

*We have information about the Sr concentration of the hare tooth. We included that information in the supplementary file. Ablation conditions were not different between standard and sample, only the spotsize was smaller for the shark spine, because of high Sr concentration in the material. The additional graph Fig. S33, demonstrates that the Ca/Ar PO interference is not a concern, even for low Sr concentrations.*

- Remeasure the standards and two or three of the samples under the same laser and MS conditions, to calibrate and calculate a semi quantitative Sr concentration for all samples. Note, this procedure does require re-measuring of both as variations over time in instrument sensitivity precludes an extrapolation of a new measurement solely of the standards (e.g. at 150 micron spot) to previous analyses.

*The secondary standard (hare tooth) has always been measured under the same conditions as the samples in regular and frequent intervals. Therefore we can go back to the data and calculate semi-quantitative concentrations for all samples. We included this data in the updated supplementary.*

- Demonstrate that the neglibile LA-TIMS/seawater offset measured in the standards will also be the case for samples as the standards have similar or lower Sr concentrations.

*We can include a graph (Fig. S33) in the supplementary file, that demonstrates that accuracy (<200ppm offset) can be achieved with these tuning conditions, even for low-concentration samples (demonstrated for concentrations down to 6.6ppm)*

The authors might also look at the variation in Sr isotopic values with Sr V where the voltage (and hence the Sr concentration) varies significantly. There does seem to be a downward trend in Sr isotopic values with increasing V for a couple of samples I looked at in detail (121 and 95). This might be attributed to the CaPO interference, though there are other potential explanations.

*This can easily be explained by isotope mixing of two or more sources with differing isotope ratios and Sr concentrations. It is very common to see a dependence between Sr concentration and isotope ratio. The fact that accurate results can be achieved in very low concentrated samples (as demonstrated in Fig. S33) is a good indicator, that the CaPO interference is not influencing the results.*
