# Supplementary material for: Early Holocene Scandinavian foragers on a journey to affluence: Mesolithic fish exploitation, seasonal abundance and storage investigated through strontium isotope ratios by laser ablation (LA‐MC-ICP‐MS)
Source: PLoS One. 2021 Jan 20;16(1):e0245222. doi: 10.1371/journal.pone.0245222 (PMC7817046; doi:10.1371/journal.pone.0245222)
Supplement: Attachment — Submitted filename: Response to Reviewers.docx [file pone.0245222.s003.docx]

6. Review Comments to the Author

Please use the space provided to explain your answers to the questions above. You may also include additional comments for the author, including concerns about dual publication, research ethics, or publication ethics. (Please upload your review as an attachment if it exceeds 20,000 characters)

Reviewer #1: Comments to the Author
The authors have responded to the comments by all three reviewer’s and it is clear that a lot of work has gone into these revisions. Specifically, the main problem of the dentine values seems to have been taken care off. However, I still have additional comments that I would like to see addressed before the manuscript can be accepted.

Title: “Early Holocene Scandinavian foragers on a journey to affluence: Mesolithic fish
exploitation, seasonal abundance and storage investigated through strontium isotope
ratios by laser ablation (LA‐MC-ICP‐MS)”. Rereading the manuscript I don’t see the direct connection or measurements of “affluence”, or quantitative estimates on “abundance”. I suggest rethinking the title with the final manuscript in mind. Its really about fish exploitation using seasonal migratory species. We disagree. The whole point of the paper is to show that Early Holocene foragers were able to optimally use fish migrations to become “affluent”. Indeed this is also the final conclusion of the paper (the second last sentence in the concluding remark section). Furthermore, while we are note quantifying anything in the paper what we are showing is that they were using seasonal abundance, i.e. they were “harvesting” the fish as they were agglomerating for spawning or migration, hence the abundance. We argue that investigating the possibility for foragers to become affluent is connected to seasonal fish abundance and as such constitute the pillars of this paper. For these reasons we consider the title appropriate.

Major comments
- The results section (baseline data) includes a discussion of the results and provides context. I suggest splitting this into results and discussion and only present the new data obtained in this study in the results. Make everything baseline related a single result section and compile the new baseline Sr isotope ratios into a table (maybe adding lat/lon?, or other geographic identifiers). Here the fundamental differences between our intention with the paper and the reviewers approach become pronounced. Had this, first and foremost, been intended as a methodological paper we would certainly agree that the discussion taking place in regard to the baseline data should be moved to the Discussion section. However, we intend this paper to be an interpretative paper focusing on Mesolithic foraging societies and how they interacted with their environment. Thus our discussion section is mainly aimed towards these interpretations. To write this paper we had to develop a new methodology for acquiring an aquatic baseline when no water is available and therefore we had to contextualize and show how we developed a way to estimate this baseline. If we were to move this contextualization to the discussion section our presentation of the fish measurements would not make sense to the reader. The results of the fish ablation are dependent on the baseline rationalizations, discussion and contextualization to be fully understood and therefore we argue that the order of how we present the findings need to stay as it is. Thereby, and given our intention with the paper, we would argue that our way of contextualizing/motivating the sampling strategy and the aquatic baseline creation is indeed a result, although not a quantitative one.

- I suggest to combine Figure 5 and Figure 6. There is too much information in the figures and the captions, if we combine them we think there will be too much. We feel it is better to keep the two figures separated

- Figure 7 could be a point or a boxplot showing the Sr isotope ranges for all considered units (so published data + the new data from this study). We are not sure what the reviewer means by this comment. All of the data shown in the figure is from this study. There does not exist any prior data from plausible aquatic sources in the area. We have combined the boxplot and the scatter plot into one figure to better visualize the data and save space by not using two figures.

- What extra information do Figure 9 and 10 provide that is not conveyed in Figure 8? Also double check the colors between figures and legend and maybe avoid red and green together. Yes, well maybe figure 8 and 9 does not add much information. We included them because they add a different perspective, but considering the length of the paper, we now moved these two figures into the supplementary and saved some space. Also, the legends are accurate and we have added a black line surrounding the red dots in Fig. 8, this combined with the explanations within the figure and in the caption should ensure that everybody understands what is what.

- Figure 11, add jittered datapoints to these boxplots (maybe with a grey outline)

Figure redone and datapoints added

- The results of the fish teeth also mix results and discussion which should be clearly separated. If citing literature (other than data sources) it probably should be in the discussion section. We had only one reference in total in the result section and half a sentence which could be considered as belonging to the discussion section. This has now been deleted.

- How are the concluding remarks different than the discussion? I first thought of them like a summary but for that purpose that section is too long. Maybe streamline it and move some of the details into the discussion? They are very different. The discussion focus on the Sr analyses of the fish teeth and aim to show how the fish migrated and when. The concluding remarks, on the other hand, are intended to contextualize the ability, among a Mesolithic foraging society, to optimally use fish migrations and what it implies for the society in general. Both on a site specific level but also for our understanding of other contemporaneous hunter-fisher-gatherer societies. The concluding remark section should not be viewed as a summary.

At 12 figures + a large supplement the paper is very long when it really doesn’t need to be. The story is relatively simple (and very interesting!) but gets diluted by all the extra details that don’t contribute to the major research questions which if I understand correctly are simply (1) are the fish local (to the lake) or migratory and does this differ between the pit vs non-pit. I strongly suggest to remove anything that doesn’t directly relate to these questions. We don´t agree here. Yes, sure it is a very long paper. But a lot is methods and explanations why the choices have been made. However, the papers is not mainly a methodological paper, quite the opposite. It explores a fundamental need among foragers if they should be able to become sedentary or not. For this reason, the introduction of sedentsim among foragers is needed. We have gone through the text and removed some sentences that were perhaps not essential to the study, but we feel that the main bulk of the paper needs to be included.

Figures in general: Provide clear x and y axis labels (on the outside of the axis not in the plot) We removed the labels from within the plot and positioned them over the axis, report Sr isotopes to the same decimal value we now report 4 decimals in the figures and in the tables the exact data is shown with 6 decimals, and use a period and not a comma (so 0.70198 instead of 0,70198).DONE I again suggest to redo the figures in R, python, matlab, or similar. New figure 9 done in matlab?. This is a really interesting paper but the figures are a major drawback and could be easily improved through use of an alternative to excel software. Also consider color choices as a lot of people may have problems differentiating the different greens and reds. Though we kept the colour in fig 8, we have made sure all details in the figures are understandable without colour (different type of dots and/or explanation in the caption)

Minor comments
Abstract
Line 14: “We have analysed 87Sr/86Sr strontium isotope ratios..” reword to “We analysed strontium isotope ratios (87Sr/86Sr)…” Done

Line 17: “centres of origin”, do you mean “regions of origin”? Yes, changed to regions
Line 17:” Our results suggest”, replace with “We find” Done

Line 18: Not everyone will know that roach are a cyprinid. Maybe you can reword and say…”the most commonly fermented cyprinid, roach,..” and then in the next sentence add “This is in contrast to the other cyprinidis…” sentence changed to: Our results suggest that the most commonly fermented fish, cyprinids (roach)

Line 23: remove “show low, non-local, Sr ratios 23 and” you don’t give the Sr isotope ratios for the other fish Done

Line 167: replace “its” with “their”
Fixed

Line 170: Space missing between “Fig1Sand”

Fixed

Line 191:” Mobility and provenance studies is” it should be “are”
Fixed

Line 198: That’s not correct. The half-life doesn’t influence if the ratio would be altered when moving from the minerals in the rock to the soil and watershed. The long half-life just means that for archaeological studies no additional Rb decay needs to be accounted for.

Changed sentence to: Because the half time of ^87^Rb is very long (ca. 49 billion years) no additional Rb decay needs to be accounted for in archaeological studies. Furthermore, the 87Sr/86Sr ratio remain unaltered as it is transferred from the bedrock to the top-soil and further into water and plants in a particular area [71].

Line 205: Remove “of the bioavailable baseline”
Done

Line 260: Replace “over” with “for”
Done

Line 268 (and rest of this paragraph): Remove the “(see Results)”
see Results removed

Line 334: Maybe mention that enamel/dentine mix was excluded

Added: , whereby the last two ablations were excluded.

Line 345: I don’t think organics cause larger fractionations. But interferences makes sense Fractionations removed

Line 528: If you use the word significant you need to provide a statistical test. Maybe reword? Fixed, it now reads: Similarly, pike found within the pit was separated from pike found outside, indicating different origins for the different populations.

Line 533. Period missing Fixed

Line 558: The first paragraph should pickup where the introduction left off and summarize the results. The following paragraphs can then provide the detailed discussion of the results and the context. Lin 602 might be a good start for the discussion followed by :” We found…”

Our discussion section does start where the introduction leaves us, but with a focus on the investigated site instead of the method used. The two small initial paragraphs of the discussion section are intended to contextualize the result and provide an insight to how important this site is. We also want to highlight the reason why the site is used in this study as well as show how a site with this level of available information can be used to develop methods that can be used elsewhere as well. This is the reason why we consider starting the discussion as we have done is the best way for this paper.

Supplement:
- The color red often is used to indicate erroneous data. Maybe pick a different color to represent the enamel only value. It is true, however, here we are using it to match the colour code in the main text and we have clearly stated in the figure caption what it represents. We feel that it is best to leave as it is.

- Match font type, heading colors, and general styles to the plosone format.

Done

Reviewer #2: This revised manuscript takes account of the reviewer comments and is substantially improved over the original submission. I see no reason it should not now be published. Alex Pryor.

Thanks!
